# Synthesis of new dihydroberberine and tetrahydroberberine analogues and evaluation of their antiproliferative activity on NCI-H1975 cells

**DOI:** 10.3762/bjoc.16.133

**Published:** 2020-07-06

**Authors:** Giacomo Mari, Lucia De Crescentini, Serena Benedetti, Francesco Palma, Stefania Santeusanio, Fabio Mantellini

**Affiliations:** 1 Section of Chemistry and Pharmaceutical Technologies, Department of Biomolecular Sciences, University of Urbino “Carlo Bo”, Via I Maggetti 24, 61029 Urbino, Italy; 2 Section of Biochemistry and Molecular Biology, Department of Biomolecular Sciences, University of Urbino “Carlo Bo”, Via Saffi 2, 61029 Urbino, Italy

**Keywords:** antiproliferative agent, dihydroberberine, hydrazones, reduction, tetrahydroberberine

## Abstract

Dihydroberberine (DHBER), the partially reduced form of the alkaloid berberine (BER), is known to exhibit important biological activities. Despite this fact, there have been only few studies that concern the biological properties of functionalized DHBER. Attracted by the potentiality of this latter compound, we have realized the preparation of new arylhydrazono-functionalized DHBERs, starting from BER and some α-bromohydrazones. On the other hand, also the fully reduced form of BER, namely tetrahydroberberine (THBER), and its derivatives have proven to present different biological activities. Therefore, the obtained arylhydrazono-functionalized DHBERs were reduced to the corresponding arylhydrazono-THBERs. The antiproliferative activity of both arylhydrazono-DHBERs and -THBERs has been evaluated on NCI-H1975 lung cancer cells.

## Introduction

The rhizome of *Coptis chinensis Franch.* is a common remedy in traditional oriental medicine for the treatment of various inflammatory diseases. The main component of this rhizome is berberine (BER), an alkaloid with numerous pharmacological properties, which include anticancer and anti-inflammatory activities [1]. However, the low bioavailability, poor solubility, and moderate nucleic acid binding affinity constitute a severe limitation in the employ of BER [2–4].

To minimize these drawbacks, its isoquinoline portion was derivatized in positions 9 and 13 that are critical for topoisomerase inhibition and quadruplex structure binding [5–8]. The insertion of a phenyl group or a benzhydryl group linked to position 13 of the BER skeleton, usually causes a geometric propensity for additional stacking-type, noncovalent aromatic interactions with cellular targets forming stronger complexes with nucleic acids than BER [9–12]. In fact, the so obtained functionalized BER shows better anticancer activity [13–19], and an increased DNA and RNA binding efficacy [4,6,9], due to its aromatic interactions with the biological macromolecules [20].

Another interesting and promising derivative is dihydroberberine (DHBER), the reduced form of BER. The enaminic function of this alkaloid is a precious reactive site, employed to insert various electrophilic agents in position 13 of the DHBER skeleton [21–24]. Usually, to remedy the poor stability of the DHBER-derivatives obtained, they are directly oxidized to the corresponding functionalized BERs, relegating to DHBERs the role of intermediates. However, pharmacokinetic studies have shown that, compared with BER [25], DHBER displayed improved adsorption and enhanced bioavailability, as also confirmed by several papers that describe how the gut microbiota converts BER derivatives into its absorbable form DHBER, which has an intestinal absorption rate 5-fold higher than that of BER [26–28].

Currently, DHBER is a drug candidate for the treatment of type 2 diabetes [29–30], can reduce the atherosclerotic plaque size [31], has therapeutic potential for myotonic dystrophy type I due to its central nervous system effects [32], inhibits both hERG current and the expression of hERG protein [33], inhibits the pancreatic lipase [34], shows antiradical, revitalizing and antifibrotic properties for dermatological applications [35], manifests a synergic effect with antibiotics [36], and displays antitumoral activities [37–38].

Unlike BER, there have been few studies that concern the biological properties of functionalized DHBER. Attracted by the potentiality of this latter compound, we have realized the preparation of new arylhydrazono-functionalized DHBERs that were tested in vitro for their biological activity; in particular, we focused our attention on their antiproliferative capacity, due to our previous research experiences on the anticancer properties of both natural and synthetic molecules [39–42]. A common strategy to increase the biological activity of a class of products can be the insertion in the original molecule of different functions with their own peculiar features. Often, the biological properties of the resultant derivatives are not simply attributable to the sum of the characteristics shown by the individual moieties, but synergistic effects can increase their effectiveness, or induce the manifestation of new features. The choice to insert an hydrazonic function on the DHBER skeleton was suggested by its wide range of interesting biological activities like antimicrobial [43], antioxidant [44], analgesic [45], anti-inflammatory [45], antiplatelet [45], anticonvulsant [46], antiprotozoal [47], antidiabetic [48], and antitubercular [49]. Relevant is the role of the hydrazone moiety as antitumor agent [50–53]. An interesting example reported by Ferreira demonstrates that the chemical derivatization of the indole alkaloids dregamine and tabernaemontanine to yield new hydrazone derivatives enhances the apoptosis inducing activity [54].

**Scheme 1:**
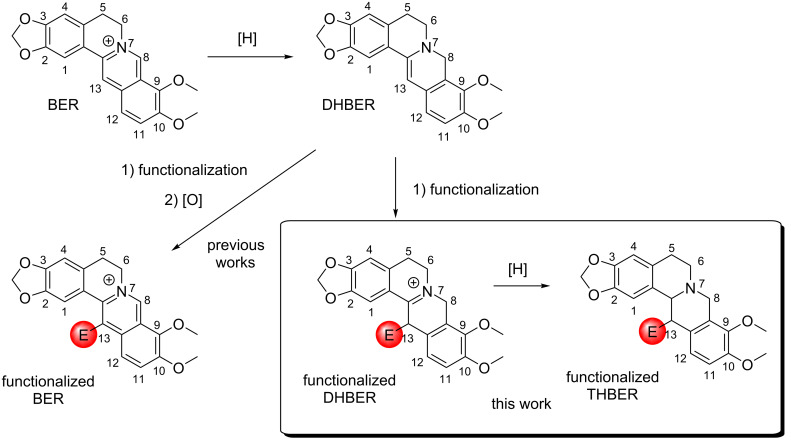
Preparation of functionalized-BER, -DHBER and -THBER derivatives.

Another particularly useful feature of the hydrazone group is related by its high tendency to provide solid derivatives. The target of the here reported strategy is to recover as pure products the desired functionalized DHBERs to evaluate their antiproliferative activity directly by precipitation from the reaction medium, avoiding any possible and common subsequent side reactions for this substrate during the purification processes (Scheme 1).

On the other hand, also the fully reduced form of BER, namely tetrahydroberberine (THBER) or canadine, is an isoquinoline alkaloid recurring in several plant species [55–57]. THBER is able to act to the central nervous system as an inhibitor [58], possesses hepatoprotective effects [59], is employed in the protection against cerebral ischemia-reperfusion injury [60], and shows anti-arrhythmic activity [61]. Furthermore, THBER and its derivatives are effective antioxidant agents [62–65].

To extend our investigations in this field, the obtained arylhydrazono-functionalized DHBERs were reduced to the corresponding arylhydrazono-THBERs, whose antiproliferative properties have been evaluated (Scheme 1).

## Results and Discussion

### Chemistry

The unsubstituted DHBER was prepared by treating BER with 2.5 equivalents of NaBH_4_ in pyridine at room temperature [66–67], and employed in the reaction with α-bromohydrazone **1a** chosen as the representative model, to determine the optimal reaction conditions for the synthesis of the corresponding arylhydrazono-functionalized DHBER **2a**. Different solvents, temperatures and molar ratios were investigated (Table 1).

Dichloromethane (DCM), room temperature, and an equimolar ratio have proved to be the best conditions, since the desired product **2a** was achieved as a pure pale yellow solid directly from the reaction medium with the best yields (Table 1, entry 7). In tetrahydrofuran (THF), in ethyl acetate (EtOAc), and in alcohols such as methanol, ethanol, isopropanol (MeOH, EtOH, iPrOH) the reaction furnishes a complicate profile, where the desired **2a** is detectable only in traces (Table 1, entries 1–5). The monitoring by TLC of the reaction conducted in acetonitrile reveals the formation of derivative **2a**, which, however, does not precipitate in the reaction medium (Table 1, entry 6).

**Table 1:** Reaction conditions optimization.

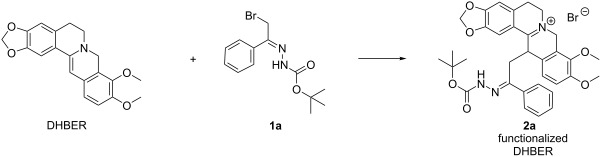				

Entry^a^	Solvent	*T* (°C)	Molar ratio (DHBER/**1a**)	Yield of **2a** (%)^b^

1	THF	Rt	1:1	–^c^
2	EtOAc	rt	1:1	–^c^
3	MeOH	rt	1:1	–^c^
4	EtOH	rt	1:1	–^c^
5	iPrOH	rt	1:1	–^c^
6	ACN	rt	1:1	–^d^
**7**	**DCM**	**rt**	**1:1**	**66**
8	DCM^e^	rt	1:1	–^f^
9	DCM	rt	1:2	–^c^
10	DCM	0	1:1	25
11	DCM	reflux	1:1	54

^a^The reactions were conducted on 0.3 mmol scale in 1.0 mL of solvent. ^b^Isolated yields. ^c^Complicated mixture. ^d^**2a** is detectable on TLC, but it does not precipitate from the reaction medium. The chromatographic separation does not furnish **2a**, probably due to degradation processes that occur in the column. ^e^The reactions were conducted on 0.3 mmol scale in 0.5 mL of solvent. ^f^The starting materials DHBER and **1a** are not completely dissolved.

The spontaneous precipitation is particularly relevant to prevent any further degradation process. In fact, all attempts to obtain the pure **2a** by chromatographic process failed, employing silica gel or aluminum oxide or basified silica gel (by addition of 3% of triethylamine).

**Scheme 2:**
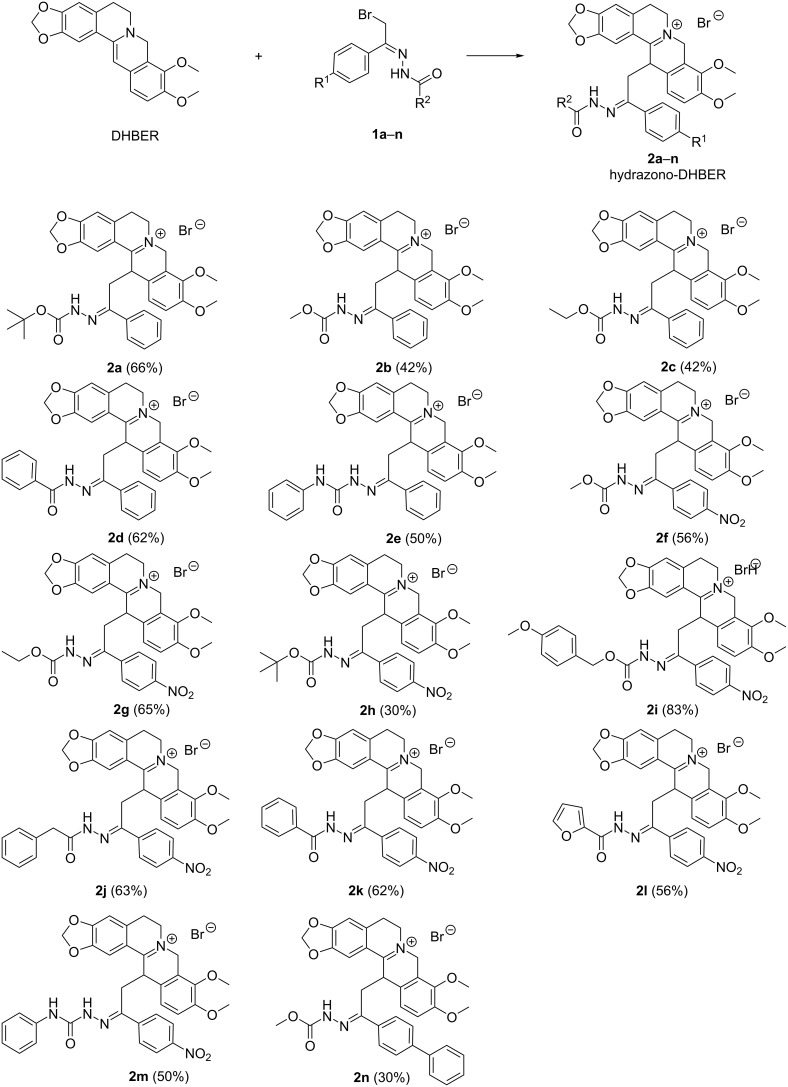
Substrate scope of hydrazono-DHBER. Reaction conditions: DHBER (1.0 mmol), **1** (1.0 mmol), DCM 3.0 mL, 25 °C. Isolated yields in parentheses.

Having identified the optimal conditions, the reaction scope was enlarged employing the α-bromohydrazones **1a–n** (Scheme 2).

All the hydrazono-DHBERs **2a–n** directly precipitate as pure products in the reaction medium with yields ranging from poor to good (30–83%). Probably the low affinity of the iminium and hydrazone groups of compounds **2a–n** with a medium-low polarity solvent such as DCM causes their direct precipitation.

**Figure 1:**
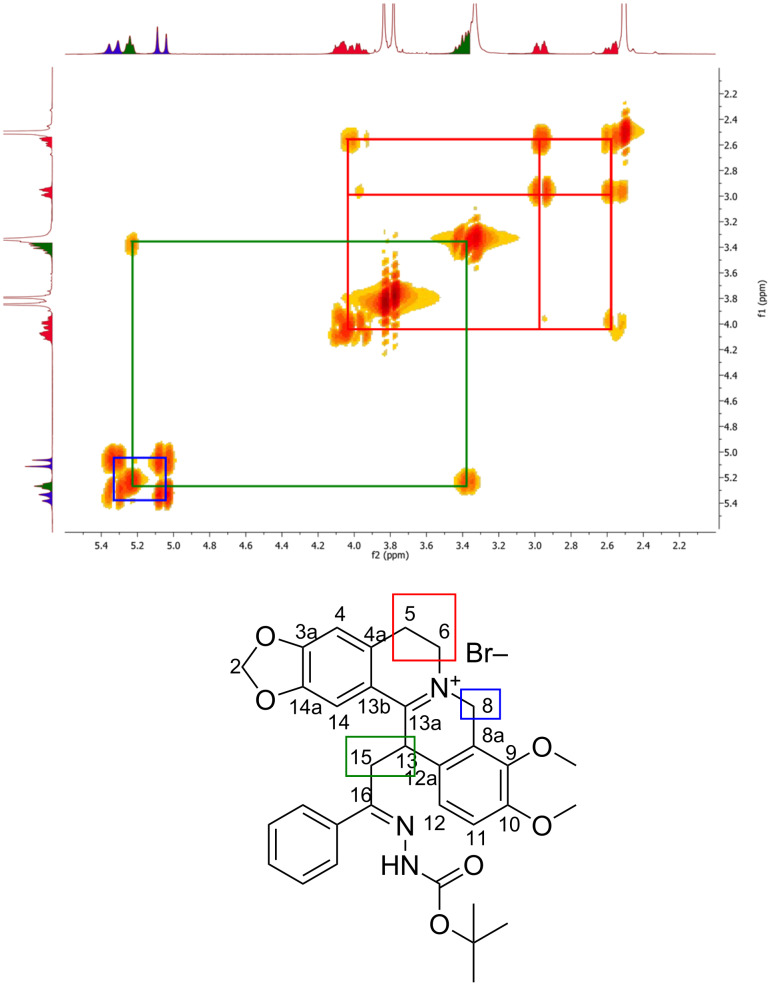
^1^H,^1^H-COSY spectrum of DHBER **2a** [65].

The mono- and two-dimensional NMR studies confirm that hydrazono-DHBER **2a–n** are in the iminium-tautomeric form. For example, the COSY experiment of hydrazono-DHBER **2a** clearly indicates the presence of three sets of related signals: the system with the triplet at 5.24 ppm, attributable to the proton bound to the carbon 13, coupled with the multiplet at 2.54–2.60 ppm, attributable to the methylene in position 15, is diagnostic for the structure proposed (highlighted in green, Figure 1). A second set, two doublets at 5.06 and 5.32 ppm, is attributable to the diastereotopic protons bound to carbon in position 8 (highlighted in blue, Figure 1). The last group, three multiplets at 2.54–2.60, 2.93–2.99 and 3.93–4.10 ppm, is ascribable to the protons of the carbons 5 and 6, respectively.

On the other hand, we have recently demonstrated that some structurally complex THBER analogues, namely pyrrolino-tetrahydroberberines, synthesized by some of us [68], exhibited enhanced antioxidant properties in comparison to THBER against a wide variety of pathophysiologically relevant oxidants such as peroxyl radicals, ferrous ion, and hydrogen peroxide [65].

**Scheme 3:**
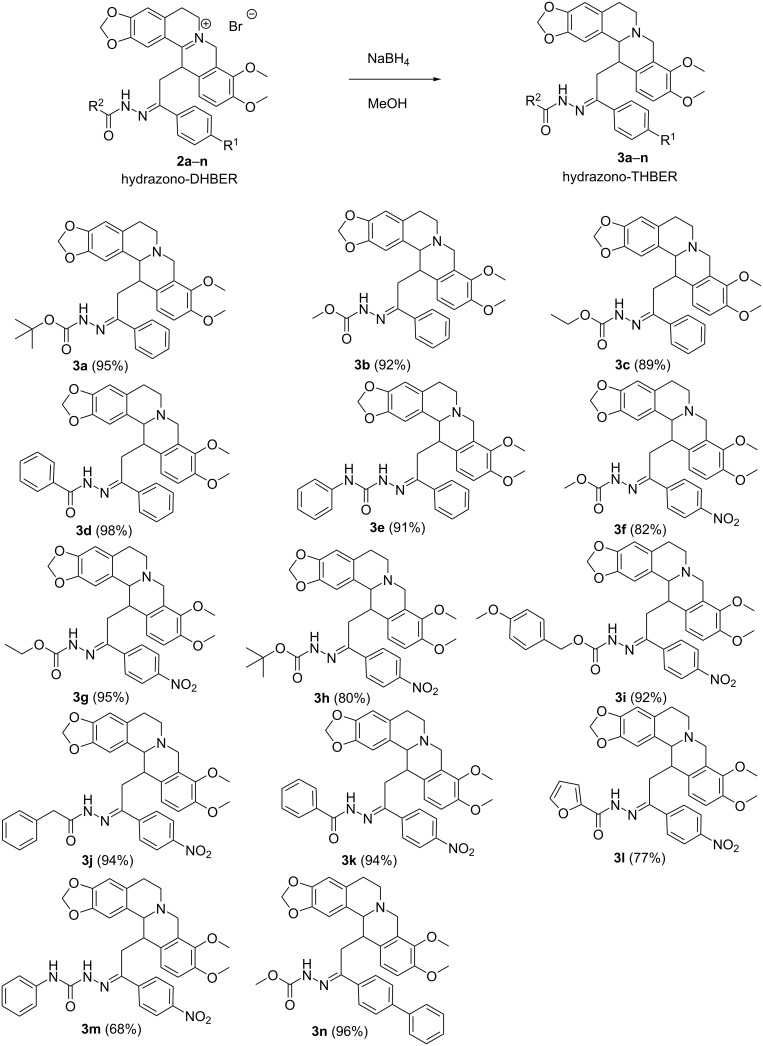
Synthesis of hydrazono-THBERs **3a–n**. Reaction conditions: DHBER (0.5 mmol), NaBH_4_ (2.0 mmol), MeOH, 3.0 mL, 25 °C. Isolated yields in parentheses.

In continuation of our ongoing interest, the hydrazono-DHBERs **2a–n** were further treated with 2.0 equivalents of sodium boron hydride at room temperature in methanol. The reaction furnishes the corresponding hydrazono-THBERs **3a–n** in good yields (77–96%, Scheme 3).

**Figure 2:**
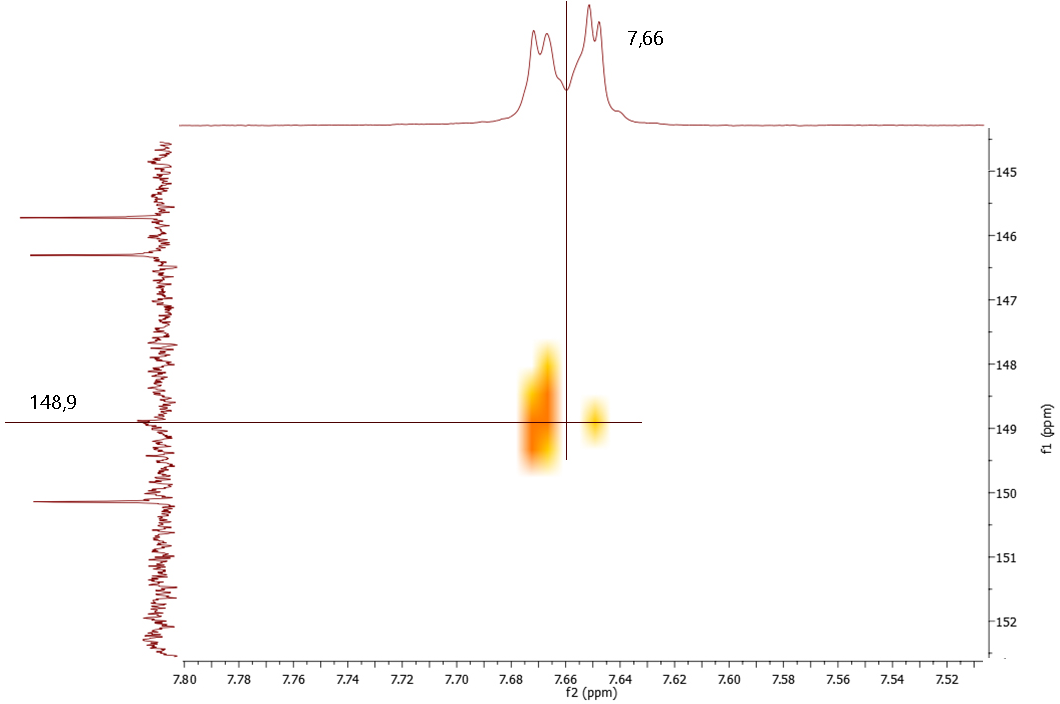
Detail of the hydrazone signal in the HMBC spectrum of THBER **3a**.

It is noteworthy that in these conditions, only the iminium moiety is reduced, leaving unchanged the hydrazone function. The presence of the hydrazone moiety is confirmed by the HMBC spectrum in which the signal at 148.9 ppm coupled with the double doublet at 7.66 ppm is attributable to the protons in *ortho* position of the phenyl bound to the carbon 16 of THBER **3a** (Figure 2).

**Figure 3:**
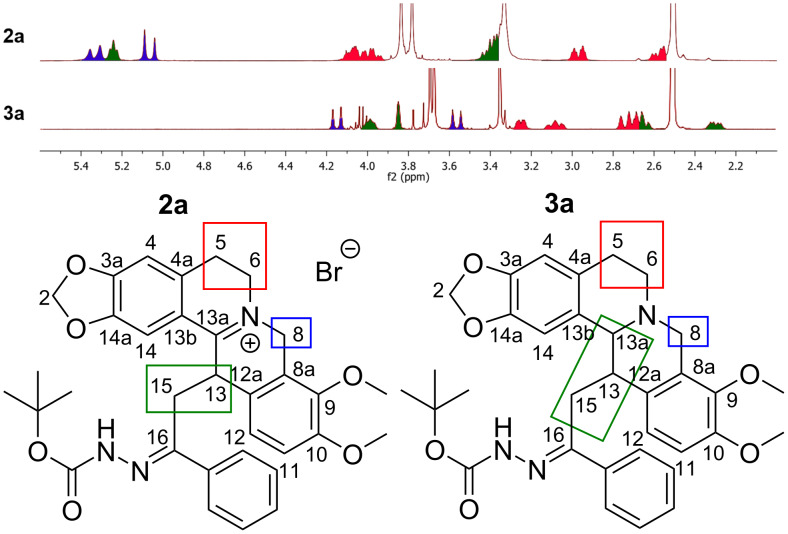
Comparison between ^1^H NMR spectra of DHBER **2a** and of THBER **3a**.

From the comparison between the ^1^H NMR spectra of DHBER **2a** and THBER **3a**, it can also be noted as the chemical shifts of 6-C, and 8-C protons, which are the closest to the N-7 nitrogen atom are significantly shifted downfield (assigned by 2D-NMR analysis, see Supporting Information File 1). For example, the two doublets at 5.06 and 5.36 ppm, assigned to the C8 protons of DHBER **2a**, are shifted to 3.55 and 4.15 ppm, respectively in THBER **3a** (signals highlighted in blue, Figure 3). Similar considerations can be extended to protons bound to C5, C6, (signals highlighted in red, Figure 3), and to protons of 13a-C, 13-C and 15-C (signals highlighted in green, Figure 3).

It is noteworthy that in the reduction process of the DHBER **2** precursors, a single diastereoisomer of the corresponding THBERs **3** is produced according to ^1^H and ^13^C NMR data.

**Figure 4:**
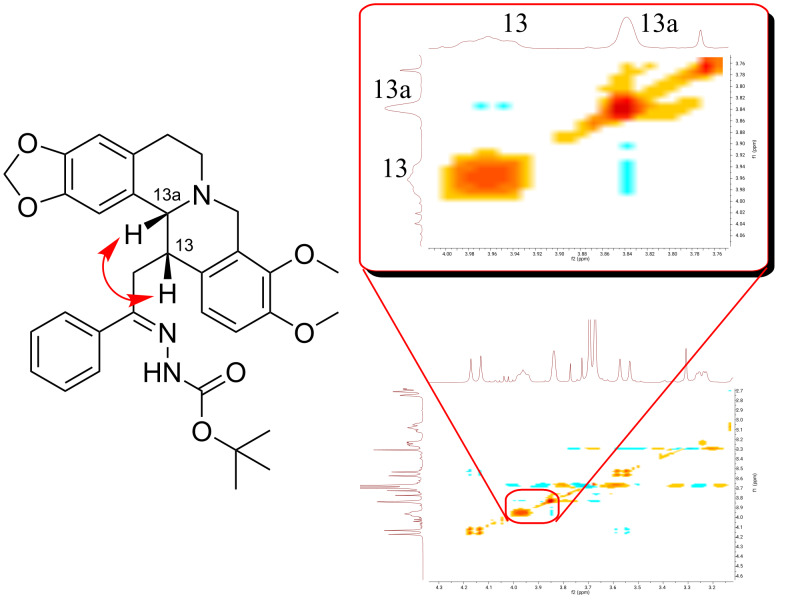
NOE correlation between C13−H and C13a−H of THBER **3a**.

In particular, the NOE correlation between C13−H and C13a−H of THBERs **3a** chosen as representative example, reveals that these two hydrogens are in *cis* position (Figure 4). Therefore, the THBERs **3** show the same configuration of the metabolite cavidine that is the 13-methyltetrahydroprotoberberine alkaloid which occur in various species of *Corydalis* [69].

### Evaluation of the antiproliferative activity of hydrazono-DHBERs **2** and hydrazono-THBERs **3**

**Figure 5:**
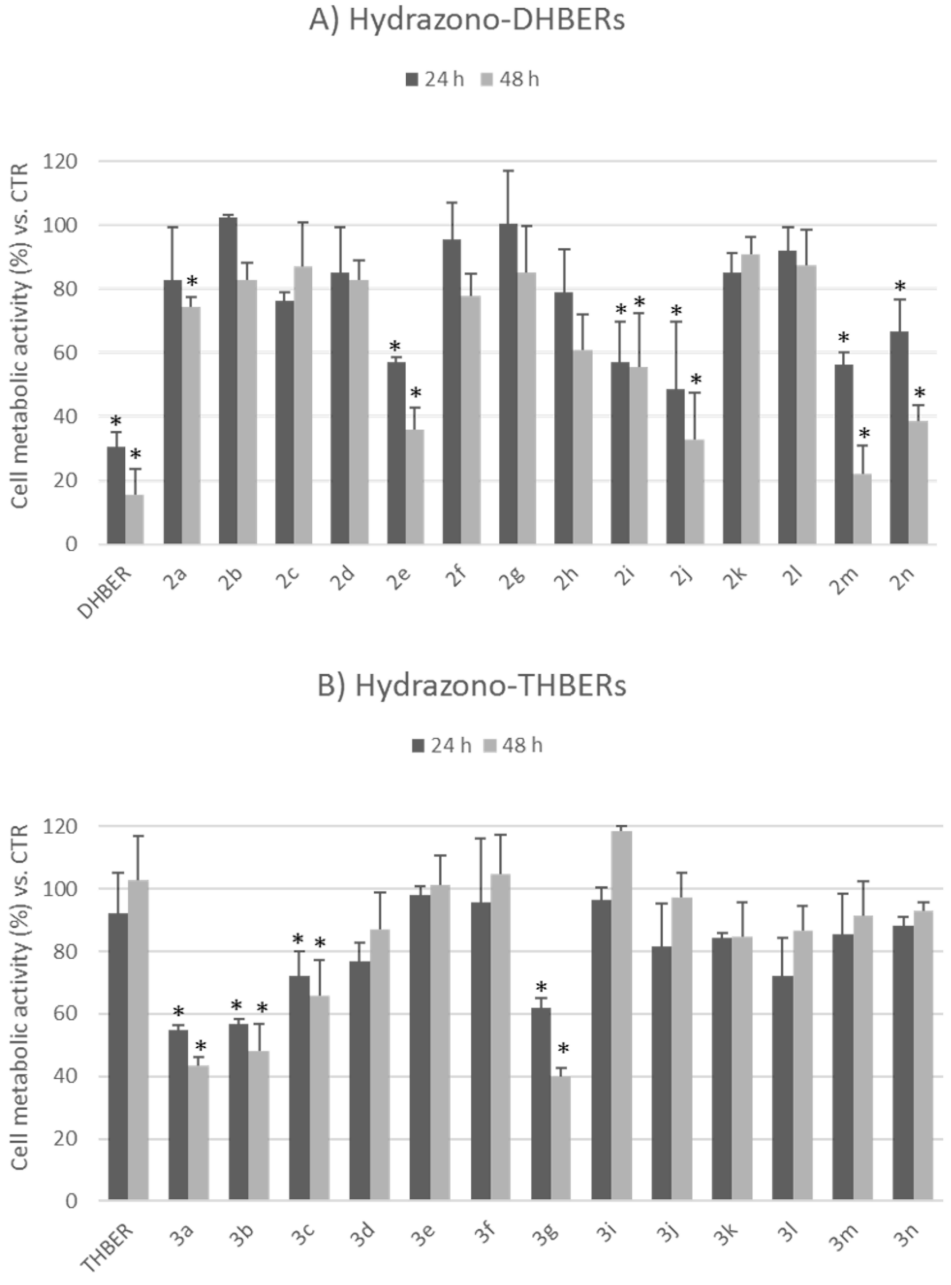
Effects of 25 µM hydrazono-DHBERs **2a–n** (A) and hydrazono-THBERs **3a–g,i–n** [70] (B) on NCI-H1975 cell proliferation as evaluated by WST-8 assay (estimating cell metabolic activity) at 24 h and 48 h of treatment. Data are expressed as mean ± SD (*n* = 3). *p < 0.05 vs CTR (DMSO 0.05%).

**Figure 6:**
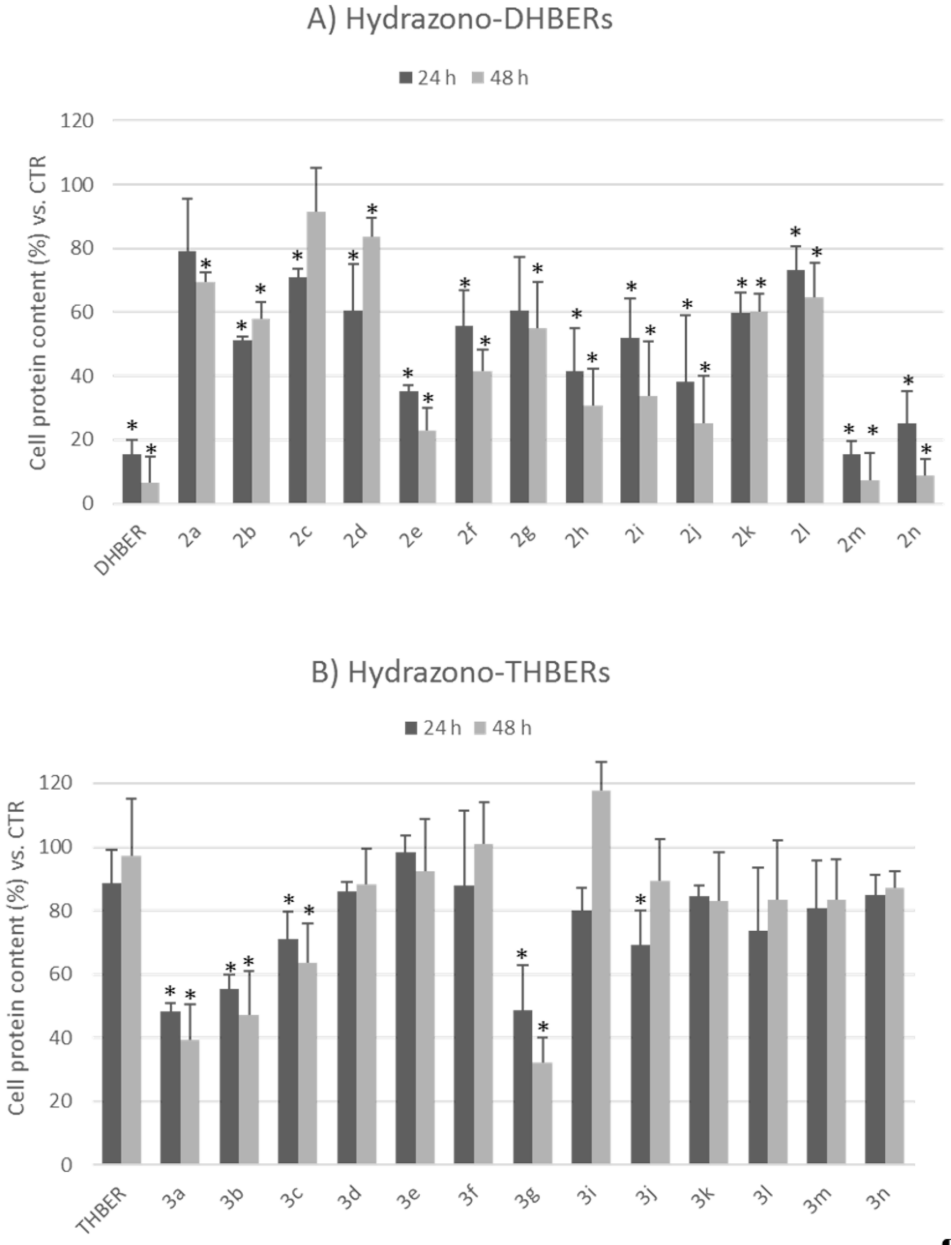
Effects of 25 µM hydrazono-DHBERs **2a–n** and hydrazono-THBERs **3a–g,i–n** [70] on NCI-H1975 cell proliferation as evaluated by SRB assay (estimating total protein content) at 24 h and 48 h of treatment. Data are expressed as mean ± SD (*n* = 3). *p < 0.05 vs CTR (DMSO 0.05%).

As regards the in vitro experiments on lung cancer cells, two different tests were applied to evaluate the effects of hydrazono-DHBERs **2a–n** and hydrazono-THBERs **3a–g,i–n** [70] on the NCI-H1975 cell proliferation. Both WST-8 [2-(2-methoxy-4-nitrophenyl)-3-(4-nitrophenyl)-5-(2,4-disulfophenyl)-2*H*-tetrazolium, monosodium salt] (Figure 5) and SRB (sulforhodamine B) (Figure 6) assays revealed that DHBER significantly reduced the cancer cell proliferation as compared to untreated cells both at 24 and 48 h of incubation, accordingly to previous findings on cultured tumor cells. On the contrary, THBER did not affect the NCI-H1975 viability. Among hydrazono-DHBERs, **2m** and **2n** showed the highest antiproliferative properties, quite comparable to those of DHBER. Referring to hydrazono-THBERs, **3a**, **3b**, and **3g** were the most effective in significantly reducing cancer cell proliferation as compared to THBER and untreated control cells. Overall, hydrazono-DHBERs presented a higher antiproliferative capacity than hydrazono-THBERs, making these compounds particularly of interest from a chemical and biological point of view.

## Conclusion

In conclusion, here we report a novel simple, and convenient methodology for easy access to a series of arylhydrazone-functionalized dihydroberberines and tetrahydroberberines. Both the procedures proceed rapidly to completion, under mild reaction conditions, requiring readily available and inexpensive starting materials.

The purification of the hydrazono-dihydroberberines by sole filtration, avoiding any solvent-consuming steps, such as chromatography, extraction or recrystallization, determines a very simple workup. This aspect, together with the total efficiency in atom economy observed, makes this procedure advantageous from both the environmental and the economic point of view.

Also, the regioselective reduction of the iminium moiety that leaves unchanged the hydrazono function requires a simple treatment of the hydrazone dihydroberberines with sodium borohydride producing a new class of tetrahydroberberine analogues with interesting biological properties. Indeed, the investigation on the antiproliferative effects highlighted the ability of some hydrazono-DHBERs and hydrazono-THBERs to induce a significant growth inhibition on the tested cancer cell line, which might deserve future researches.

## Experimental

**General experimental details:** All chemicals and solvents were purchased from commercial suppliers and used as received. α-Bromohydrazones [71] and DHBER [66–67] were prepared as previously reported. Melting points were determined in open capillary tubes and are uncorrected. FTIR spectra were obtained as nujol mulls. All ^1^H NMR and ^13^C NMR spectra were recorded at 400 and 100 MHz, respectively. Proton and carbon spectra were referenced internally to solvent signals, using values of δ = 2.50 ppm for proton (middle peak) and δ = 39.50 ppm for carbon (middle peak) in DMSO-*d*_6_ and δ = 7.27 ppm for proton and δ = 77.00 ppm for carbon (middle peak) in CDCl_3_. All coupling constants (*J*) are given in Hz. All the NH exchanged with D_2_O. Precoated aluminium oxide plates 0.25 mm were employed for analytical thin-layer chromatography. All new compounds showed satisfactory elemental analysis. Mass spectra were recorded in the ESI and EI modes. The nomenclature was generated using ACD/IUPAC Name (version 3.50, 5 Apr. 1998), Advanced Chemistry Development Inc., Toronto, ON (Canada).

**General procedure for the synthesis of hydrazono-dihydroberberines (DHBERs) 2a–n.** To a solution in dichloromethane (3.0 mL) of α-bromohydrazones **1a–n** [71] (1.0 mmol) was added the unsubstitued dihydroberberine [66–67] (1.0 mmol). The reaction was allowed to stand at room temperature under magnetic stirring until the complete disappearance of the starting materials (TLC monitoring) and the formation of compounds **2a–n** that directly precipitated from the reaction medium. Compounds **2a–n** were collected by filtration under vacuum and washed with acetone (5.0 mL). Characterization data of **2a**, chosen as representative compound, are given below.

**13-(2-(2-(*tert*-Butoxycarbonyl)hydrazono)-2-phenylethyl)-9,10-dimethoxy-5,6,8,13-tetrahydro-[1,3]dioxolo[4,5-*g*]isoquinolino[3,2-*a*]isoquinolin-7-ium bromide (2a): 2a** was isolated by precipitation in the reaction medium (DCM) in 66% yield (428 mg). Pale yellow amorphous solid; mp: 123–124 °C; ^1^H NMR (400 MHz, DMSO-*d*_6_, 25 °C) δ 1.45 (s, 9H, C(*CH_3_*)*_3_*), 2.54–2.60 and 2.93–2.99 (2m, 2H, C(5*)H_2_*), 3.36–3.43 (m, 2H, C(15)*H_2_*), 3.78 (s, 3H, OC*H_3_*), 3.83 (s, 3H, OC*H_3_*), 3.93–4.10 (m, 2H, C(6)*H_2_*), 5.06 (d, *J* = 19.6 Hz, 1H, C(8)*H_2_*), 5.24 (t, *J* = 6.8 Hz, 1H, C*H*), 5.32 (d, *J* = 19.6 Hz, 1H, C(8)*H_2_*), 6.25 and 6.27 (2s, 2H, OC(2)*H_2_*O), 7.05 (s, 1H, C(14)*H*), 7.17 (s, 2H, C(12)*H* and C(11)*H*), 7.29–7.38 (2m, 3H, Ar*H*), 7.47–7.49 (2m, 2H, Ar*H*), 7.61 (s, 1H, C(4)*H*), 9.71 (s, 1H, N*H*); ^13^C NMR (100 MHz, DMSO-*d*_6_, 25 °C) δ 24.7 (t), 27.9 (q), 31.7 (t), 38.1 (d), 50.5 (t), 52.6 (t), 56.0 (q), 60.3 (q), 79.6 (s), 103.1 (t), 108.4 (d), 108.9 (d), 113.6 (d), 118.7 (s), 122.5 (d), 123.0 (s), 123.5 (s), 126.2 (d), 128.2 (d), 128.9 (d), 136.5 (s), 137.0 (s), 143.6 (s), 147.1 (s), 151.5 (s), 152.6 (s), 154.3 (s), 164.0 (s), 172.1 (s); IR (nujol): ν_max_ = 3196, 3039, 1738, 1727, 1700 cm^−1^; ESIMS (*m/z*): [M − Br]^+^ 570; anal. calcd. for C_33_H_36_BrN_3_O_6_ (650.56): C, 60.92; H, 5.58; N, 6.46; found: C, 61.04; H, 5.54; N, 6.40.

**General procedure for the synthesis of hydrazono-tetrahydroberberine (THBERs) 3a–n:** To a solution of hydrazono-DHBERs **2a–n** (0.2 mmol) in methanol (2.0 mL) at room temperature sodium borohydride (0.4 mmol) was added. The reaction was allowed to stand at room temperature under magnetic stirring until the complete disappearance of the starting hydrazono-DHBERs **2a–n** (2.0–3.0 h, TLC monitoring). The reaction solvent was then evaporated under reduced pressure. The crude mixture was then purified by column chromatography on silica gel (elution with cyclohexane/ethyl acetate mixtures) to afford products **3a–n** that were crystallized in a mixture of diethyl ether/petroleum ether. Characterization data of **3a**, chosen as representative compound, are given below.

***tert*-Butyl 2-(2-(9,10-dimethoxy-6,8,13,13a-tetrahydro-5*H*-[1,3]dioxolo[4,5-*g*]isoquinolino[3,2-*a*]isoquinolin-13-yl)-1-phenylethylidene)hydrazinecarboxylate (3a): 3a** was isolated by chromatographic column on silica gel (ethyl acetate/cyclohexane, 30:70) in 95% yield (109 mg). White amorphous solid; mp: 162–164 °C; ^1^H NMR (400 MHz, DMSO-*d*_6_, 25 °C) δ 1.45 (s, 9H, C(C*H_3_)_3_*), 2.29–2.34 (m, 1H, C(15)*H_2_*), 2.62–2.75 (m, 3H, C(15)*H_2_,* C(5)*H2,* C(6)*H_2_*), 3.04–3.12 (m, 1H, C(5)*H_2_*), 3.23–3.26 (m, 1H, C(6)*H_2_*), 3.55 (d, *J* = 15.6 Hz, 1H, C(8*)H_2_*), 3.67 (s, 3H, O*CH_3_*), 3.70 (s, 3H, OC*H_3_*), 3.84 (brs, 1H, C(13a)*H*), 3.94–3.98 (m, 1H, C(13)*H*), 4.15 (d, *J* = 16.0 Hz, 1H, C(8)*H_2_*), 5.99 and 6.00 (2s, 2H, OC(2)*H_2_*O), 6.41 (d, *J* = 8.4 Hz, 1H, C(12)*H*), 6.57 (d, *J* = 8.8 Hz, 1H, C(11)*H*), 6.75 (s, 1H, C(4)*H*), 7.03 (s, 1H, C(14)*H*), 7.32–7.38 (m, 3H, Ar*H*), 7.63–7.65 (m, 2H, Ar*H*), 11.04 (brs, 1H, N*H*); ^13^C NMR (100 MHz, DMSO-*d*_6_, 25 °C) δ 28.0 (q), 28.6 (t), 29.1 (t), 39.9 (d), 50.0 (t), 53.1 (t), 55.4 (q), 59.4 (q), 63.4 (d), 79.1 (s), 100.7 (t), 106.1 (d), 108.1 (d), 110.2 (d), 125.2 (d), 126.0 (d), 127.4 (d), 127.8 (s), 128.0 (d), 128.4 (s), 128.7 (s), 129.1 (s), 138.2 (s), 143.8 (s), 145.7 (s), 146.3 (s), 148.9 (s), 150.1 (s), 152.3 (s); IR (nujol): ν_max_ = 3288, 3129, 1742 cm^−1^; HRMS–ESI (*m*/*z*): [M + H]^+^: calcd. for C_33_H_38_N_3_O_6_, 572.2761; found, 572.2814.

## Supporting Information

File 1 Experimental procedures, characterization data, and copies of NMR spectra for compounds **2a–n** and **3a–n**.

File 2 Antiproliferative evaluation of compounds **2a–n** and **3a–g,i–n**.
