# Supplementary material for: Synthesis of new dihydroberberine and tetrahydroberberine analogues and evaluation of their antiproliferative activity on NCI-H1975 cells
Source: Beilstein J Org Chem. 2020 Jul 6;16:1606–16. doi: 10.3762/bjoc.16.133 (PMC7356317; doi:10.3762/bjoc.16.133)
Supplement: File 1 — Experimental procedures, characterization data, and copies of NMR spectra for compounds 2a–n and 3a–n. [file Beilstein_J_Org_Chem-16-1606-s001.pdf]

BEILSTEIN JOURNAL OF ORGANIC CHEMISTRY

Supporting Information

for

Synthesis of new dihydroberberine and tetrahydroberberine analogues and evaluation of their antiproliferative activity on NCI-H1975 cells

Giacomo Mari, Lucia De Crescentini, Serena Benedetti, Francesco Palma,
Stefania Santeusanio and Fabio Mantellini

Beilstein J. Org. Chem. **2020,** *16,* 1606–1616. doi:10.3762/bjoc.16.133

Experimental procedures, characterization data, and copies of NMR spectra for compounds 2a–n and 3a–n

License and Terms: This is a supporting information file under the terms of the Creative Commons Attribution License (http://creativecommons.org/licenses/by/4.0). Please note that the reuse, redistribution and reproduction in particular requires that the authors and source are credited.
The license is subject to the *Beilstein Journal of Organic Chemistry* terms and conditions: (https://www.beilstein-journals.org/bjoc)

Table of contents

1. General experimental details S2

2. Experimental procedures and characterization data S2–24

    2.1. General procedure for the synthesis of hydrazono-dihydroberberines (DHBERs) **2a–n** S2

    2.2. Characterization data of hydrazono-dihydroberberines (DHBERs) **2a–n** S3

    2.3. General procedure for the synthesis of hydrazono-tetrahydroberberines (THBERs) **3a–n** S13

    2.4. Characterization data of hydrazono-tetrahydroberberines (THBERs) **3a–n** S13

3. ^{1}H and ^{13}C NMR spectra of products **2a–n** S25–39

4. ^{1}H and ^{13}C NMR spectra of products **3a–n** S40–61

5. References S62

1. General experimental details.

All chemicals and solvents were purchased from commercial suppliers and used as received. α-Bromo hydrazones[1] and DHBER[2,3] were prepared as previously reported. Melting points were determined in open capillary tubes and are uncorrected. FTIR spectra were obtained as Nujol mulls. All ^{1}H NMR and ^{13}C NMR spectra were recorded at 400 and 100 MHz, respectively. Proton and carbon spectra were referenced internally to solvent signals, using values of $\delta = 2.50$ ppm for proton (middle peak) and $\delta = 39.50$ ppm for carbon (middle peak) in DMSO-d_6 and $\delta = 7.27$ ppm for proton and $\delta = 77.00$ ppm for carbon (middle peak) in $CDCl_3$. All coupling constants (*J*) are given in Hz. All the NH exchanged with D_2O. Precoated aluminium oxide plates 0.25 mm were employed for analytical thin-layer chromatography. All new compounds showed satisfactory elemental analysis. Mass spectra were recorded in the ESI and EI modes. The nomenclature was generated using ACD/IUPAC Name (version 3.50, 5 Apr. 1998), Advanced Chemistry Development Inc., Toronto, ON (Canada).

2. Experimental procedures and characterization data

2.1. General Procedure for the Synthesis of Hydrazono-dihydroberberines (DHBERs) 2a–n.

To a solution in dichloromethane (3.0 mL) of α-bromo hydrazones **1a–n**[1] (1.0 mmol) was added the unsubstituted dihydroberberine[2,3] (1.0 mmol). The reaction was allowed to stand at room temperature under magnetic stirring until the complete disappearing of the starting materials (TLC monitoring) and the formation of compounds **2a–n** that directly precipitated from the reaction medium. Compounds **2a–n** were collected by filtration under vacuum and washed with acetone (5.0 mL).

2.2. Characterization data of Hydrazono-dihydroberberines (DHBERs) 2a–n.

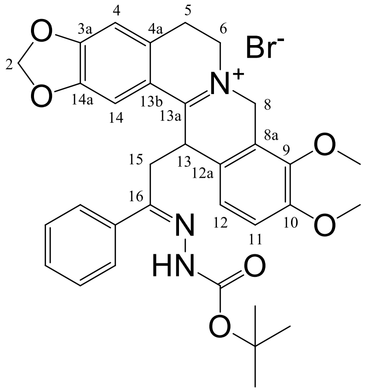

13-(2-(2-(*tert*-Butoxycarbonyl)hydrazono)-2-phenylethyl)-9,10-dimethoxy-5,6,8,13-tetrahydro-[1,3]dioxolo[4,5-*g*]isoquinolino[3,2-*a*]isoquinolin-7-ium bromide (2a).

2a was isolated by precipitation in the reaction medium (DCM) in 66% yield (428 mg). Pale yellow amorphous solid; mp: 123–124 °C; ^{1}H NMR (400 MHz, DMSO$_{d6}$, 25 °C): 1.45 (s, 9H, C(*CH$_3$)$_3$*), 2.54–2.60 and 2.93–2.99 (2m, 2H, *C(5)H$_2$*), 3.36–3.43 (m, 2H, *C(15)H$_2$*), 3.78 (s, 3H, O*CH$_3$*), 3.83 (s, 3H, O*CH$_3$*), 3.93–4.10 (m, 2H, C(6)*H$_2$*), 5.06 (d, 1H, *J* =19.6 Hz, *C(8)H$_2$*), 5.24 (t, 1H, *J* =6.8 Hz, *C(13)H*), 5.32 (d, 1H, *J* =19.6 Hz, C(8)*H$_2$*), 6.25 and 6.27 (2s, 2H, OC(2)*H$_2$*O), 7.05 (s, 1H, *C(14)H*), 7.17 (s, 2H, *C(12)H* and *C(11)H*), 7.29–7.38 (2m, 3H, *ArH*), 7.47–7.49 (2m, 2H, *ArH*), 7.61 (s, 1H, *C(4)H*), 9.71 (s, 1H, *NH*); ^{13}C NMR (100 MHz, DMSO$_{d6}$, 25 °C): δ = 24.7 (t), 27.9 (q), 31.7 (t), 38.1 (d), 50.5 (t), 52.6 (t), 56.0 (q), 60.3 (q), 79.6 (s), 103.1 (t), 108.4 (d), 108.9 (d), 113.6 (d), 118.7 (s), 122.5 (d), 123.0 (s), 123.5 (s), 126.2 (d), 128.2 (d), 128.9 (d), 136.5 (s), 137.0 (s), 143.6 (s), 147.1 (s), 151.5 (s), 152.6 (s), 164.0 (s), 154.3 (s), 172.1 (s); IR (nujol): ν_{max} = 3196, 3039, 1738, 1727, 1700 cm^{-1}; MS *m/z* (ESI): 570 [M − Br]$^+$; anal. calcd. for $C_{33}H_{36}BrN_3O_6$ (650.56): C, 60.92; H, 5.58; N, 6.46; found: C, 61.04; H, 5.54; N, 6.40.

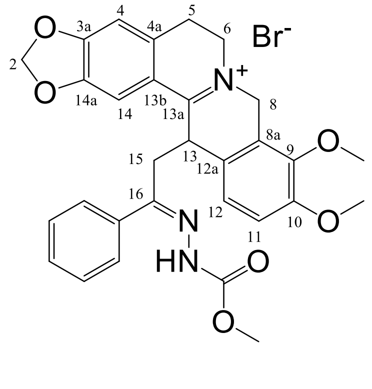

9,10-Dimethoxy-13-(2-(2-(methoxycarbonyl)hydrazono)-2-phenylethyl)-5,6,8,13-tetrahydro-[1,3]dioxolo[4,5-*g*]isoquinolino[3,2-*a*]isoquinolin-7-ium bromide (2b).

2b was isolated by precipitation in the reaction medium (DCM) in 42% yield (255 mg). Pale yellow amorphous solid mp: 164–165 °C with decomposition. ^{1}H NMR (400 MHz, DMSO$_{d6}$,

25 °C): 2.42–2.47 and 2.89–2.95 (2m, 2H, *C(5)H₂*), 3.35–3.45 (m, 2H, *C(15)H₂*), 3.68 (s, 3H, O*CH₃*), 3.78 (s, 3H, O*CH₃*), 3.83 (s, 3H, O*CH₃*), 3.91–4.00 and 4.04–4.10 (2m, 2H, C(6)*H₂*), 5.06 (d, 1H, *J* =19.6 Hz, *C(8)H₂*), 5.26 (t, 1H, *J* =7.2 Hz, *C(13)H*), 5.36 (d, 1H, *J* =19.6 Hz, C(8)*H₂*), 6.25 and 6.28 (2s, 2H, OC(2)*H₂*O), 7.03 (s, 1H, *C(14)H*), 7.17 (s, 1H, *C(12)H*), 7.18 (s, 1H, *C(11)H*), 7.32–7.37 (m, 3H, *ArH*), 7.47 (d, 2H, *J* =8.4 Hz, *ArH*), 7.61 (s, 1H, *C(4)H*), 10.16 (1s, 1H, *NH*); ^{13}C NMR (100 MHz, DMSO$_{d6}$, 25 °C): δ = 24.7 (t), 31.8 (t), 38.0 (d), 50.0 (q), 50.5 (t), 52.6 (t), 56.1 (q), 60.3 (q), 103.1 (t), 108.3 (d), 108.9 (d), 113.6 (d), 118.7 (s), 122.6 (d), 123.1 (s), 123.6 (s), 126.3 (d), 128.2 (d), 129.1 (d), 136.5 (s), 136.9 (s), 143.6 (s), 147.1 (s), 147.2 (s), 151.3 (s), 154.3 (s), 154.4 (s), 172.1 (s); IR (nujol): ν_{max} = 3409, 4249, 3168, 1741, 1720, 1641 cm^{-1}; HRMS (ESI) calcd. for $C_{30}H_{30}N_3O_6$ [M – Br]$^+$: 528.2135; found: 528.2172.

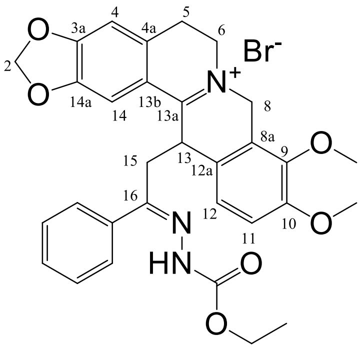

13-(2-(2-(Ethoxycarbonyl)hydrazono)-2-phenylethyl)-9,10-dimethoxy-5,6,8,13-tetrahydro-[1,3]dioxolo[4,5-*g*]isoquinolino[3,2-*a*]isoquinolin-7-ium bromide (2c).

2c was isolated by precipitation in the reaction medium (DCM) in 42% yield (260 mg). Pale yellow amorphous solid; mp: 159–161 °C with decomposition. ^{1}H NMR (400 MHz, DMSO$_{d6}$, 25 °C): 1.24 (t, 3H, *J* =6.8 Hz *CH₃*), 2.52–2.56 and 2.90–2.97 (2m, 2H, *C(5)H₂*), 3.39–3.45 (m, 2H, *C(15)H₂*), 3.78 (s, 3H, O*CH₃*), 3.83 (s, 3H, O*CH₃*), 3.92–4.01 and 4.05–4.08 (2m, 2H, C(6)*H₂*), 4.12 (q, 2H, *J* =7.2 Hz, *OCH₂*), 5.06 (d, 1H, *J* =19.6 Hz, *C(8)H₂*), 5.26 (t, 1H, *J* =7.2 Hz, *C(13)H*), 5.35 (d, 1H, *J* =19.6 Hz, C(8)*H₂*), 6.25 and 6.28 (2s, 2H, OC(2)*H₂*O), 7.03 (s, 1H, *C(14)H*), 7.17 (s, 1H, *C(12)H*), 7.18 (s, 1H, *C(11)H*), 7.30–7.39 (m, 3H, *ArH*), 7.48 (d, 2H, *J* =8.4 Hz, *ArH*), 7.61 (s, 1H, *C(4)H*) 10.08 (s, 1H, *NH*); ^{13}C NMR (100 MHz, DMSO$_{d6}$, 25 °C): δ = 14.5 (q), 24.7 (t), 31.8 (t), 38.0 (d), 50.5 (t), 52.6 (t), 56.0 (q), 60.3 (q), 60.7 (t), 103.1 (t), 108.3 (d), 109.0 (d), 113.6 (d), 118.6 (s), 122.6 (d), 123.1 (s), 123.6 (s), 126.3 (d), 128.2 (d), 129.0 (d), 136.5 (s), 136.6 (s), 143.6 (s), 146.9 (s), 147.1 (s), 151.3 (s),

153.8 (s), 154.3 (s), 172.1 (s); IR (nujol): ν_{max} = 3362, 3222, 1754, 1716, 1702, 1646 cm^{-1}; MS *m/z* (ESI): 542 $[M - Br]^+$; anal. calcd. for $C_{31}H_{32}BrN_3O_6$ (622.51): C, 59.81; H, 5.18; N, 6.75; found: C, 59.95; H, 5.13; N, 6.69.

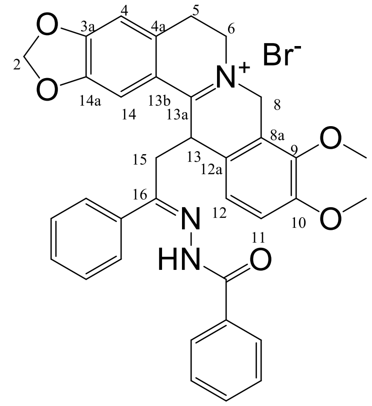

13-(2-(2-Benzoylhydrazono)-2-phenylethyl)-9,10-dimethoxy-5,6,8,13-tetrahydro-[1,3]dioxolo[4,5-*g*]isoquinolino[3,2-*a*]isoquinolin-7-ium bromide (2d).

2d was isolated by precipitation in the reaction medium (DCM) in 62% yield (405 mg). Pale yellow amorphous solid; mp: 174–176 °C with decomposition. ^{1}H NMR (400 MHz, DMSO$_{d6}$, 25 °C): 2.33–2.46 and 2.89–3.09 (2m, 2H, *C(5)H$_2$*), 3.59–4.22 (m, 10H, *C(15)H$_2$*, O*CH$_3$*, O*CH$_3$* and C(6)*H$_2$*), 5.07–5.53 (m, 3H, *C(8)H$_2$* and *C(13)H*), 6.25 and 6.26 (2s, 2H, OC(2)*H$_2$*O), 6.99–7.87 (m, 14H, *C(14)H*, *C(12)H*, *C(11)H*, *C(4)H*, *ArH*), 10.38, 10.48 and 10.99 (3brs, 1H, *NH* or O*H* enol form); ^{13}C NMR (100 MHz, DMSO$_{d6}$, 25 °C): δ = 24.8 (t), 31.9 (t), 38.2 (d), 50.5 (t), 52.7 (t), 55.9 (q), 60.2 (q), 103.2 (t), 108.6 (d), 108.8 (d), 113.7 (d), 118.7 (s), 122.6 (d), 123.1 (s), 123.6 (s), 126.6 (d), 126.9 (d), 128.3 (d), 128.9 (d), 129.5 (d), 131.7 (d), 133.2 (s), 136.6 (s), 136.9 (s), 143.6 (s), 147.4 (s), 150.8 (s), 151.3 (s), 154.4 (s), 163.8 (s), 172.1 (s); IR (nujol): ν_{max} = 3346, 3071, 3041, 1641 cm^{-1}; MS *m/z* (ESI): 574 $[M - Br]^+$; anal. calcd. for $C_{35}H_{32}BrN_3O_5$ (654.55): C, 64.22; H, 4.93; N, 6.42; found: C, 64.31; H, 4.87; N, 6.47.

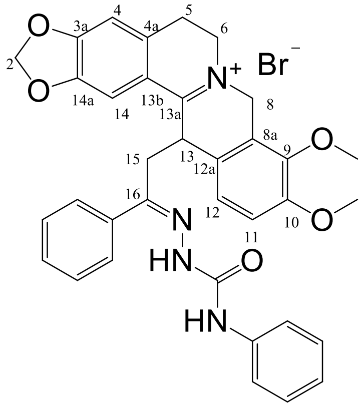

9,10-Dimethoxy-13-(2-phenyl-2-(2-(phenylcarbamoyl)hydrazono)ethyl)-5,6,8,13-tetrahydro-[1,3]dioxolo[4,5-*g*]isoquinolino[3,2-*a*]isoquinolin-7-ium bromide (2e).

2e was isolated by precipitation in the reaction medium (DCM) in 50% yield (335 mg). Pale yellow amorphous solid; mp: 173–175 °C with

decomposition. ^{1}H NMR (400 MHz, DMSO$_{d6}$, 25 °C): 2.51–2.55 and 2.89–2.92 (2m, 2H, *C(5)H$_2$*), 3.39–3.49 (m, 2H, *C(15)H$_2$*), 3.79 (s, 3H, O*CH$_3$*), 3.82 (s, 3H, O*CH$_3$*), 3.91–4.00 and 4.07–4.12 (2m, 2H, C(6)*H$_2$*), 5.08 (d, 1H, *J* =20.0, Hz *C(8)H$_2$*), 5.28 (t, 1H, *J* =7.6 Hz, *C(13)H*), 5.48 (d, 1H, *J* =19.2 Hz, C(8)*H$_2$*), 6.17 and 6.22 (2s, 2H, OC(2)*H$_2$*O), 6.96 (s, 1H, *C(14)H*), 7.03–7.07 (m, 1H, *ArH*), 7.19 (d, 1H, *J* =8.8Hz, *C(18)H*), 7.26–7.41 (m, 6H, *C(12)H* and *ArH*), 7.55–7.56 (m, 2H, *ArH*), 7.57 (s, 1H, *C(4)H*), 7.67–7.69 (m, 2H, *ArH*), 8.59 (s, 1H, *NH*), 10.02 (s, 1H, *NH*); ^{13}C NMR (100 MHz, DMSO$_{d6}$, 25 °C): δ = 24.7 (t), 32.1 (t), 38.0 (d), 50.5 (t), 52.7 (t), 56.0 (q), 60.3 (q), 103.0 (t), 108.2 (d), 109.1 (d), 113.7 (d), 118.8 (s), 120.1 (d), 122.7 (d), 123.2 (s), 123.5 (s), 126.6 (d), 128.3 (d), 128.5 (d), 129.0 (d), 136.6 (s), 136.8 (s), 138.7 (s), 143.6 (s), 144.2 (s), 147.0 (s), 147.0 (s), 151.3 (s), 153.1 (s), 154.4 (s), 172.1 (s); IR (nujol): ν_{max} = 3236, 3185, 3130, 1730, 1709, 1653 cm^{-1}; HRMS (ESI) calcd. for $C_{35}H_{33}N_4O_5$ [M – Br]$^+$: 589.2451; found: 589.2485.

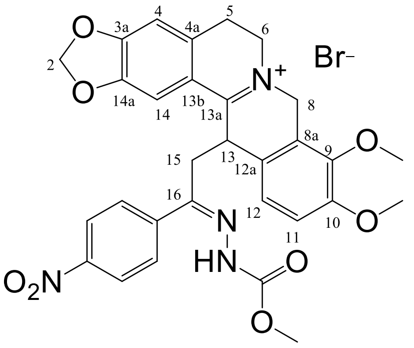

9,10-Dimethoxy-13-(2-(2-(methoxycarbonyl)hydrazono)-2-(4-nitrophenyl)ethyl)-5,6,8,13-tetrahydro-[1,3]dioxolo[4,5-*g*]isoquinolino[3,2-*a*]isoquinolin-7-ium bromide (2f).

2f was isolated by precipitation in the reaction medium (DCM) in 56% yield (365 mg). Pale yellow amorphous solid; mp: 160–161 °C with decomposition. ^{1}H NMR (400 MHz, DMSO$_{d6}$, 25 °C):, 2.65–2.75 and 2.98–3.04 (2m, 2H, *C(5)H$_2$*), 3.37–3.50 (1m, 2H, *C(15)H$_2$*), 3.72 (s, 3H, COO*CH$_3$*), 3.78 (s, 3H, O*CH$_3$*), 3.83 (s, 3H, O*CH$_3$*), 3.94–4.03 and 4.16–4.22 (2m, 2H, C(6)*H$_2$*), 5.08 (d, 1H, *J* =20.0 Hz, *C(8)H$_2$*), 5.24 (t, 1H, *J* =7.2 Hz, *C(13)H*), 5.42 (d, 1H, *J* =19.6 Hz, C(8)*H$_2$*), 6.25 and 6.27 (2s, 2H, OC(2)*H$_2$*O), 7.07 (s, 1H, *C(14)H*), 7.15 (s, 2H, *C(12)H* and *C(11)H*), 7.58 (1s, 1H, *C(4)H*), 7.74 (d, 2H, *J* =8.8 Hz, *ArH*), 8.18 (d, 2H, *J* =9.2 Hz, *ArH*), 10.45 (1s, 1H, *NH*); ^{13}C NMR (100 MHz, DMSO$_{d6}$, 25 °C): δ = 24.9 (t), 31.7 (t), 37.8 (d), 50.5 (t), 52.2 (t), 52.6 (q), 56.0 (q), 60.1 (q), 103.1 (t), 108.3 (d), 109.0 (d), 113.7 (d), 118.6 (s), 122.5 (d), 123.1 (s), 123.3 (s), 127.4 (s), 136.4 (s), 143.0 (d), 143.6 (s), 144.9

(s), 147.1 (s), 147.2 (d), 151.3 (s), 154.1 (s), 154.4 (s), 172.0 (s); IR (nujol): ν_{max} = 3079, 3058, 1752, 1734, 1720, 1707, 1644 cm^{-1}; MS *m/z* (ESI): 573 [M − Br]$^+$; anal. calcd. for $C_{30}H_{29}BrN_4O_8$ (653.48): C, 55.14; H, 4.47; N, 8.57; found: C, 55.28; H, 4.42; N, 8.48.

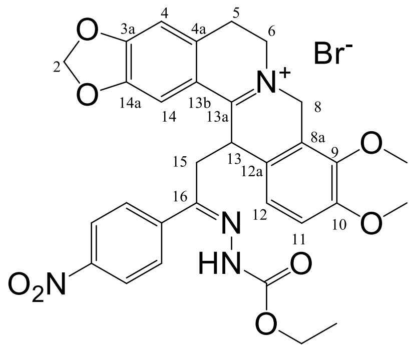

9,10-Dimethoxy-13-(2-(2-(methoxycarbonyl)hydrazono)-2-(4-nitrophenyl)ethyl)-5,6,8,13-tetrahydro-[1,3]dioxolo[4,5-*g*]isoquinolino[3,2-*a*]isoquinolin-7-ium bromide (2g).

2g was isolated by precipitation in the reaction medium (DCM) in 65% yield (382 mg). Pale yellow amorphous solid; mp: 161−162 °C with decomposition. ^{1}H NMR (400 MHz, DMSO$_{d6}$, 25 °C):, 1.26 (t, 3H, *J* =7.2 Hz, *CH$_3$*), 2.71–2.81 and 3.00–3.05 (2m, 2H, *C(5)H$_2$*), 3.38–3.48 (m, 2H, *C(15)H$_2$*), 3.77 (s, 3H, O*CH$_3$*), 3.82 (s, 3H, O*CH$_3$*), 3.95–4.04 (m, 1H, C(6)*H$_2$*), 4.17 (q, 2H, *J* =6.8 Hz, *OCH$_2$*), 4.19–4.22 (m, 1H, C(6)*H$_2$*), 5.08 (d, 1H, *J* =19.6 Hz, *C(8)H$_2$*), 5.24 (t, 1H, *J* =6.8 Hz, *C(13)H*), 5.44 (d, 1H, *J* =20.0 Hz, C(8)*H$_2$*), 6.25 and 6.27 (2s, 2H, OC(2)*H$_2$*O), 7.07 (s, 1H, *C(14)H*), 7.15 (s, 2H, *C(12)H* and *C(11)H*), 7.59 (s, 1H, *C(4)H*), 7.76 (d, 2H, *J* =8.8 Hz, *ArH*), 8.18 (d, 2H, *J*=9.2 Hz, *ArH*), 10.40 (1s, 1H, *NH*); ^{13}C NMR (100 MHz, DMSO$_{d6}$, 25 °C): δ = 14.4 (q), 24.9 (t), 31.8 (t), 37.9 (d), 50.6 (t), 52.6 (t), 56.0 (q), 60.1 (q), 61.0 (t), 103.1 (t), 108.3 (d), 109.0 (d), 113.7 (d), 118.6 (s), 122.5 (d), 123.1 (s), 123.2 (d), 123.3 (s), 127.5 (d), 136.4 (s), 143.0 (d), 143.6 (s), 144.7 (s), 147.1 (s), 147.1 (s), 151.3 (s), 153.6 (s), 154.4 (s), 171.9 (s); IR (nujol): ν_{max} = 3376, 3223, 1755, 1751, 1653 cm^{-1}; MS *m/z* (ESI): 587 [M − Br]$^+$; anal. calcd. for $C_{31}H_{31}BrN_4O_8$ (667.50): C, 55.78; H, 4.68; N, 8.39; found: C, 55.71; H, 4.75; N, 8.42.

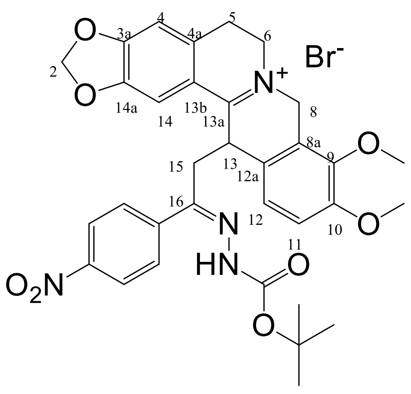

13-(2-(2-(*tert*-Butoxycarbonyl)hydrazono)-2-(4-nitrophenyl)ethyl)-9,10-dimethoxy-5,6,8,13-tetrahydro-

[1,3]dioxolo[4,5-*g*]isoquinolino[3,2-*a*]isoquinolin-7-ium bromide (2h).

2h was isolated by precipitation in the reaction medium (DCM) in 30% yield (208 mg). Pale yellow amorphous solid; mp: 180−181 °C with decomposition. ^{1}H NMR (400 MHz, DMSO$_{d6}$, 25 °C): 1.46 (s, 9H, C(*CH$_3$)$_3$*), 2.72−2.82 and 3.02−3.07 (2m, 2H, *C(5)H$_2$*), 3.41−3.50 (m, 2H, *C(15)H$_2$*), 3.76 (s, 3H, O*CH$_3$*), 3.82(s, 3H, O*CH$_3$*), 3.94−4.03 and 4.17−4.22 (2m, 2H, C(6)*H$_2$*), 5.07 (d, 1H, *J* =19.6 Hz, *C(8)H$_2$*), 5.24 (t, 1H, *J* =6.0 Hz, *C(13)H*), 5.40 (d, 1H, *J* =20.0 Hz, C(8)*H$_2$*), 6.28 (s, 2H, OC(2)*H$_2$*O), 7.09 (s, 1H, *C(14)H*), 7.15 (s, 2H, *C(12)H* and *C(11)H*), 7.58 (s, 1H, *C(4)H*), 7.75 (d, 2H, *J* =9.2 Hz, *ArH*), 8.18 (d, 2H, *J* =8.8 Hz, *ArH*), 10.13 (s, 1H, *NH*); ^{13}C NMR (100 MHz, DMSO$_{d6}$, 25 °C): δ = 24.9 (t), 27.9 (q), 31.9 (t), 38.0 (d), 50.6 (t), 52.7 (t), 56.0 (q), 60.2 (q), 80.3 (s), 103.2 (t), 108.5 (d), 109.1 (d), 113.6 (d), 118.7 (s), 122.5 (d), 122.9 (d), 123.2 (s), 123.3 (s), 127.4 (s), 136.4 (s), 143.3 (d), 143.5 (s), 147.1 (s), 147.1 (s), 150.4 (s), 151.3 (s), 152.4 (s), 154.4 (s), 172.0 (s); IR (nujol): ν_{max} = 3166, 3069, 1745, 1709, 1673, 1649 cm^{-1}; MS *m/z* (ESI): 615 [M − Br]$^{+}$; anal. calcd. for $C_{33}H_{35}BrN_4O_8$ (695.56): C, 56.98; H, 5.07; N, 8.05; found: C, 56.89; H, 5.02; N, 8.10.

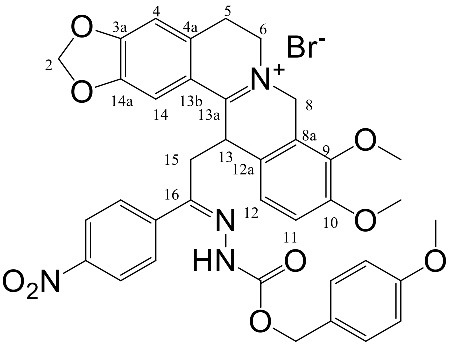

9,10-Dimethoxy-13-(2-(2-(((4-methoxybenzyl)oxy)carbonyl)hydrazono)-2-(4-nitrophenyl)ethyl)-5,6,8,13-tetrahydro-[1,3]dioxolo[4,5-*g*]isoquinolino[3,2-*a*]isoquinolin-7-ium bromide (2i).

2i was isolated by precipitation in the reaction medium (DCM) in 83% yield (630 mg). Pale yellow amorphous solid; mp: 168−169 °C with decomposition. ^{1}H NMR (400 MHz, DMSO$_{d6}$, 25 °C): 2.65−2.75 and 2.91−2.96 (2m, 2H, *C(5)H$_2$*), 3.45−3.51 (m, 2H, *C(15)H$_2$*), 3.74 (s, 3H, O*CH$_3$*), 3.78 (s, 3H, O*CH$_3$*), 3.83 (s, 3H, O*CH$_3$*), 3.91−4.00 and 4.12−4.17 (2m, 2H, C(6)*H$_2$*), 5.06 (d, 1H, *J* =20.0 Hz, *C(8)H$_2$*), 5.11 (s, 2H, O*CH$_2$*), 5.21 (t, 1H, *J* =6.4 Hz, *C(13)H*), 5.40 (d, 1H, *J* =19.6 Hz, C(8)*H$_2$*), 6.22 and 6.24 (2s, 2H, OC(2)*H$_2$*O), 6.95 (s, 1H,

C(14)H), 7.14 (s, 2H, *C(12)H* and *C(11)H*), 6.99 (d, 2H, *J* =8.8 Hz, *ArH*), 7.37 (d, 2H, *J* =8.8 Hz, *ArH*), 7.54 (s, 1H, *C(4)H*), 7.75 (d, 2H, *J* =8.8 Hz, *ArH*), 8.19 (d, 2H, *J* =9.2 Hz, *ArH*), 10.56 (s, 1H, *NH*); ^{13}C NMR (100 MHz, DMSO$_{d6}$, 25 °C): δ = 24.9 (t), 32.0 (t), 37.9 (d), 50.5 (t), 52.7 (t), 55.2 (q), 56.1 (q), 60.2 (q), 66.4 (t), 103.2 (t), 108.3 (d), 109.1 (d), 113.6 (s), 113.9 (d), 118.7 (s), 122.6 (d), 123.2 (s), 123.4 (d), 123.5 (s), 127.6 (d), 128.0 (s), 130.3 (d), 136.4 (s), 143.1 (d), 143.5 (s), 147.1 (s), 147.2 (s), 150.4 (s), 151.4 (s), 153.6 (s), 154.4 (s), 159.3 (s), 172.0 (s); IR (nujol): ν_{max} = 3448, 3338, 3219, 1758, 1645 cm^{-1}; MS *m/z* (ESI): 779 [M − Br]$^+$; anal. calcd. for $C_{37}H_{35}BrN_4O_9$ (759.60): C, 58.50; H, 4.64; N, 7.38; found: C, 58.36; H, 4.67; N, 7.48.

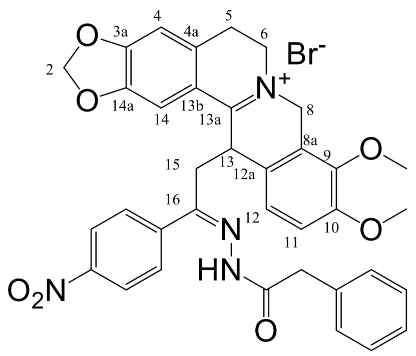

9,10-Dimethoxy-13-(2-(4-nitrophenyl)-2-(2-(2-phenylacetyl)hydrazono)ethyl)-5,6,8,13-tetrahydro-[1,3]dioxolo[4,5-*g*]isoquinolino[3,2-*a*]isoquinolin-7-ium bromide (2j).

2j was isolated by precipitation in the reaction medium (DCM) in 63% yield (449 mg). Pale yellow amorphous solid; mp: 158–160 °C with decomposition. ^{1}H NMR (400 MHz, DMSO$_{d6}$, 25 °C): 2.67–2.80 and 2.94–3.02 (2m, 2H, *C(5)H$_2$*), 3.35–3.44 and 3.50–3.56 (2m, 2H, *C(15)H$_2$*), 3.73–3.88 (m, 2H, *C(31)H$_2$*), 3.79 (s, 3H, O*CH$_3$*), 3.83 (s, 3H, O*CH$_3$*), 3.97–4.10 and 4.21–4.25 (2m, 2H, C(6)*H$_2$*), 5.09 (d, 1H, *J* =19.6 Hz, *C(8)H$_2$*), 5.21–5.30 (m, 1H, *C(13)H*), 5.57 (d, 1H, *J* =19.6 Hz, C(8)*H$_2$*), 6.27 and 6.32 (2s, 2H, OC(2)*H$_2$*O), 7.05 (s, 1H, *C(14)H*), 7.13–7.34 (m, 7H, *C(12)H*, *C(11)H* and *ArH*), 7.54 (s, 1H, *C(4)H*), 7.86 (d, 2H, *J* =8.8 Hz, *ArH*), 8.24 (d, 2H, *J* =8.4 Hz, *ArH*), 10.96 (1s, 1H, *NH*); ^{13}C NMR (100 MHz, DMSO$_{d6}$, 25 °C): δ = 24.9 (t), 30.7 (t), 32.1 (t), 38.0 (d), 50.6 (t), 52.8 (t), 56.1 (q), 60.3 (q), 103.2 (d), 108.3 (d), 109.3 (d), 113.6 (d), 118.7 (s), 122.7 (d), 123.3 (s), 123.6 (s), 126.5 (d), 127.7 (d), 128.2 (d), 129.4 (d), 135.1 (d), 136.5 (s), 142.8 (s), 143.2 (s), 143.5 (s), 147.0 (s), 147.3 (s), 151.4 (s), 154.3 (s), 154.4 (s), 171.8 (s), 173.5 (s); IR (nujol): ν_{max} = 3236, 3122,

3071, 1755, 1713, 1695, 1686, 1649 cm^{-1}; HRMS (ESI) calcd. for $C_{36}H_{33}N_4O_7$ [M − Br]$^+$: 633.2349; found: 633.2368.

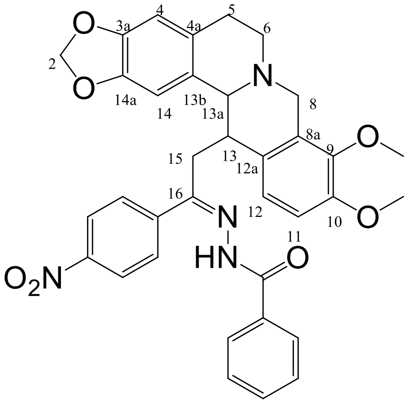

N'-(2-(9,10-Dimethoxy-6,8,13,13a-tetrahydro-5*H*-[1,3]dioxolo[4,5-*g*]isoquinolino[3,2-*a*]isoquinolin-13-yl)-1-(4-nitrophenyl)ethylidene)benzohydrazide (2k).

2k was isolated by precipitation in the reaction medium (DCM) in 62% yield (433 mg). Pale yellow amorphous solid; mp: 172−173 °C with decomposition. ^{1}H NMR (400 MHz, DMSO$_{d6}$, 25 °C): 2.67−2.70 and 2.98−3.04 (2m, 2H, *C(5)H2*), 3.59−3.82 (1m, 8H, *C(15)H$_2$*, O*CH$_3$* and O*CH$_3$*), 3.96−4.05 and 4.15−4.25 (2m, 2H, C(6)*H$_2$*), 5.08 (d, 1H, *J* =20.0 Hz, *C(8)H$_2$*), 5.40 (brs, 2H, *C(13)H* and C(8)*H$_2$*), 6.26 and 6.28 (2s, 2H, OC(2)*H$_2$*O), 7.03 (s, 1H, *C(14)H*), 7.10−7.13 (m, 2H, *ArH*), 7.45−7.56 (m, 3H, *C(12)H* and *ArH*), 7.60−7.64 (m, 1H, *ArH*), 7.76−7.80 (m, 2H, *ArH*), 8.05−8.07 (m, 1H, *C(11)H*), 8.14−8.16 (m, 2H, *ArH*), 8.28−8.31 (m, 1H, *C(4)H*), 10.28 and 10.83 (s and brs, 1H, *NH*); ^{13}C NMR (100 MHz, DMSO$_{d6}$, 25 °C): δ = 25.0 (t), 31.8 (t), 38.0 (d), 50.6 (t), 52.7 (t), 56.0 (q), 60.1 (q), 103.2 (t), 108.6 (d), 108.8 (d), 113.7 (d), 118.6 (s), 122.5(d), 123.0 (d), 123.3 (s), 123.6 (s), 127.1 (d), 127.7 (d), 128.1 (d), 128.4 (d), 131.7 (s), 133.2 (s), 136.5 (s), 143.1 (s), 143.6 (s), 147.3 (s), 147.4 (s), 151.3 (s), 154.5 (s), 166.6 (s), 171.9 (s); IR (nujol): ν_{max} = 3256, 3194, 3129, 1751, 1718, 1700, 1649 cm^{-1}; HRMS (ESI) calcd. for $C_{35}H_{31}N_4O_7$ [M − Br]$^+$: 619.2193; found: 619.2227.

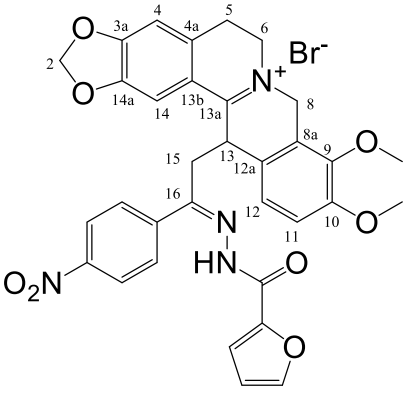

13-(2-(2-(Furan-2-carbonyl)hydrazono)-2-(4-nitrophenyl)ethyl)-9,10-dimethoxy-5,6,8,13-tetrahydro-[1,3]dioxolo[4,5-*g*]isoquinolino[3,2-*a*]isoquinolin-7-ium bromide (2l).

2l was isolated by precipitation in the reaction medium (DCM) in 56% yield (385 mg). Pale yellow amorphous solid; mp: 170–171 °C with decomposition. ^{1}H NMR (400 MHz, DMSO$_{d6}$, 25 °C): 2.66–2.75 and 2.96–3.02 (2m, 2H, *C(5)H$_2$*), 3.56–3.66 (m, 2H, *C(15)H$_2$*), 3.73 (s, 3H, O*CH$_3$*), 3.75 (s, 3H, O*CH$_3$*), 3.94–4.03 and 4.16–4.20 (2m, 2H, C(6)*H$_2$*), 5.08 (d, 1H, *J* =20.0 Hz, *C(8)H$_2$*), 5.35–5.45 (m, 2H, *C(13)H* and *C(8)H$_2$*), 6.23 and 6.25 (brs and s, 2H, OC(2)*H$_2$*O), 6.73 (dd, 1H, *J* =3.6 Hz, *J* =1.6 Hz, *furan*), 6.97 (s, 1H, *C(14)H*), 7.10 (d, 1H, *J* =8.4 Hz, *C(12)H*), 7.15 (d, 1H, *J* =8.4 Hz, *C(11)H*), 7.24 (brs, 1H, *furan*), 7.75 (brs, 1H, *C(4)H*), 7.81 (d, 2H, *J* =8.8 Hz, *ArH*), 7.99 (dd, 1H, *J* =1.2 Hz, *J* =0.4 Hz, *furan*), 8.12–8.26 (m, 2H, *ArH*), 10.75 (brs, 1H, *NH*); ^{13}C NMR (100 MHz, DMSO$_{d6}$, 25 °C): δ = 25.0 (t), 32.0 (t), 37.9 (d), 50.6 (t), 52.7 (t), 56.0 (q), 60.1 (q), 103.2 (t), 108.4 (d), 108.8 (d), 112.0 (d), 113.8 (d), 118.5 (s), 122.4 (d), 123.0 (s), 123.0 (d), 123.2 (d), 123.3 (s), 123.4 (d), 127.8 (d), 133.9 (s), 136.5 (s), 142.9 (s), 143.6 (s), 145.6 (s), 146.4 (s), 147.4 (s), 151.3 (s), 154.5 (s), 165.7 (s), 171.8 (s); IR (nujol): ν_{max} = 3351, 3160, 1700, 1653 cm^{-1}; HRMS (ESI) calcd. for $C_{33}H_{29}N_4O_8$ [M – Br]$^+$: 609.1985; found: 619.2028.

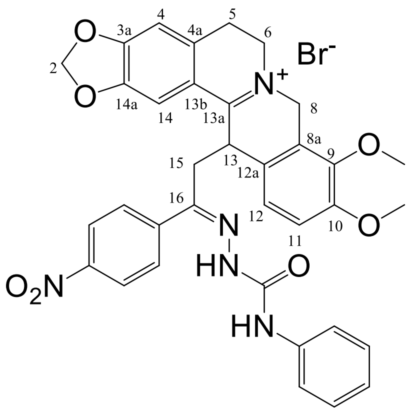

9,10-Dimethoxy-13-(2-(4-nitrophenyl)-2-(2-(phenylcarbamoyl)hydrazono)ethyl)-5,6,8,13-tetrahydro-[1,3]dioxolo[4,5-*g*]isoquinolino[3,2-*a*]isoquinolin-7-ium bromide (2m).

2m was isolated by precipitation in the reaction medium (DCM) in 60% yield (428 mg). Pale yellow amorphous solid; mp: 176–178 °C with decomposition. ^{1}H NMR (400 MHz, DMSO$_{d6}$, 25 °C): 2.73–2.83 and 2.96–3.01 (2m, 2H, *C(5)H$_2$*), 3.40–3.46 and 3.51–3.57 (2m, 2H, *C(15)H$_2$*), 3.78 (s, 3H, O*CH$_3$*), 3.82 (s, 3H, O*CH$_3$*), 3.93–4.02 and 4.18–4.23 (2m, 2H, C(6)*H$_2$*), 5.10 (d, 1H, *J* =20.0 Hz, *C(8)H$_2$*), 5.27 (t, 1H, *J* =7.2 Hz, *C(13)H*), 5.59 (d, 1H, *J* =19.6 Hz, C(8)*H$_2$*), 6.21 and 6.24 (2s, 2H, OC(2)*H$_2$*O), 7.00 (s, 1H, *C(14)H*), 7.08 (m, 1H, *ArH*), 7.18 (d, 1H, *J* =8.4 Hz, *C(12)H*), 7.25 (d, 1H, *J* =8.8 Hz, *C(11)H*),

7.35 (m, 2H, *ArH*), 7.54 (m, 3H, *C(4)H* and *ArH*), 8.00 (d, 2H, *J* =8.8 Hz, *ArH*), 8.19 (d, 2H, *J* =8.8, *ArH*), 8.76 (s, 1H, *NH*), 10.30 (s, 1H, *NH*); ^{13}C NMR (100 MHz, DMSO$_{d6}$, 25 °C): δ = 24.9 (t), 32.3 (t), 38.0 (d), 50.6 (t), 52.8 (t), 56.1 (q), 60.3 (q), 103.1 (t), 108.3 (d), 109.2 (d), 113.7 (d), 118.8 (s), 120.7 (d), 122.7 (d), 123.1 (s), 123.2 (d), 123.3 (s), 123.5 (s), 127.9 (d), 128.5 (d), 136.5 (s), 138.4 (s), 142.0 (d), 142.7 (s), 143.6 (s), 147.0 (s), 147.1 (s), 151.4 (s), 152.9 (s), 154.4 (s), 171.9 (s); IR (nujol): ν_{max} = 3243, 3164, 1733, 1697, 1683, 1649 cm^{-1}; HRMS (ESI) calcd. for $C_{35}H_{32}N_5O_7$ [M – Br]$^+$: 634.2302; found: 634.2322.

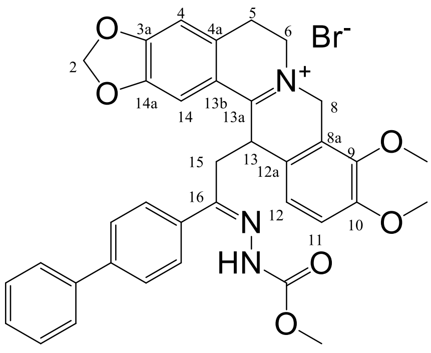

13-(2-([1,1'-Biphenyl]-4-yl)-2-(2-(methoxycarbonyl)hydrazono)ethyl)-9,10-dimethoxy-5,6,8,13-tetrahydro-[1,3]dioxolo[4,5-*g*]isoquinolino[3,2-*a*]isoquinolin-7-ium bromide (2n).

2n was isolated by precipitation in the reaction medium (DCM) in 30% yield (206 mg). Pale yellow amorphous solid; mp: 162−164 °C with decomposition. ^{1}H NMR (400 MHz, DMSO$_{d6}$, 25 °C): 2.40–2.46 and 2.90–2.94 (2m, 2H, *C(5)H$_2$*), 3.38–3.47 (m, 2H, *C(15)H$_2$*), 3.70 (s, 3H, O*CH$_3$*), 3.77 (s, 3H, O*CH$_3$*), 3.83 (s, 3H, O*CH$_3$*), 3.93–3.99 and 4.05–4.12 (2m, 2H, C(6)*H$_2$*), 5.06 (d, 1H, *J* =19.6 Hz, *C(8)H$_2$*), 5.26 (t, 1H, *J* =7.2 Hz, *C(13)H*), 5.38 (d, 1H, *J* =19.6 Hz, C(8)*H$_2$*), 6.25 and 6.28 (2s, 2H, OC(2)*H$_2$*O), 7.04 (s, 1H, *C(14)H*), 7.19–7.21 (m, 2H, *C(12)H*, *C(11)H*), 7.39–7.42 (m, 1H, *ArH*), 7.49–7.70 (m, 9H, *ArH),* 10.16 (s, 1H, *NH*); ^{13}C NMR (100 MHz, DMSO$_{d6}$, 25 °C): δ = 28.7 (t), 29.2 (t), 39.8 (d), 49.9 (t), 52.0 (t), 55.5 (q), 59.3 (q), 63.4 (q), 100.7 (t), 106.2 (d), 108.1 (d), 110.3 (d), 125.2 (s), 126.2(d), 126.5 (d), 126.8 (s), 127.5 (s), 127.6 (s), 127.8 (s), 128.9 (s), 129.0 (d), 129.1 (s), 137.2 (s), 139.4 (s), 139.9 (s), 143.9 (s), 145.7 (s), 146.3 (s), 149.4 (s), 150.2 (s), 154.1 (s); IR (nujol): ν_{max} = 3164, 3054, 1774, 1751, 1717, 1697, 1685, 1670, 1648 cm^{-1}; MS *m/z* (ESI): 604 [M − Br]$^+$; anal. calcd. for $C_{36}H_{34}BrN_3O_6$ (684.58): C, 63.16; H, 5.01; N, 6.14; found: C, 63.25; H, 5.00; N, 6.06.

2.3. General Procedure for the Synthesis of Hydrazono-tetrahydroberberines (THBERs) 3a–n.

To a solution of hydrazono-DHBERs **2a–n** (0.2 mmol) in methanol (2.0 mL) at room temperature sodium borohydride (0.4 mmol) was added. The reaction was allowed to stand at room temperature under magnetic stirring until the complete disappearing of the staring hydrazono-DHBERs **2a–n** (2.0–3.0 h, TLC monitoring). The reaction solvent was then evaporated under reduced pressure. The crude mixtures were then purified by column chromatography on silica gel (elution with cyclohexane-ethyl acetate mixtures) to afford products **3a–n** that were crystallized in a mixture of diethyl ether-petroleum ether.

2.4. Characterization data of hydrazono-tetrahydroberberines (THBERs) 3a–n

***tert*-Butyl 2-(2-(9,10-dimethoxy-6,8,13,13a-tetrahydro-5*H*-[1,3]dioxolo[4,5-*g*]isoquinolino[3,2-*a*]isoquinolin-13-yl)-1-phenylethylidene)hydrazinecarboxylate (3a).**

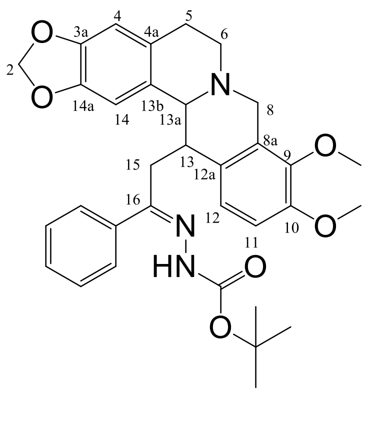

3a was isolated by chromatographic column on silica gel (ethyl acetate/cyclohexane, 30/70) in 95% yield (109 mg). White amorphous solid; mp: 162–164 °C. ^{1}H NMR (400 MHz, DMSO*d6*, 25 °C): 1.45 (s, 9H, C(*CH$_3$)$_3$*), 2.29–2.34 (m, 1H, *C(15)H$_2$*), 2.62–2.75 (m, 3H, *C(15)H$_2$, C(5)H2,* C(6)*H$_2$*), 3.04–3.12 (m, 1H, *C(5)H$_2$*), 3.23–3.26 (m, 1H, C(6)*H$_2$*), 3.55 (d, 1H, *J* =15.6 Hz, *C(8)H$_2$*), 3.67 (s, 3H, O*CH$_3$*), 3.70 (s, 3H, O*CH$_3$*), 3.84 (brs, 1H, *C(13a)H*), 3.94–3.98 (m, 1H, *C(13)H*), 4.15 (d, 1H, *J* =16.0 Hz, *C(8)H$_2$*), 5.99 and 6.00 (2s, 2H, OC(2)*H$_2$*O), 6.41 (d, 1H, *J* =8.4 Hz, *C(12)H*), 6.57 (d, 1H, *J* =8.8 Hz, *C(11)H*), 6.75 (s, 1H, *C(4)H*), 7.03 (s, 1H, *C(14)H*), 7.32–7.38 (m, 3H, *ArH*), 7.63–7.65 (m, 2H, *ArH*), 11.04 (brs, 1H, *NH*); ^{13}C NMR (100 MHz, DMSO*d6*, 25 °C): δ = 28.0 (q), 28.6 (t), 29.1 (t), 39.9 (d), 50.0 (t), 53.1 (t), 55.4 (q), 59.4 (q), 63.4 (d), 79.1 (s), 100.7 (t), 106.1 (d),

108.1 (d), 110.2 (d), 125.2 (d), 126.0 (d), 127.4 (d), 127.8 (s), 128.0 (d), 128.4 (s), 128.7 (s), 129.1 (s), 138.2 (s), 143.8 (s), 145.7 (s), 146.3 (s), 148.9 (s), 150.1 (s), 152.3 (s); IR (nujol): ν_{max} = 3288, 3129, 1742 cm^{-1}; HRMS (ESI) calcd. for $C_{33}H_{38}N_3O_6$ [M + H]$^+$: 572.2761; found: 572.2814.

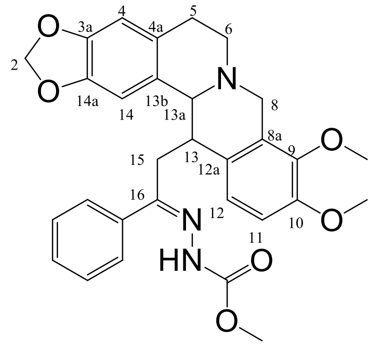

Methyl-2-(2-(9,10-dimethoxy-6,8,13,13a-tetrahydro-5*H*-[1,3]dioxolo[4,5-*g*]isoquinolino[3,2-*a*]isoquinolin-13-yl)-1-phenylethylidene)hydrazinecarboxylate (3b).

3b was isolated by chromatographic column on silica gel (ethyl acetate/cyclohexane, 40/60) in 87% yield (92 mg). White amorphous solid; mp: 165–167 °C. ^{1}H NMR (400 MHz, DMSO$_{d6}$, 25 °C): 2.38–2.43 (m, 1H, *C(15)H$_2$*), 2.60–2.72 (m, 3H, *C(15)H$_2$*, *C(5)H2*, C(6)*H$_2$*), 3.02–3.10 (m, 1H, C(6)*H$_2$*), 3.23–3.26 (m, 1H, *C(5)H$_2$*), 3.53 (d, 1H, *J* =16.0 Hz, *C(8)H$_2$*), 3.64 (s, 3H, *OCH$_3$*), 3.66 (s, 3H, O*CH$_3$*), 3.67 (s, 3H, O*CH$_3$*), 3.82 (brs, 1H, *C(13a)H*), 3.91–3.94 (m, 1H, *C(13)H*), 4.13 (d, 1H, *J* =16.0 Hz, *C(8)H$_2$*), 5.98 and 5.99 (2s, 2H, OC(2)*H$_2$*O), 6.42 (d, 1H, *J* =8.4 Hz, *C(12)H*), 6.58 (d, 1H, *J* =8.4 Hz, *C(11)H*), 6.73 (s, 1H, *C(4)H*), 7.01 (s, 1H, *C(14)H*), 7.33–7.39 (m, 3H, *ArH*), 7.61–7.63 (m, 2H, *ArH*), 11.13 (s, 1H, *NH*); ^{13}C NMR (100 MHz, DMSO$_{d6}$, 25 °C): δ = 28.6 (t), 29.2 (t), 39.8 (d), 49.9 (t), 52.0 (q), 53.2 (t), 55.5 (q), 59.3 (q), 63.3 (d), 100.7 (t), 106.2 (d), 108.1 (d), 110.2 (d), 125.1 (d), 126.2 (d), 127.5 (d), 127.7 (s), 128.0 (d), 128.5 (s), 128.9 (s), 129.1 (s), 138.0 (s), 143.9 (s), 145.4 (s), 145.7 (s), 146.2 (s), 150.2 (s), 154.1 (s); IR (nujol): ν_{max} = 3345, 1751 cm^{-1}; MS *m/z* (ESI): 530 [M + H$^+$]; anal. calcd. for $C_{30}H_{31}N_3O_6$ (529.58): C, 68.04; H, 5.90; N, 7.93; found: C, 68.14; H, 5.96; N, 7.85.

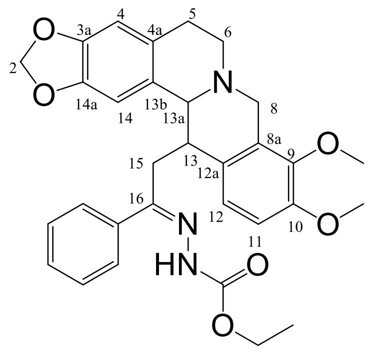

Ethyl 2-(2-(9,10-dimethoxy-6,8,13,13a-tetrahydro-5*H*-[1,3]dioxolo[4,5-*g*]isoquinolino[3,2-*a*]isoquinolin-13-yl)-1-phenylethylidene)hydrazinecarboxylate (3c).

3c was isolated by chromatographic column on silica gel (ethyl acetate/cyclohexane, 40/60) in 89% yield (97 mg). White amorphous solid; mp: 168−169 °C. ^{1}H NMR (400 MHz, DMSO*d6*, 25 °C): 1.24 (t, 3H, *J*=6.8 Hz, *CH3*), 2.35–2.40 (m, 1H, *C(15)H2*), 2.62–2.74 (m, 3H, *C(15)H2*, *C(5)H2*, C(6)*H2*), 3.04–3.12 (m, 1H, *C(5)H2*), 3.23–3.26 (m, 1H, C(6)*H2*), 3.54 (d, 1H, *J* =15.6 Hz, *C(8)H2*), 3.66 (s, 3H, O*CH3*), 3.68 (s, 3H, O*CH3*), 3.85 (brs, 1H, *C(13a)H*), 3.93–3.96 (m, 1H, *C(13)H*), 4.04–4.11 (m, 2H, O*CH2*), 4.14 (d, 1H, *J* =16.4 Hz, *C(8)H2*), 5.99 and 6.00 (2s, 2H, OC(2)*H2*O), 6.42 (d, 1H, *J* =8.8 Hz, *C(12)H*), 6.58 (d, 1H, *J* =8.4 Hz, *C(11)H*), 6.74 (s, 1H, *C(4)H*), 7.02 (s, 1H, *C(14)H*), 7.24–7.37 (2m, 3H, *ArH*), 7.62−7.64 (2m, 2H, *ArH*), 11.25 (brs, 1H, *NH*); ^{13}C NMR (100 MHz, DMSO*d6*, 25 °C): δ = 14.5 (q), 28.6 (t), 29.2 (t), 40.0 (d), 49.9 (t), 53.1 (t), 55.5 (q), 59.3 (q), 60.3 (t), 63.5 (d), 100.7 (t), 106.1 (d), 108.1 (d), 110.2 (d), 125.2 (d), 126.1 (d), 127.4 (d), 127.7 (s), 128.0 (d), 128.4 (s), 128.8 (s), 129.1(s), 138.1 (s), 143.9 (s), 145.7 (s), 146.2 (s), 149.8 (s), 150.1 (s), 153.4 (s); IR (nujol): ν_{max} = 3304, 1750, 1622 cm^{-1}; HRMS (ESI) calcd. for $C_{31}H_{34}N_3O_6$ [M + H]$^{+}$: 544.2448; found: 544.2416.

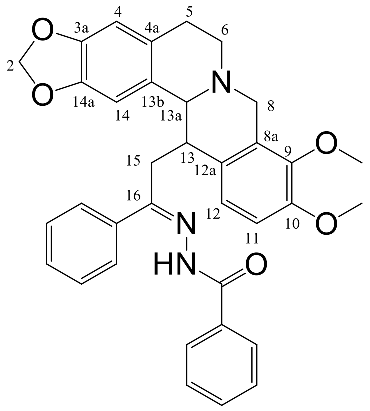

N'-(2-(9,10-Dimethoxy-6,8,13,13a-tetrahydro-5*H*-[1,3]dioxolo[4,5-*g*]isoquinolino[3,2-*a*]isoquinolin-13-yl)-1-phenylethylidene)benzohydrazide (3d).

3d was isolated by chromatographic column on silica gel (ethyl acetate/cyclohexane, 30/70) in 98% yield (112 mg). White amorphous solid; mp: 174−176 °C with decomposition. ^{1}H NMR (400 MHz, DMSO*d6*, 25 °C): 2.42–2.45 (m, 1H, *C(15)H2*), 2.55–2.67 (m, 3H, *C(15)H2*, *C(5)H2*, C(6)*H2*), 2.79–2.82 (m, 1H, *C(5)H2*), 2.94–3.00 (m, 1H, C(6)*H2*), 3.12–3.16 (m, 1H, *C(8)H2*), 3.50 (s, 3H, O*CH3*), 3.59 (brs, 1H, *C(13a)H*), 3.64 (s, 3H, O*CH3*), 3.70 (s, 1H, *C(8)H2*), 3.80 (m, 1H, *C(13)*, 5.99 and 6.00 (2s, 2H, OC(2)*H2*O), 6.45 (d, 1H, *J* =8.4 Hz, *C(12)H*), 6.66 (d, 1H, *J* =8.8 Hz, *C(11)H*), 6.74 (s, 1H, *C(4)H*), 7.02 (s, 1H, *C(14)H*), 7.29–7.71 (m, 10H, *2 ArH*), 10.40 (s, 1H, *NH*);

^{13}C NMR (100 MHz, $DMSO_{d6}$, 25 °C): δ = 28.7 (t), 29.6 (t), 39.9 (d), 50.5 (t), 53.7 (t), 55.5 (q), 59.3 (q), 63.3 (d), 100.7 (t), 106.1 (d), 108.2 (d), 110.6 (d), 124.8 (d), 126.1 (d), 126.5 (d), 127.0 (d), 127.4 (d), 127.8 (s), 128.3 (d), 128.6 (s), 129.2 (s), 129.5 (s), 131.7 (s), 134.1 (s), 137.8 (s), 144.1 (s), 145.7 (s), 146.2 (s), 150.3 (s), 154.2 (s), 163.1 (s); IR (nujol): ν_{max} = 3271, 1713, 1683 cm^{-1}; MS *m/z* (ESI): 576 [M + H^+]; anal. calcd. for $C_{35}H_{33}N_3O_5$ (575.65): C, 73.03; H, 5.78; N, 7.30; found: C, 72.89; H, 5.84; N, 7.36.

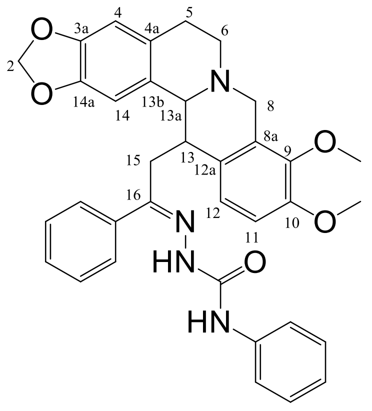

2-(2-(9,10-Dimethoxy-6,8,13,13a-tetrahydro-5*H*-[1,3]dioxolo[4,5-*g*]isoquinolino[3,2-*a*]isoquinolin-13-yl)-1-phenylethylidene)-*N*-phenylhydrazinecarboxyamide (3e).

3e was isolated by chromatographic column on silica gel (ethyl acetate/cyclohexane, 40/60) in 91% yield (108 mg). White amorphous solid; mp: 181–182 °C with decomposition. ^{1}H NMR (400 MHz, $DMSO_{d6}$, 25 °C): 2.43–2.46 (m, 1H, *C(15)H₂*), 2.61–2.76 (m, 3H, *C(15)H₂*, *C(5)H2*, C(6)*H₂*), 3.08–3.15 (m, 1H, *C(5)H₂*), 3.27–3.31 (m, 1H, C(6)*H₂*), 3.53 (d, 1H, *J* =16.0 Hz, *C(8)H₂*), 3.62 (s, 3H, O*CH₃*), 3.62 (s, 3H, O*CH₃*), 3.85 (brs, 1H, *C(13a)H*), 3.95–3.98 (m, 1H, *C(13)H*), 4.26 (d, 1H, *J* =16.0 Hz, *C(8)H₂*), 5.99–6.00 (m, 2H, OC(2)*H₂*O), 6.50 (d, 1H, *J* =8.8 Hz, *C(12)H*), 6.58 (d, 1H, *J* =8.4 Hz, *C(11)H*), 6.74 (s, 1H, *C(4)H*), 6.96–6.99 (m, 1H, *ArH*), 7.03 (s, 1H, *C(14)H*), 7.24 (t, 2H, *J* =7.2 Hz, *ArH*), 7.34–7.40 (m, 3H, *ArH*), 7.56 (d, 2H, *J* =7.6 Hz, *ArH*), 7.83–7.86 (m, 2H, *ArH*), 8.50 (s, 1H, *NH*), 10.79 (s, 1H, *NH*); ^{13}C NMR (100 MHz, $DMSO_{d6}$, 25 °C): δ = 28.7 (t), 29.5 (t), 40.2 (d), 49.7 (t), 53.1 (t), 55.5 (q), 59.3 (q), 63.1 (d), 100.7 (t), 106.2 (d), 108.1 (d), 110.2 (d), 119.5 (d), 122.4 (d), 125.0 (d), 126.5 (d), 127.8 (d), 127.8 (s), 128.0 (d), 128.4 (d), 128.4 (s), 129.1 (s), 129.2 (s), 137.8 (s), 138.8 (s), 144.0 (s), 145.7 (s), 146.2 (s), 147.0 (s), 150.2 (s), 153.2 (s); IR (nujol): ν_{max} = 3267, 3256, 1769, 1653 cm^{-1}; MS *m/z* (ESI): 591 [M + H^+]; anal. calcd. for $C_{35}H_{34}N_4O_5$ (590.67): C, 71.17; H, 5.80; N, 9.49; found: C, 71.03; H, 5.87; N, 9.60.

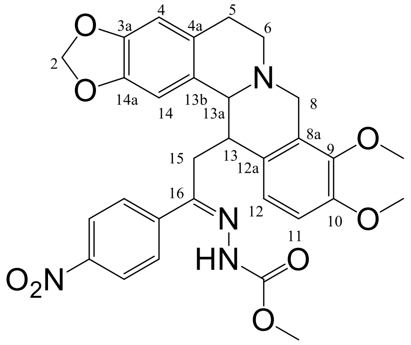

Methyl-2-(2-(9,10-dimethoxy-6,8,13,13a-tetrahydro-5*H*-[1,3]dioxolo[4,5-*g*]isoquinolino[3,2-*a*]isoquinolin-13-yl)-1-(4-nitrophenyl)ethylidene)hydrazinecarboxylate (3f).

3f was isolated by chromatographic column on silica gel (ethyl acetate/cyclohexane, 40/60) in 82% yield (94 mg). White amorphous solid; mp: 180−181 °C. ^{1}H NMR (400 MHz, DMSO$_{d6}$, 25 °C): 2.41−2.46 (m, 1H, *C(15)H$_2$*), 2.60−2.73 (m, 3H, *C(15)H$_2$, C(5)H2,* C(6)*H$_2$*), 2.79−2.85 (m, 1H, C(6)*H$_2$*), 3.04−3.11 (m, 1H, *C(5)H2*), 3.52 (d, 1H, *J* =15.6 Hz, *C(8)H$_2$*), 3.64 (s, 3H, *OCH$_3$*), 3.67 (s, 3H, O*CH$_3$*), 3.69 (s, 3H, O*CH$_3$*), 3.85 (brs, 1H, *C(13a)H*), 3.97−4.01 (m, 1H, *C(13)H*), 4.10 (d, 1H, *J* =16.0 Hz, *C(8)H$_2$*), 5.99 and 6.00 (2s, 2H, OC(2)*H$_2$*O), 6.42 (d, 1H, *J* =8.8 Hz, *C(12)H*), 6.59 (d, 1H, *J* =8.8 Hz, *C(11)H*), 6.72 (s, 1H, *C(4)H*), 6.98 (s, 1H, *C(14)H*), 7.85 (d, 2H, *J*=8.4 Hz, *ArH*), 8.17 (d, 2H, *J*=8.4 Hz, *ArH*), 11.58 (s, 1H, *NH*);^{13}C NMR (100 MHz, DMSO$_{d6}$, 25 °C): δ = 28.5 (t), 29.0 (t), 39.9 (d), 49.8 (t), 52.1 (q), 53.0 (t), 55.5 (q), 59.2 (q), 63.1 (d), 100.6 (t), 106.1 (d), 108.0 (d), 110.4 (d), 123.1 (d), 124.9 (d), 127.1 (d), 127.3 (s), 127.5 (s), 128.8 (s), 129.0 (s), 143.9 (s), 144.3 (s), 145.7 (s), 146.1 (s), 146.9 (s), 148.4 (s), 150.1 (s), 152,3 (s); IR (nujol): ν_{max} = 3258, 1733, 1691 cm^{-1}; MS *m/z* (ESI): 575 [M + H$^+$]; anal. calcd. for $C_{30}H_{30}N_4O_8$ (574.78): C, 62.71; H, 5.26; N, 9.75; found: C, 62.79 H, 5.29; N, 9.64.

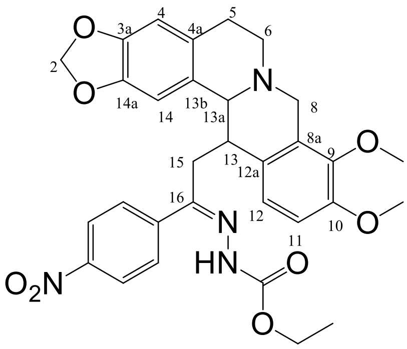

Ethyl-2-(2-(9,10-dimethoxy-6,8,13,13a-tetrahydro-5*H*-[1,3]dioxolo[4,5-*g*]isoquinolino[3,2-*a*]isoquinolin-13-yl)-1-(4-nitrophenyl)ethylidene)hydrazinecarboxylate (3g).

3g was isolated by chromatographic column on silica gel (ethyl acetate/cyclohexane, 40/60) in 95% yield (112 mg). White amorphous solid; mp: 178−179 °C; ^{1}H NMR (400 MHz, DMSO$_{d6}$, 25 °C): 1.26 (t, 3H, *J*=7.2

Hz, CH_3), 2.36–2.41 (m, 1H, *C(15)H₂*), 2.62–2.86 (m, 3H, *C(15)H₂, C(5)H₂,* C(6)*H₂*), 3.04–3.12 (m, 1H, *C(5)H₂*), 3.24–3.27 (m, 1H, C(6)*H₂*), 3.53 (d, 1H, *J* =16.0 Hz, *C(8)H₂*), 3.64 (s, 3H, O*CH₃*), 3.66 (s, 3H, O*CH₃*), 3.86 (brs, 1H, *C(13a)H*), 4.00–4.06 (m, 1H, *C(13)H*), 4.05–4.17 (m, 3H, *OCH₂* and *C(8)H₂*), 5.99 and 6.00 (2s, 2H, OC(2)*H₂*O), 6.41 (d, 1H, *J* =8.8 Hz, *C(12)H*), 6.58 (d, 1H, *J* =8.8 Hz, *C(11)H*), 6.74 (s, 1H, *C(4)H*), 7.00 (s, 1H, *C(14)H*), 7.87 (d, 2H, *J* =8.8 Hz, *ArH*), 8.18 (d, 2H, *J* =9.2 Hz, *ArH*), 11.77 (brs, 1H, *NH*); ^{13}C NMR (100 MHz, DMSO$_{d6}$, 25 °C): δ = 14.4 (q), 28.5 (t), 29.1 (t), 39.9 (d), 49.9 (t), 53.0 (t), 55.5 (q), 59.3 (q), 60.7 (t), 63.2 (d), 100.7 (t), 106.2 (d), 108.1 (d), 110.3 (d), 123.2 (d), 125.1 (d), 127.2 (d), 127.3 (s), 127.6 (s), 128.7 (s), 129.1 (s), 143.9 (s), 144.4 (s), 145.8 (s), 146.2 (s), 146.9 (s), 148.0 (s), 150.2 (s), 153.4 (s); IR (nujol): ν_{max} = 3358, 2976, 1748, 1524 cm^{-1}; MS *m/z* (ESI): 589 [M + H$^+$]; anal. calcd. for $C_{31}H_{32}N_4O_8$ (588.61): C, 63.26; H, 5.48; N, 9.52; found: C, 63.21; H, 5.58; N, 9.62.

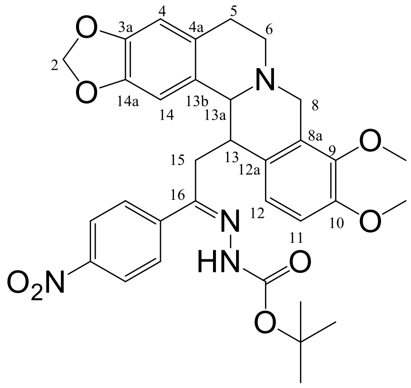

***tert*-Butyl-2-(2-(9,10-dimethoxy-6,8,13,13a-tetrahydro-5*H*-[1,3]dioxolo[4,5-*g*]isoquinolino[3,2-*a*]isoquinolin-13-yl)-1-(4-nitrophenyl)ethylidene)hydrazinecarboxylate (3h).**

3h was isolated by chromatographic column on silica gel (ethyl acetate/cyclohexane, 40/60) in 80% yield (98 mg). White amorphous solid; mp: 165–167 °C; ^{1}H NMR (400 MHz, DMSO$_{d6}$, 25 °C): 1.47 (s, 9H, C(*CH₃)₃*), 2.28–2.33 (m, 1H, *C(15)H₂*), 2.61–2.83 (m, 3H, *C(15)H₂, C(5)H2,* C(6)*H₂*), 3.05–3.12 (m, 1H, *C(5)H2*), 3.23–3.27 (m, 1H, C(6)*H₂*), 3.54 (d, 1H, *J* =16.0 Hz, *C(8)H₂*), 3.66 (s, 3H, O*CH₃*), 3.68 (s, 3H, O*CH₃*), 3.84 (brs, 1H, *C(13a)H*), 3.97–4.07 (m, 1H, *C(13)H*), 4.12 (d, 1H, *J* =16.0 Hz, *C(8)H₂*), 5.98 and 5.99 (2s, 2H, OC(2)*H₂*O), 6.38 (d, 1H, *J* =8.4 Hz, *C(12)H*), 6.55 (d, 1H, *J* =8.8 Hz, *C(11)H*), 6.72 (s, 1H, *C(4)H*), 7.00 (s, 1H, *C(14)H*), 7.88 (d, 2H, *J* =8.8 Hz, *ArH*), 8.17 (d, 2H, *J*= 8.8 Hz, *ArH*), 11.57 (brs, 1H, *NH*);^{13}C NMR (100 MHz, DMSO$_{d6}$, 25 °C): δ = 28.0 (q), 28.4 (t), 29.0 (t), 39.9 (d), 50.0 (t), 53.0 (t), 55.4 (q), 59.4 (q), 63.4

(d), 79.7 (s), 100.8 (t), 106.2 (d), 108.2 (d), 110.3 (d), 123.2 (d), 125.1 (d), 127.0 (d), 127.2 (s), 127.6 (s), 128.6 (s), 129.1 (s), 143.9 (s), 144.5 (s), 145.8 (s), 146.2 (s), 146.8 (s), 147.2 (s), 150.2 (s), 152.2 (s); IR (nujol): ν_{max} = 3246, 1730, 1638, 1526 cm^{-1}; MS *m/z* (ESI): 617 [M + H^+]; anal. calcd. for $C_{33}H_{36}N_4O_8$ (616.66): C, 64.27; H, 5.88; N, 9.09; found: C, 64.15; H, 5.92; N, 9.01.

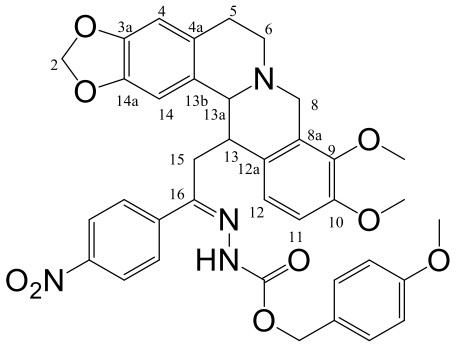

4-Methoxybenzyl 2-(2-(9,10-dimethoxy-6,8,13,13a-tetrahydro-5*H*-[1,3]dioxolo[4,5-*g*]isoquinolino[3,2-*a*]isoquinolin-13-yl)-1-(4-nitrophenyl)ethylidene)hydrazinecarboxylate (3i).

3i was isolated by chromatographic column on silica gel (ethyl acetate/cyclohexane, 40/60) in 92% yield (125 mg). White amorphous solid; mp: 175−177 °C with decomposition; ^{1}H NMR (400 MHz, DMSO$_{d6}$, 25 °C): 2.32−2.36 (m, 1H, *C(15)H$_2$*), 2.55−2.63 (m, 2H, *C(15)H$_2$, C(5)H2*), 2.81−2.87 (m, 1H, *C(5)H2*), 2.94–3.07 (m, 2H, C(6)*H$_2$*), 3.44 (d, 1H, *J* =15.6 Hz, *C(8)H$_2$*), 3.57 (s, 3H, O*CH$_3$*), 3.66 (s, 3H, O*CH$_3$*), 3.78 (s, 3H, O*CH$_3$*), 3.81 (brs, 1H, *C(13a)H*), 3.97–4.04 (m, 2H, *C(13)H* and *C(8)H$_2$*), 5.07 (s, 2H, *OCH$_2$*), 5.98–and 6.00 (2s, 2H, OC(2)*H$_2$*O), 6.39 (d, 1H, *J* =8.4 Hz, *C(12)H*), 6.56 (d, 1H, *J* =8.8 Hz, *(11)H*), 6.71 (s, 1H, *C(4)H*), 6.97–6.99 (m, 3H, *C(14)H, ArH*), 7.37 (d, 2H, *J* =8.4 Hz, *ArH*), 7.87 (d, 2H, *J* =8.8 Hz, *ArH*), 8.17 (d, 2H, *J* =8.8 Hz, *ArH*), 11.94 (brs, 1H, *NH*); ^{13}C NMR (100 MHz, DMSO$_{d6}$, 25 °C): δ = 28.4 (t), 29.1 (t), 39.9 (d), 49.6 (t), 52.9 (t), 55.1 (q), 55.5 (q), 59.2 (q), 63.3 (d), 66.2 (t), 100.7 (t), 106.2 (d), 108.1 (d), 110.3 (d), 113.8 (d), 123.2 (d), 125.1 (d), 127.2 (d), 127.3 (s), 127.5 (s), 128.2 (s), 128.6 (s), 129.1 (s), 130.1 (d), 143.8 (s), 144.3 (s), 145.7 (s), 146.2 (s), 146.9 (s), 148.4 (s), 150.2 (s), 153.4 (s), 159.2 (s); IR (nujol): ν_{max} = 3186, 1721, 1587 cm^{-1}; HRMS (ESI) calcd. for $C_{37}H_{37}N_4O_9$ [M + H]$^+$: 681.2561; found: 681.2522.

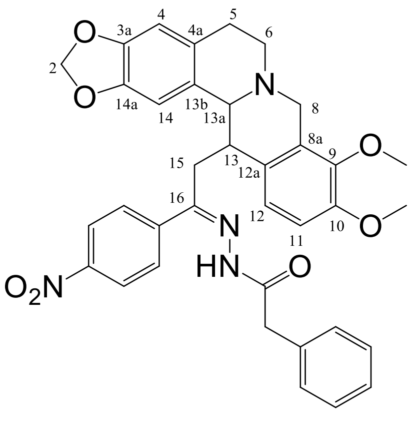

N'-(2-(9,10-Dimethoxy-6,8,13,13a-tetrahydro-5*H*-[1,3]dioxolo[4,5-g]isoquinolino[3,2-*a*]isoquinolin-13-yl)-1-(4-nitrophenyl)ethylidene)-2-phenylacetohydrazide (3j).

3j was isolated by chromatographic column on silica gel (ethyl acetate/cyclohexane, 40/60) in 94% yield (119 mg). White amorphous solid; mp: 175–176 °C with decomposition; ^{1}H NMR (400 MHz, DMSO$_{d6}$, 25 °C): 2.52–2.60 (m, 3H, *C(15)H$_2$*, *C(5)H2*, C(6)*H$_2$*), 2.82–2.88 (m, 1H, *C(15)H$_2$*), 3.01–3.09 (m, 1H, *C(5)H$_2$*), 3.23–3.27 (m, 1H, C(6)*H$_2$*), 3.48 (d, 1H, *J* =13.2 Hz, *C(8)H$_2$*), 3.50 (s, 3H, O*CH$_3$*), 3.64–3.73 (m, 2H, *CH$_2$*), 3.69 (s, 3H, O*CH$_3$*), 3.82 (brs, 1H, *C(13a)H*), 3.94–3.96 (m, 1H, *C(13)H*), 4.10 (d, 1H, *J* =16.0 Hz, *C(8)H$_2$*), 5.98 and 6.01 (2s, 2H, OC(2)*H$_2$*O), 6.45 (d, 1H, *J* =8.4 Hz, *C(12)H*), 6.01 (d, 1H, *J* =8.4 Hz, *C(11)H*), 6.69 (s, 1H, *C(4)H*), 6.95 (s, 1H, *C(14)H*), 7.06–7.08 (m, 2H, *ArH*), 7.16–7.24 (m, 3H, *ArH*), 7.90 (d, 2H, *J* =9.2Hz, *ArH*), 8.18 (d, 2H, *J* =9.2 Hz, *ArH*), 11.66 (s, 1H, *NH*); ^{13}C NMR (100 MHz, DMSO$_{d6}$, 25 °C): δ = 28.5 (t), 29.1 (t), 38.5 (t), 40.2. (d), 49.6 (t), 53.0 (t), 55.5 (q), 59.1 (q), 62.8 (d), 100.7 (t), 106.2 (d), 108.0 (d), 110.4 (d), 123.2 (d), 124.7 (d), 126.1 (d), 127.2 (d), 127.5 (d), 127.6 (s), 128.0 (d), 128.1 (s), 129.0 (s), 129.1 (s), 135.4 (s), 144.0 (s), 144.2 (s), 145.7 (s), 146.1 (s), 147.0 (s), 147.0 (s), 150.1 (s), 172.9 (s); IR (nujol): ν_{max} = 3060, 1684, 1583 cm^{-1}; MS *m/z* (ESI): 635 [M + H$^+$]; anal. calcd. for $C_{36}H_{34}N_4O_7$ (634.68): C, 68.13; H, 5.40; N, 8.83; found: C, 67.99; H, 5.44; N, 8.89.

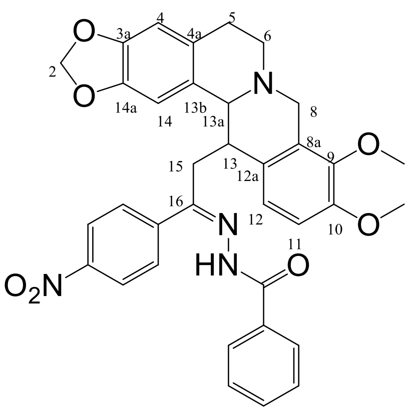

N'-(2-(9,10-Dimethoxy-6,8,13,13a-tetrahydro-5*H*-[1,3]dioxolo[4,5-*g*]isoquinolino[3,2-*a*]isoquinolin-13-yl)-1-(4-nitrophenyl)ehylidene)benzohydrazide (3k).

3k was isolated by chromatographic column on silica gel (ethyl acetate/cyclohexane, 40/60) in 94% yield (116 mg). White amorphous solid; mp: 176–178 °C with decomposition; ^{1}H NMR

(400 MHz, DMSO*d6*, 25 °C): 2.38–2.46 (m, 1H, *C(15)H2*), 2.56–2.69 and 2.75–2.83 (2m, 3H, *C(15)H2*, *C(5)H2*), C(6)*H2*), 2.93–2.99 (m, 1H, *C(5)H2*), 3.09–3.19 (m, 1H, C(6)*H2*), 3.30 (d, 1H, *J*= 14.4 Hz, *C(8)H2*), 3.50 (s, 3H, O*CH3*), 3.60–3.64 (m, 1H, *C(13a)H*), 3.64 (s, 3H, O*CH3*), 3.69 (brs, 1H, *C(8)H2*), 3.76–3.83 (m, 1H, *C(13)*), 5.97 and 6.00 (2s, 2H, OC(2)*H2*O), 6.46 (d, 1H, *J* =8.8 Hz, *C(12)H*), 6.65 (d, 1H, *J* =8.4 Hz, *C(11)H*), 6.73 (s, 1H, *C(4)H*), 7.01 (brs, 1H, *C(14)H*), 7.29–7.46 (m, 3H, *ArH*), 7.51–7.75 (m, 6H, *ArH*), 10.39 (brs, 1H, *NH*);^{13}C NMR (100 MHz, DMSO*d6*, 25 °C): δ = 28.7 (t), 29.6 (t), 39.9 (d), 50.5 (t), 53.8 (t), 55.5 (q), 59.3 (q), 63.3 (d), 100.7 (t), 106.1 (d), 108.2 (d), 110.6 (d), 124.8 (s), 126.5 (d), 127.0 (d), 127.4 (s), 127.8 (d), 128.3 (d), 128.6 (d), 129.2 (d), 129.5 (s), 131.7 (s), 134.2 (s), 137.8 (s), 144.0 (s), 145.7 (s), 146.2 (s), 150.2 (s), 154.2 (s), 163.1 (s); IR (nujol): ν_{max} = 3184, 1705, 1648 cm^{-1}; MS *m/z* (ESI): 621 [M + H$^+$]; anal. calcd. for $C_{35}H_{32}N_4O_7$ (620.65): C, 67.73; H, 5.20; N, 9.03; found: C, 67.88; H, 5.14; N, 8.95.

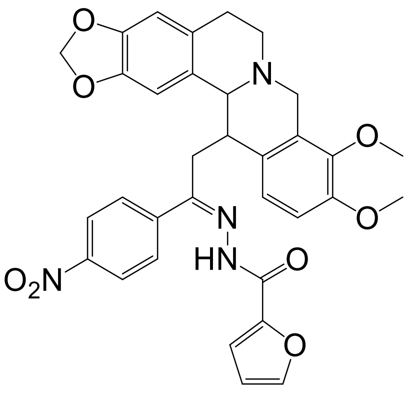

N'-(2-(9,10-Dimethoxy-6,8,13,13a-tetrahydro-5*H*-[1,3]dioxolo[4,5-*g*]isoquinolino[3,2-*a*]isoquinolin-13-yl)-1-(4-nitrophenyl)ethylidene)furan-2-carbohydrazide (3l).

3l was isolated by chromatographic column on silica gel (ethyl acetate/cyclohexane, 40/60) in 77% yield (94 mg). White amorphous solid; mp: 181–183 °C with decomposition; ^{1}H NMR (400 MHz, DMSO*d6*, 25 °C): 2.51–2.94 (m, 4H, *C(15)H2*, *C(5)H2*, C(6)*H2*), 3.04–3.10 (m, 1H, *C(5)H2*), 3.17–3.28 (m, 1H, C(6)*H2*), 3.48 (d, 1H, *J* =16.0 Hz, *C(8)H2*), 3.56 (s, 3H, O*CH3*), 3.66 (s, 3H, O*CH3*), 3.78 (brs, 1H, *C(10)H*), 3.89–3.97 (m, 1H, *C(13)H*), 4.09 (d, 1H, *J* =16.0 Hz, *C(8)H2*), 5.94 and 5.96 (2s, 2H, OC(2)*H2*O), 6.49–6.57 (m, 1H, *C(12)H*), 6.62–6.79 (m, 3H, *C(11)H, furan*), 6.94 (s, 1H, *C(4)H*), 7.20 (brs, 1H, *C(14)H*), 7.89 (d, 2H, *J* =8.8 Hz, *ArH*), 8.01 (brs, 1H, *furan*), 8.21 (d, 2H, *J* =9.2 Hz, *ArH*), 10.74 (brs, 1H, *NH*);^{13}C NMR (100 MHz, DMSO*d6*, 25 °C): δ = 28.5 (t), 29.9 (t), 39.9 (d), 50.5 (t), 53.6 (t), 55.6 (q), 59.2 (q), 63.0 (d), 100.7 (t), 106.1 (d), 108.1 (d),

110.7 (d), 112.4 (d), 123.2 (d), 124.5 (d), 124.6 (s), 127.4 (s), 127.5 (d), 127.5 (s), 129.1 (s), 129.8 (s), 143.7 (s), 144.2 (s), 145.7 (s), 146.1 (s), 147.3 (s), 150.3 (s), 152.8 (s), 170.1 (s); IR (nujol): ν_{max} = 3252, 1741, 1565 cm^{-1}; HRMS (ESI) calcd. for $C_{33}H_{31}N_4O_8$ $[M + H]^+$: 611.2142; found: 611.2167.

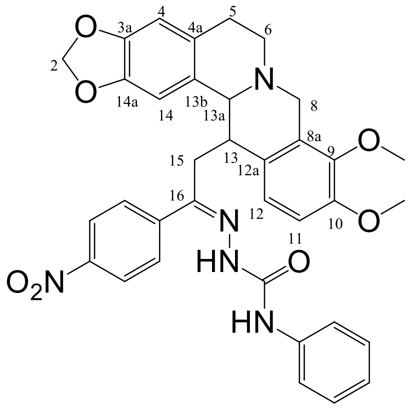

2-(2-(9,10-Dimethoxy-6,8,13,13a-tetrahydro-5*H*-[1,3]dioxolo[4,5-*g*]isoquinolino[3,2-*a*]isoquinolin-13-yl)-1-(4-nitrophenyl)ethylidene)-*N*-phnylhydrazinecarboxamide (3m). **3m** was isolated by chromatographic column on silica gel (ethyl acetate/cyclohexane, 40/60) in 68% yield (87 mg). White amorphous solid; mp: 181−182 °C with decomposition; ^{1}H NMR (400 MHz, DMSO$_{d6}$, 25 °C): 2.63–2.71 (m, 3H, *C(15)H$_2$*, *C(5)H$_2$*, C(6)*H$_2$*), 2.82–2.90 (m, 1H, *C(15)H$_2$*), 3.06–3.18 (m, 1H, *C(5)H$_2$*), 3.49–3.56 (m, 2H, C(6)*H$_2$*) and *C(8)H$_2$*), 3.60–3.65 (m, 6H, O*CH$_3$* and O*CH$_3$*), 3.86–3.88 (m, 1H, *C(13a)H*), 4.02–4.05 (m, 1H, *C(13)H*), 4.21–4.27 (m, 1H, *C(8)H$_2$*), 5.98 and 6.02 (2s, 2H, OC(2)*H$_2$*O), 6.47–6.52 (m, 1H, *J* =8.4 Hz, *C(12)H*), 6.57–6.62 (d, 1H, *J* =8.4 Hz, *C(11)H*), 6.71–6.74 (s, 1H, *C(4)H*), 6.99–7.05 (m, 2H, *C(14)H* and *ArH*), 7.26–7.28 (m, 2H, *ArH*), 7.56–7.60 (m, 2H, 9,2Hz, *ArH*), 8.12–8.27 (m, 4H, *ArH*), 8.63–8.66 (m, 1H, *NH*), (11.27 (s, 1H, *NH*);^{13}C NMR (100 MHz, DMSO$_{d6}$, 25 °C): δ = 28.5 (t), 29.3 (t), 39.9 (d), 49.5 (t), 52.9 (t), 55.4 (q), 59.2 (q), 62.9 (d), 100.6 (t), 106.1 (d), 108.0 (d), 110.3 (d), 119.8 (d), 122.5 (s), 122.9 (d), 124.8 (d), 127.4 (d), 127.4 (s), 127.5 (d), 128.1 (s), 128.3 (d), 128.9 (s), 129.1(s), 138.6 (s), 144.0 (s), 145.0 (s), 145.7 (s), 146.1 (s), 146.7 (s), 150.1 (s), 153.0 (s); IR (nujol): ν_{max} = 3251, 1730, 1676 cm^{-1}; MS *m/z* (ESI): 636 $[M + H^+]$; anal. calcd. for $C_{35}H_{33}N_5O_7$ (635.67): C, 66.13; H, 5.23; N, 11.02; found: C, 66.23; H, 5.19; N, 10.94.

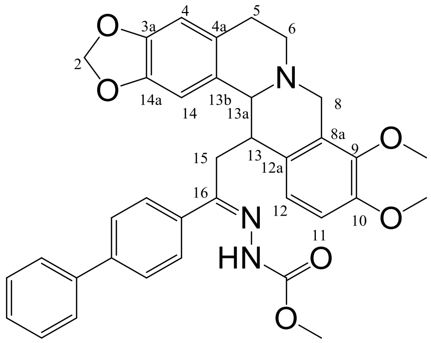

Methyl-2-(1-([1,1'-biphenyl]-4-yl)-2-(9,10-dimethoxy-6,8,13,13a-tetrahydro-5*H*-[1,3]dioxolo[4,5-*g*]isoquinolino[3,2-*a*]isoquinolin-13-yl)ethylidene)hydrazinecarboxylate (3n).

3n was isolated by chromatographic column on silica gel (ethyl acetate/cyclohexane, 40/60) in 96% yield (115 mg). White amorphous solid; mp: 175–176 °C; ^{1}H NMR (400 MHz, DMSO$_{d6}$, 25 °C): 2.41–2.46 (m, 1H, *C(15)H$_2$*), 2.61–2.76 (m, 3H, *C(15)H$_2$*, *C(5)H2*, C(6)*H$_2$*), 3.03–3.12 (m, 1H, C(6)*H$_2$*), 3.24–3.28 (m, 1H, *C(5)H$_2$*), 3.54 (d, 1H, *J* =15.6 Hz, *C(8)H$_2$*), 3.66 (2s, 6H, *OCH$_3$*), 3.67 (s, 3H, O*CH$_3$*), 3.85 (brs, 1H, *C(13a)H*), 3.95–3.99 (m, 1H, *C(13)H*), 4.14 (d, 1H, *J* =16.0 Hz, *C(8)H$_2$*), 5.99 and 6.01 (2s, 2H, OC(2)*H$_2$*O), 6.48 (d, 1H, *J* =8.4 Hz, *C(12)H*), 6.61 (d, 1H, *J* =8.4 Hz, *C(11)H*), 6.74 (s, 1H, *C(4)H*), 7.04 (s, 1H, *C(14)H*), 7.36–7.40 (m, 1H, *ArH*), 7.47–7.50 (m, 2H, *ArH*), 7.65–7.73 (m, 6H, *ArH*), 11.18 (s, 1H, *NH*); ^{13}C NMR (100 MHz, DMSO$_{d6}$, 25 °C): δ = 28.7 (t), 29.2 (t), 39.9 (d), 49.9 (t), 52.0 (q), 53.2 (t), 55.5 (q), 59.3 (q), 63.3 (d), 100.7 (t), 106.2 (d), 108.1 (d), 110.3 (d), 125.1 (d), 126.2 (d), 126.5 (d), 126.7 (d), 127.5 (d), 127.6 (s), 127.7 (d), 128.9 (s), 129.1 (s), 130.0 (s), 137.2 (s), 139.4 (s), 139.9 (s), 143.9 (s), 145.7 (s), 146.2 (s), 148.8 (s), 149.7 (s), 150.2 (s); IR (nujol): ν_{max} = 3429, 3317, 3238, 1758, 1708, 1666 cm^{-1}; MS *m/z* (ESI): 606 [M + H$^+$]; anal. calcd. for $C_{36}H_{35}N_3O_6$ (605.68): C, 71.39; H, 5.82; N, 6.94; found: C, 71.25; H, 5.87 N, 6.99.

3. ^{1}H and ^{13}C NMR spectra of products 2a–n

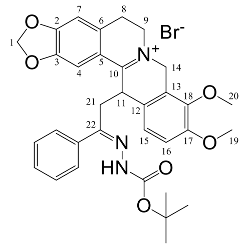

13-(2-(2-(*tert*-Butoxycarbonyl)hydrazono)-2-phenylethyl)-9,10-dimethoxy-5,6,8,13-tetrahydro-[1,3]dioxolo[4,5-*g*]isoquinolino[3,2-*a*]isoquinolin-7-ium bromide (2a)

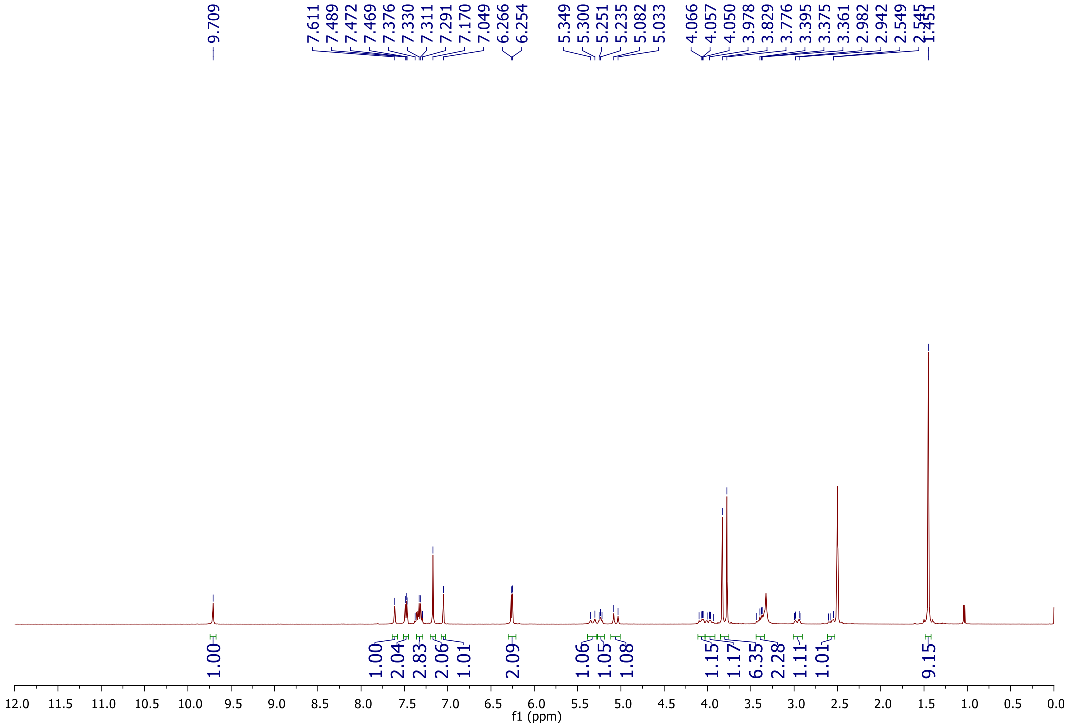

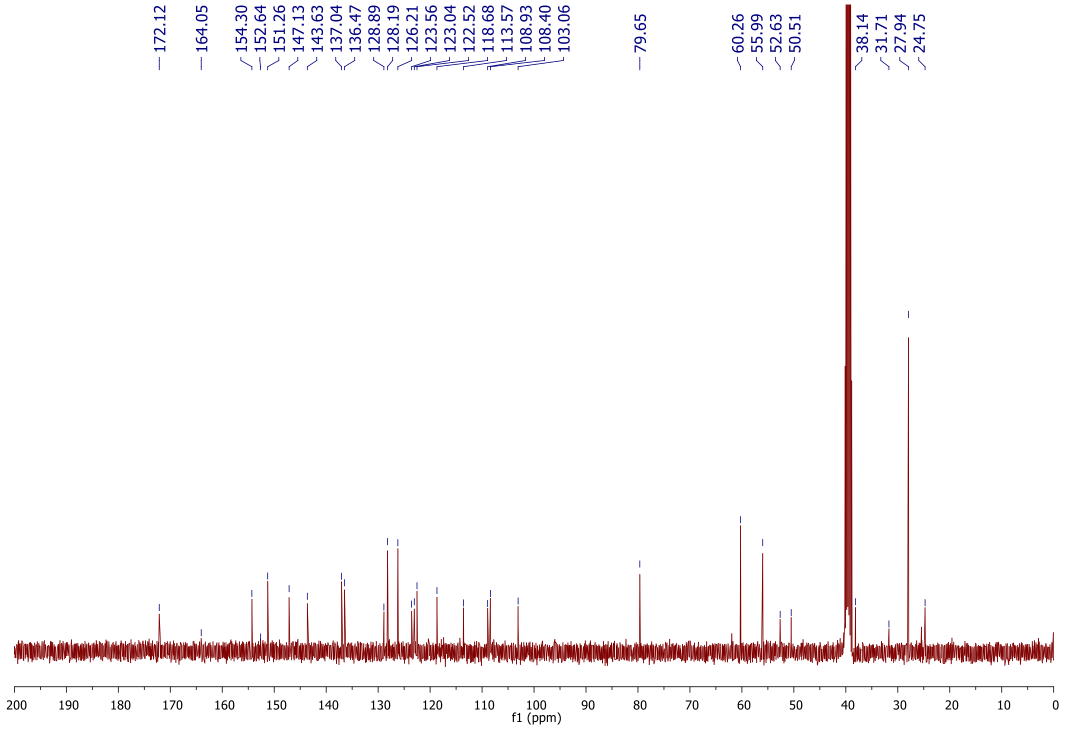

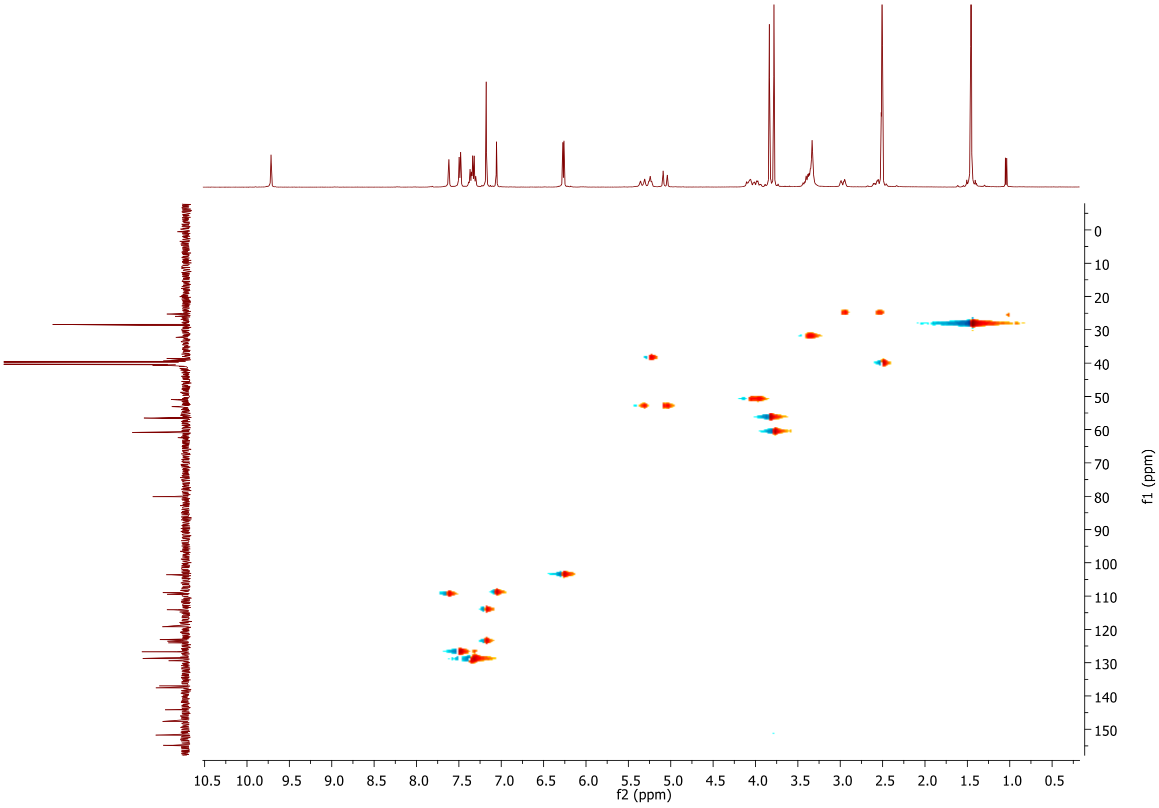

f2 (ppm)
f1 (ppm)

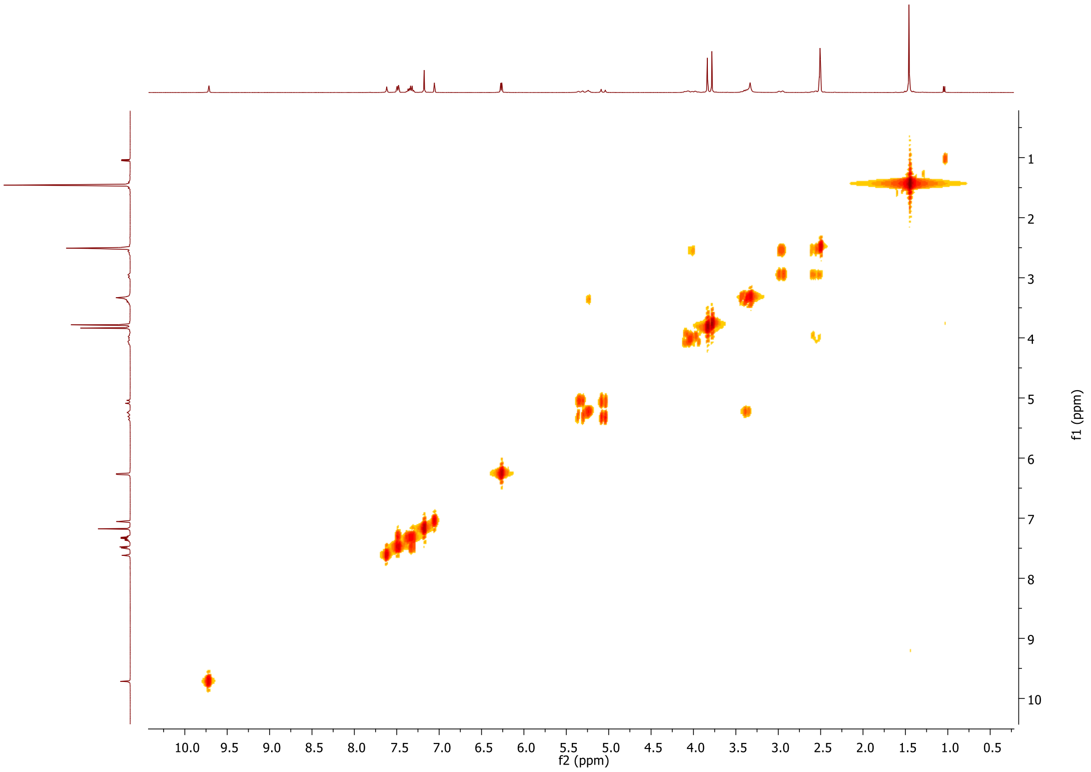

f2 (ppm)
f1 (ppm)

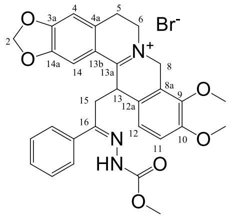

9,10-Dimethoxy-13-(2-(2-(methoxycarbonyl)hydrazono)-2-phenylethyl)-5,6,8,13-tetrahydro-[1,3]dioxolo[4,5-*g*]isoquinolino[3,2-*a*]isoquinolin-7-ium bromide (2b)

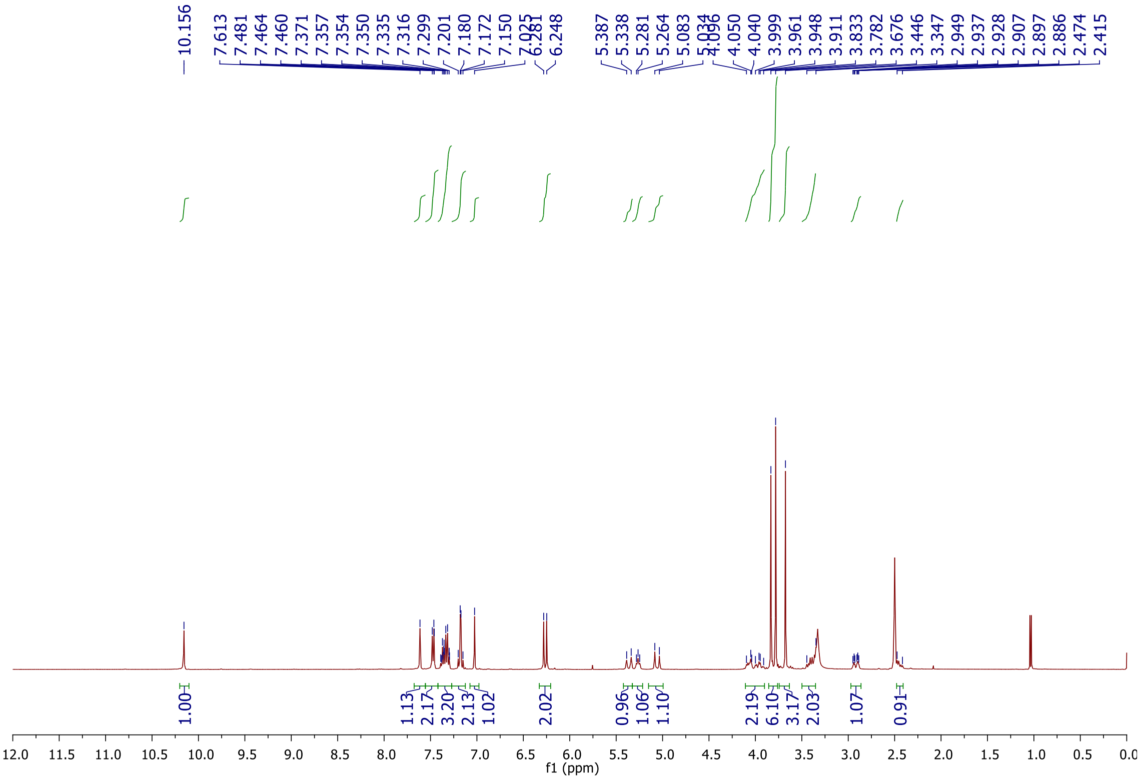

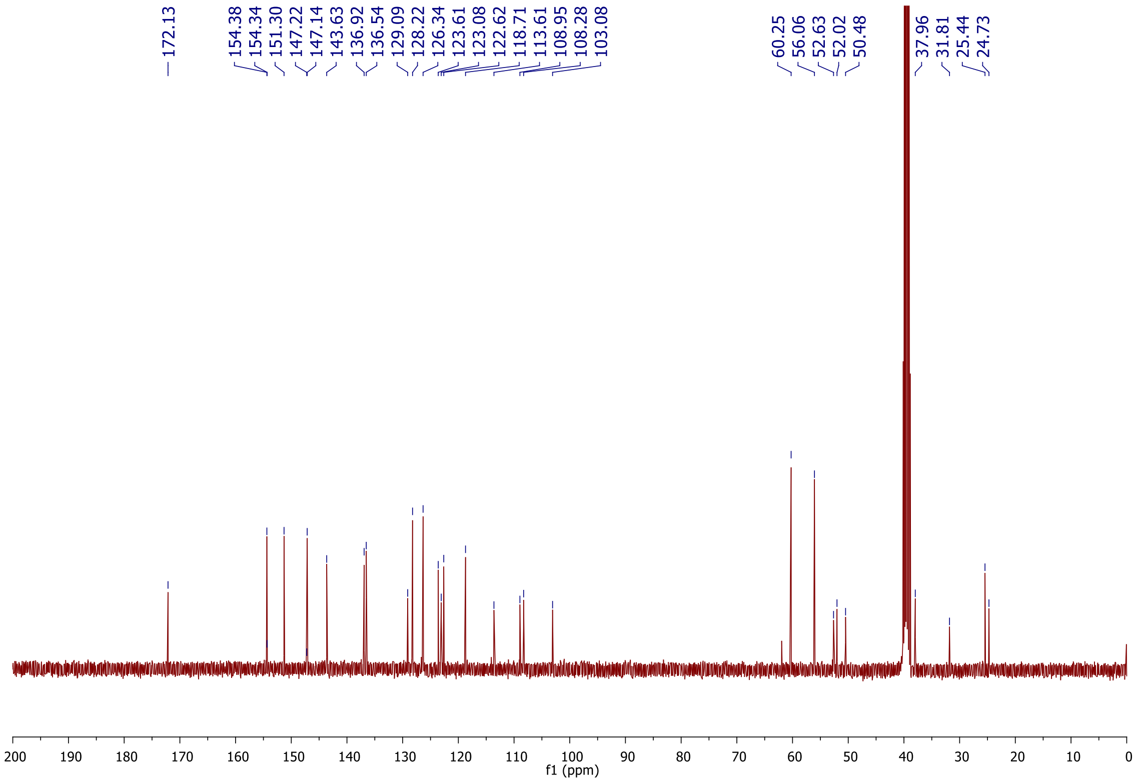

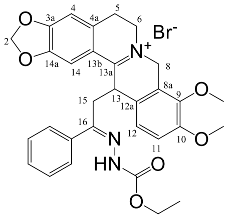

13-(2-(2-(Ethoxycarbonyl)hydrazono)-2-phenylethyl)-9,10-dimethoxy-5,6,8,13-tetrahydro-[1,3]dioxolo[4,5-*g*]isoquinolino[3,2-*a*]isoquinolin-7-ium bromide (2c)

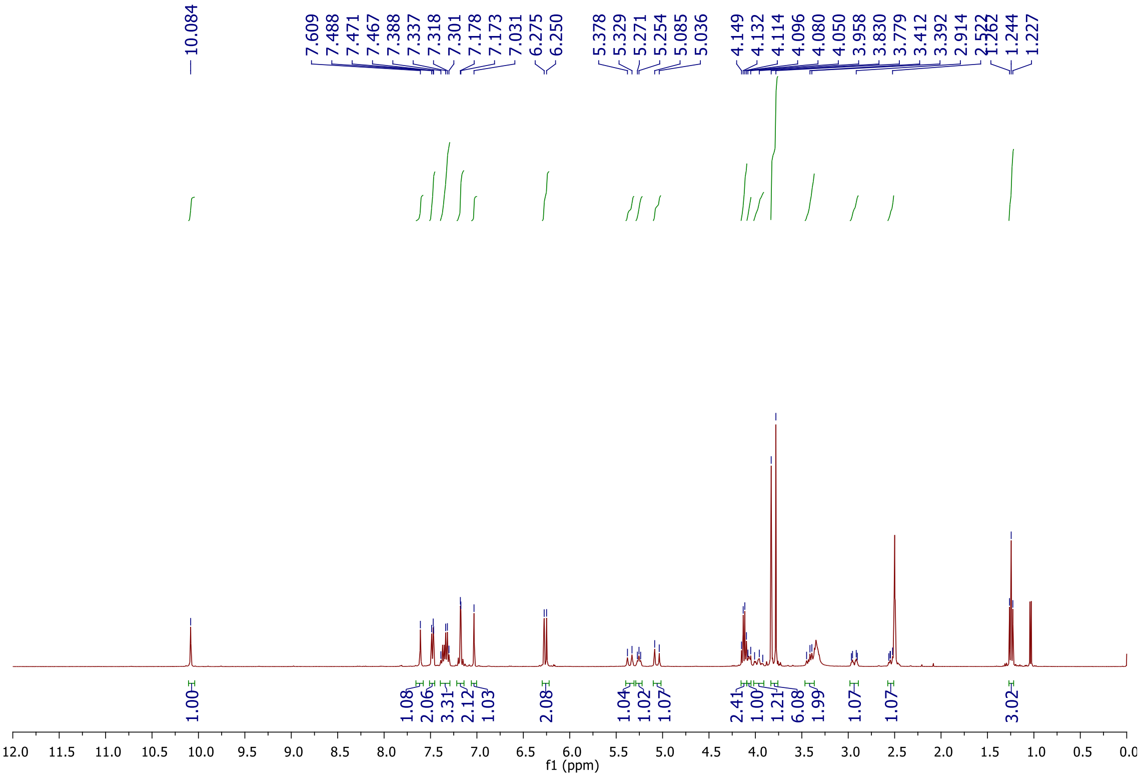

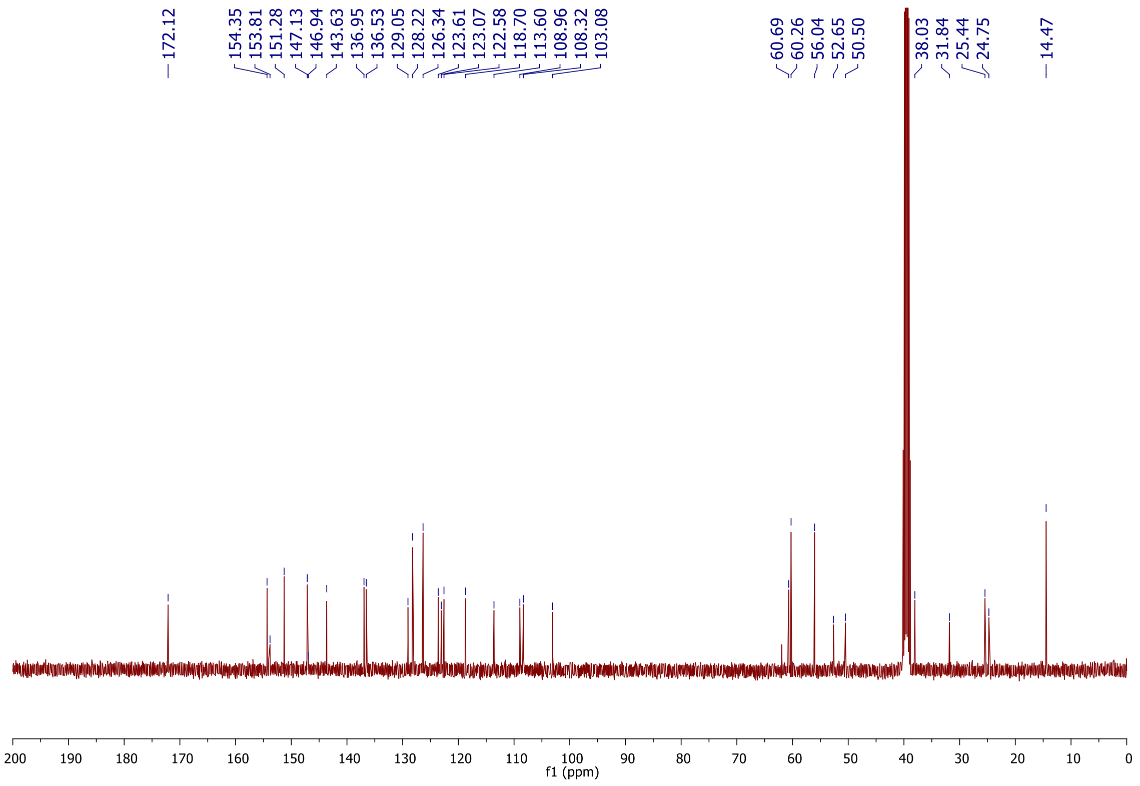

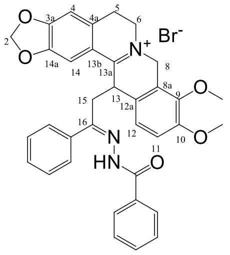

13-(2-(2-Benzoylhydrazono)-2-phenylethyl)-9,10-dimethoxy-5,6,8,13-tetrahydro-[1,3]dioxolo[4,5-*g*]isoquinolino[3,2-*a*]isoquinolin-7-ium bromide (2d)

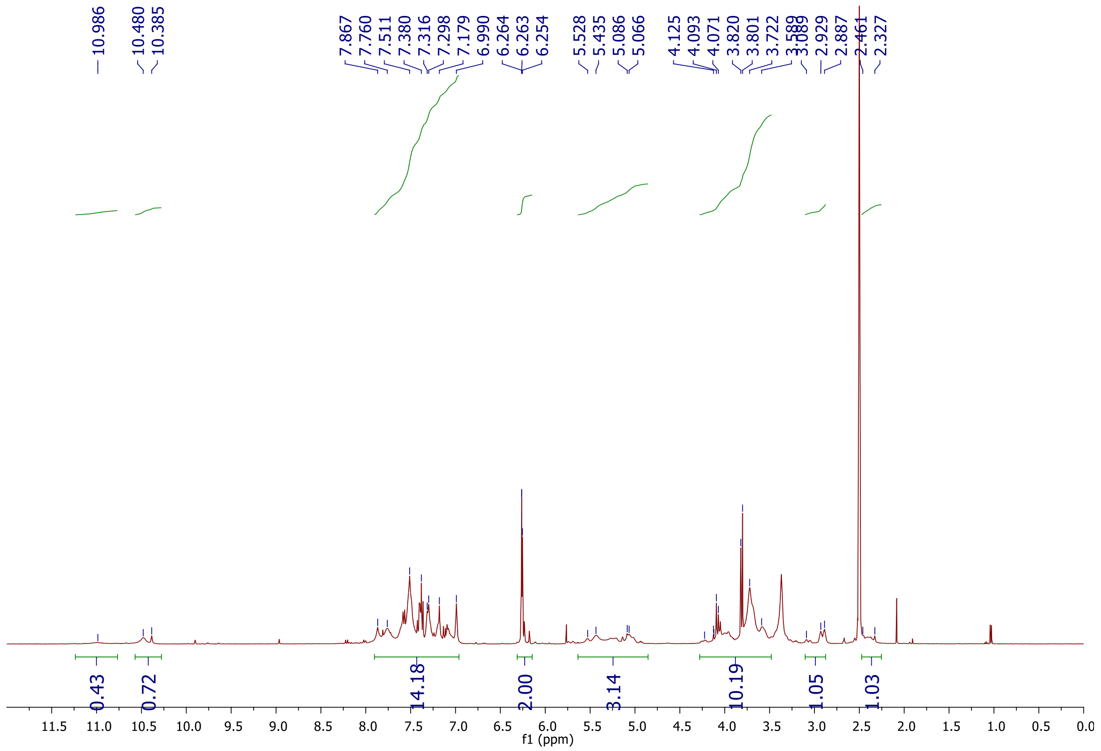

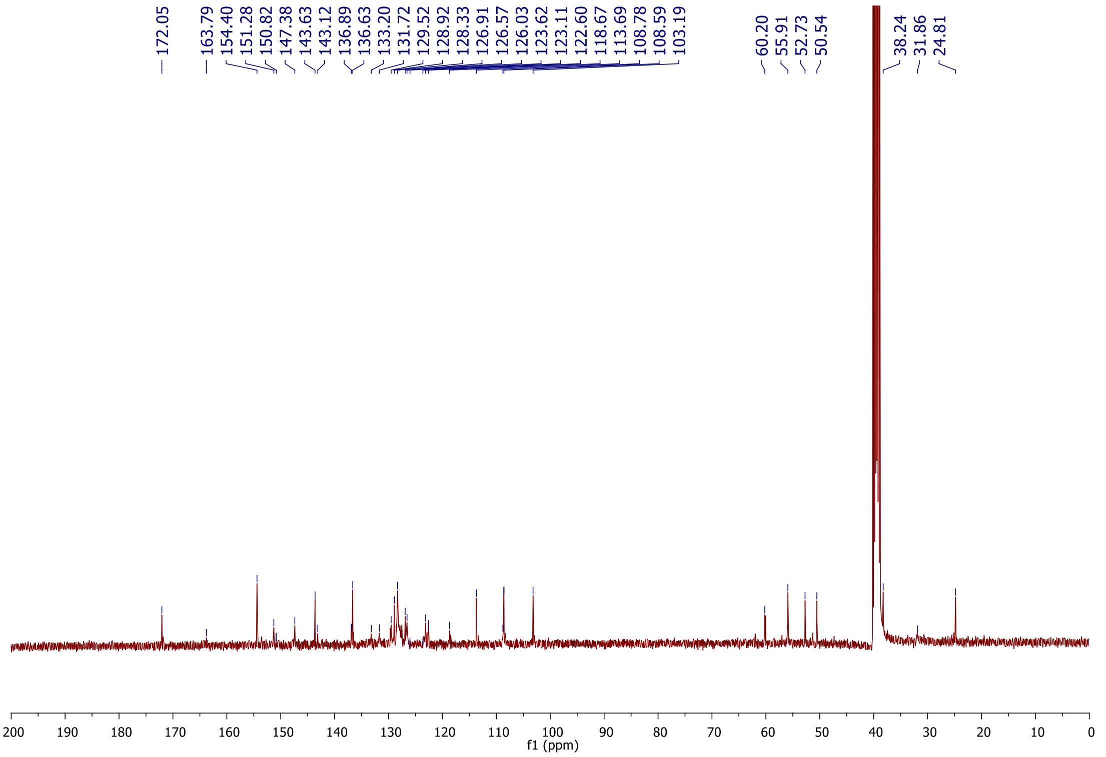

9,10-Dimethoxy-13-(2-phenyl-2-(2-(phenylcarbamoyl)-hydrazono)ethyl)-5,6,8,13-tetrahydro-[1,3]dioxolo[4,5-*g*]isoquinolino[3,2-*a*]isoquinolin-7-ium bromide (2e)

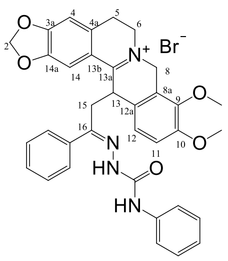

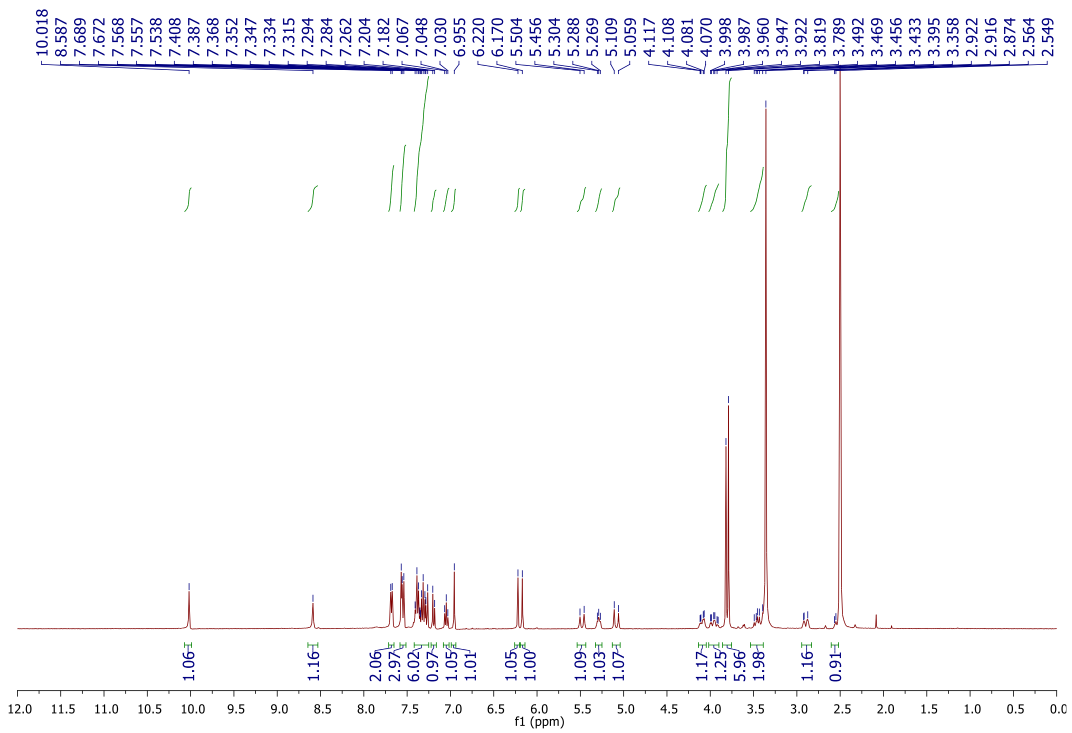

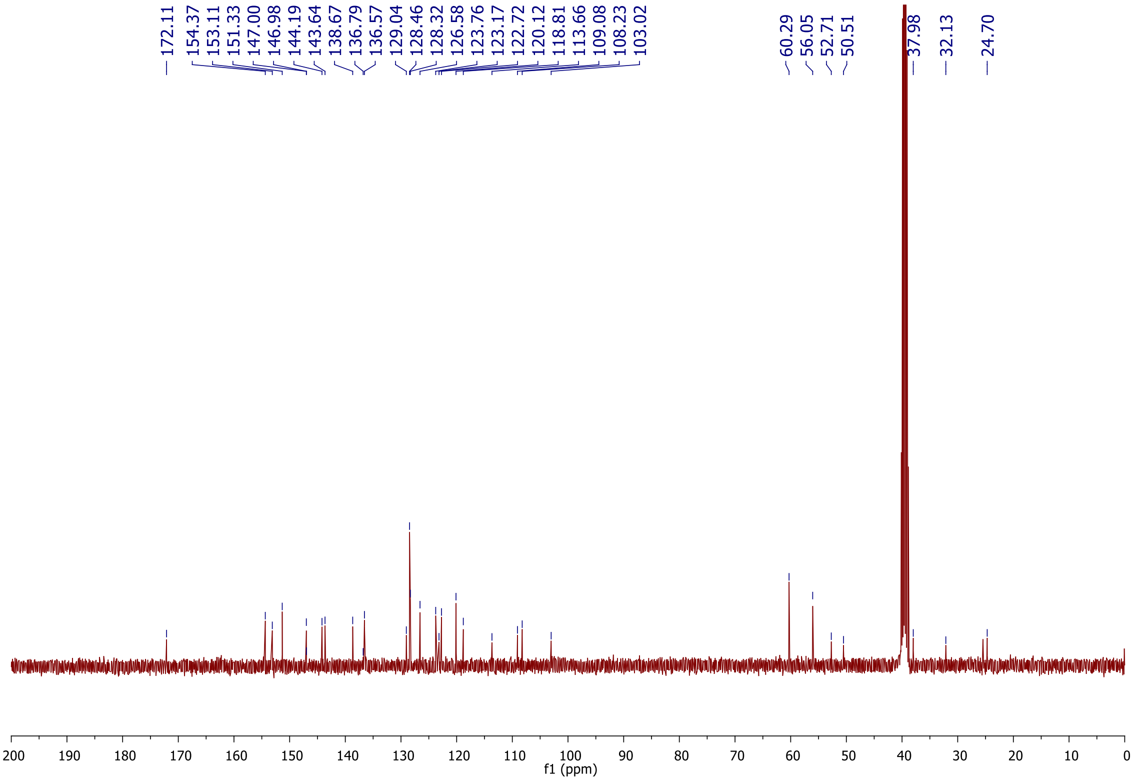

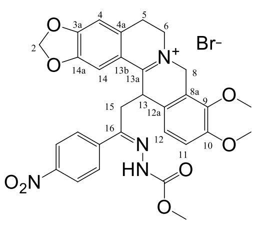

9,10-Dimethoxy-13-(2-(2-(methoxycarbonyl)hydrazono)-2-(4-nitrophenyl)ethyl)-5,6,8,13-tetrahydro-[1,3]dioxolo[4,5-*g*]isoquinolino[3,2-*a*]isoquinolin-7-ium bromide 2f

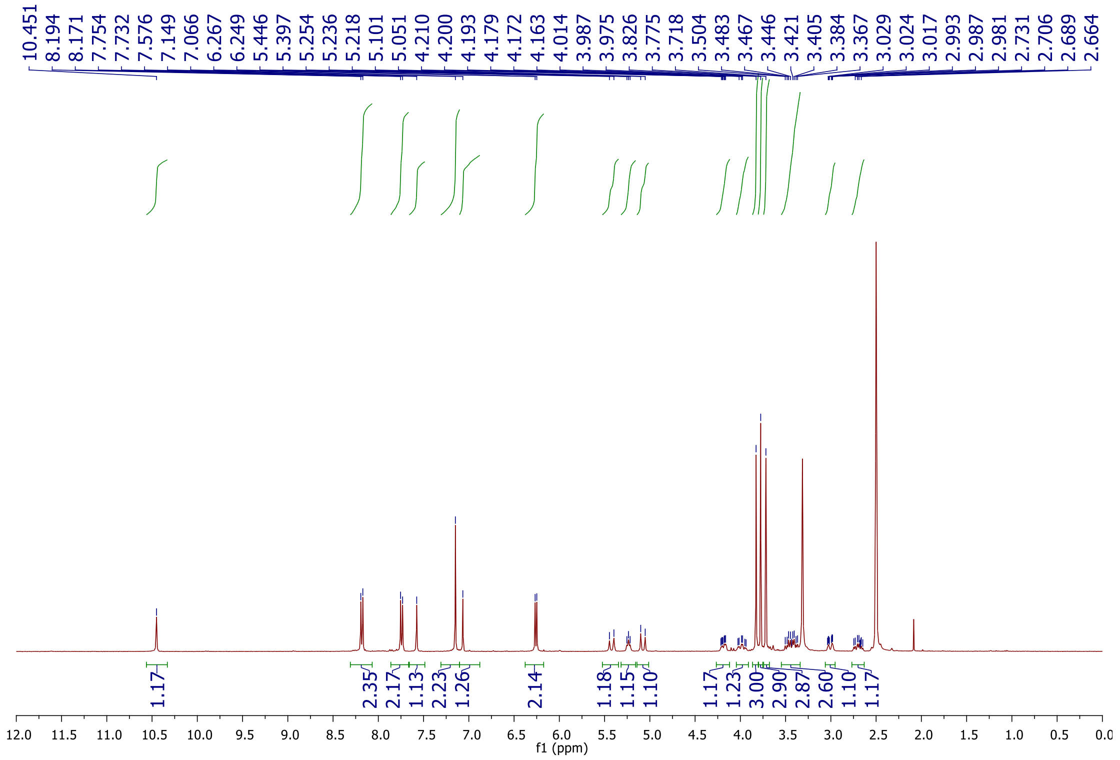

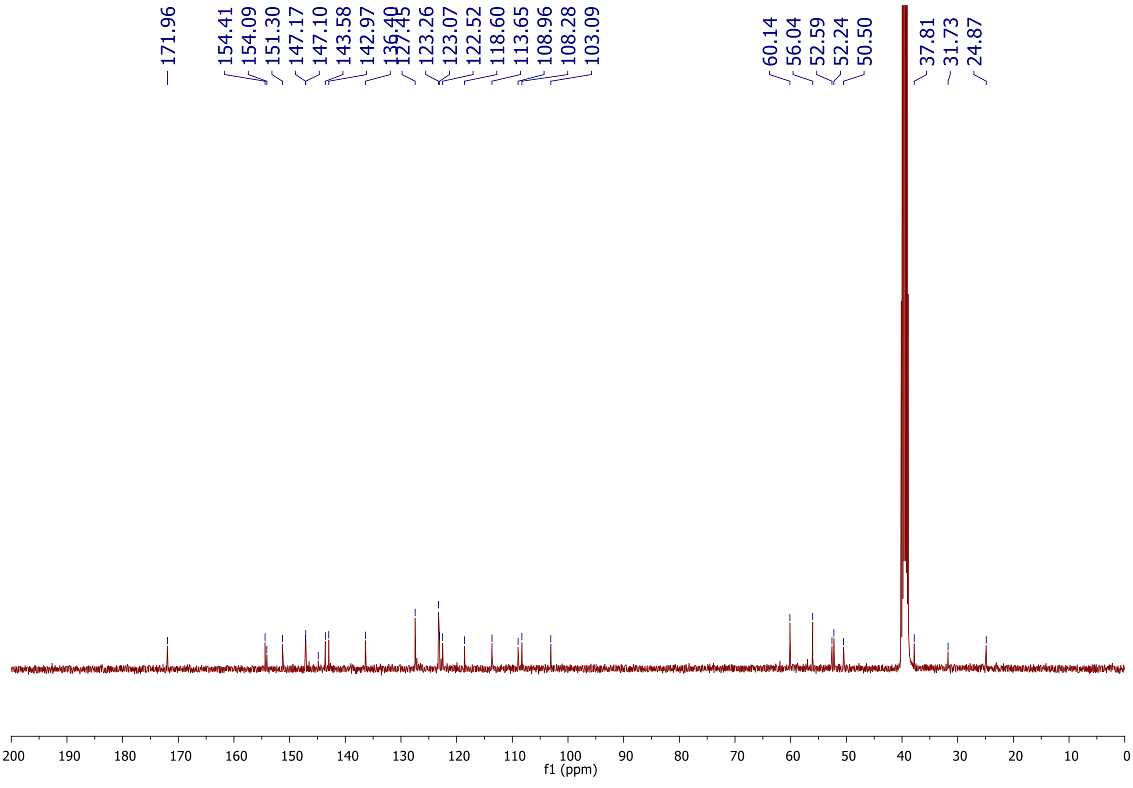

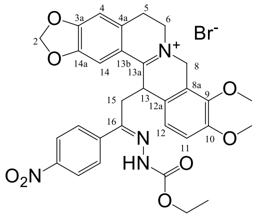

9,10-Dimethoxy-13-(2-(2-(methoxycarbonyl)hydrazono)-2-(4-nitrophenyl)ethyl)-5,6,8,13-tetrahydro-[1,3]dioxolo[4,5-*g*]isoquinolino[3,2-*a*]isoquinolin-7-ium bromide (2g)

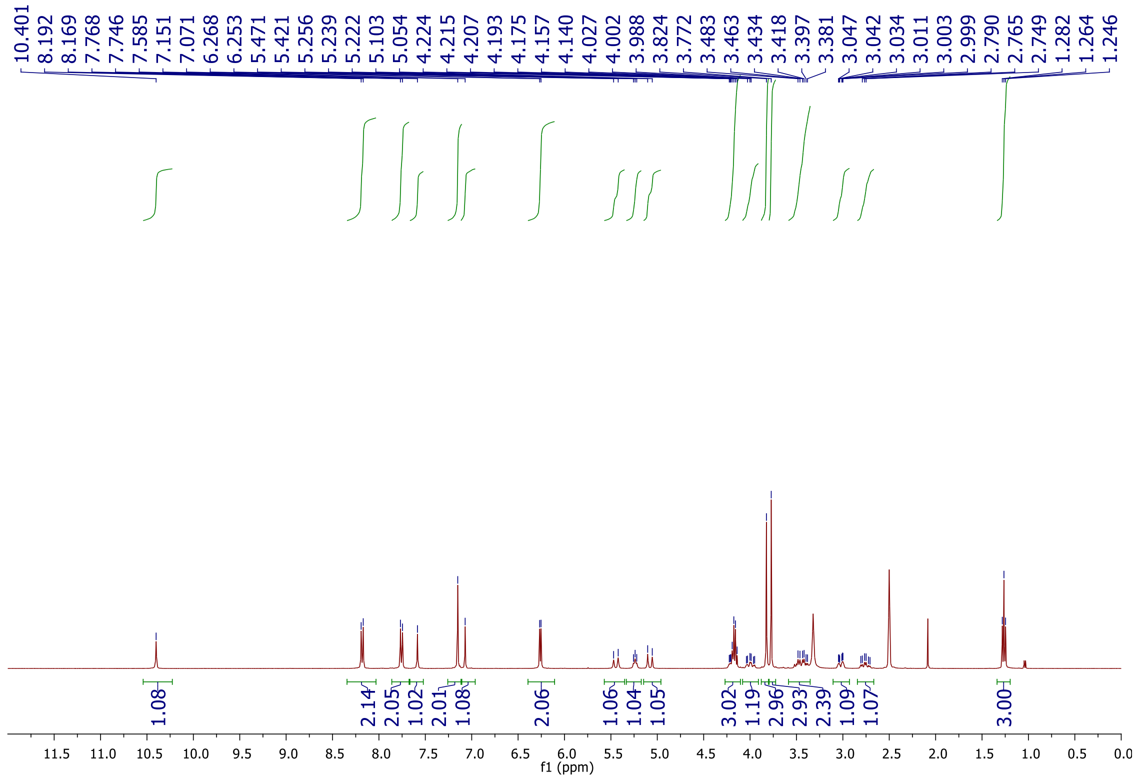

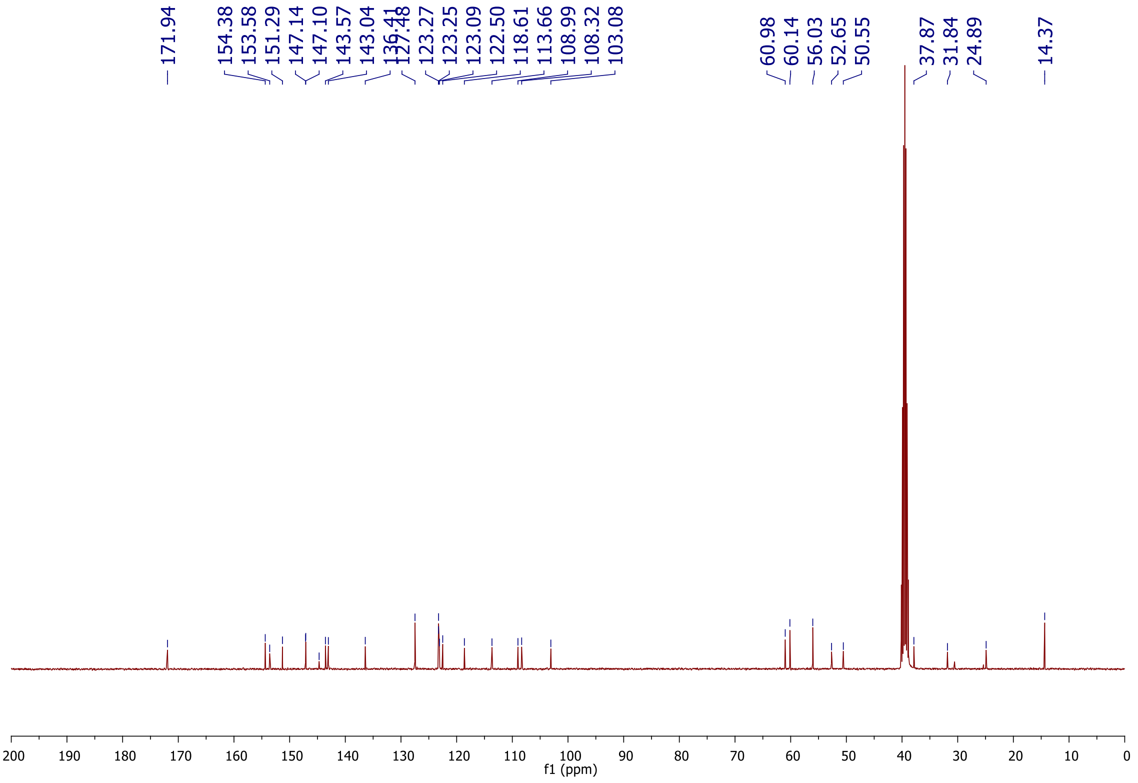

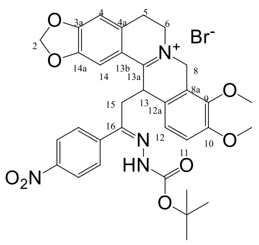

13-(2-(2-(*tert*-Butoxycarbonyl)hydrazono)-2-(4-nitrophenyl)ethyl)-9,10-dimethoxy-5,6,8,13-tetrahydro-[1,3]dioxolo[4,5-*g*]isoquinolino[3,2-*a*]isoquinolin-7-ium bromide (2h)

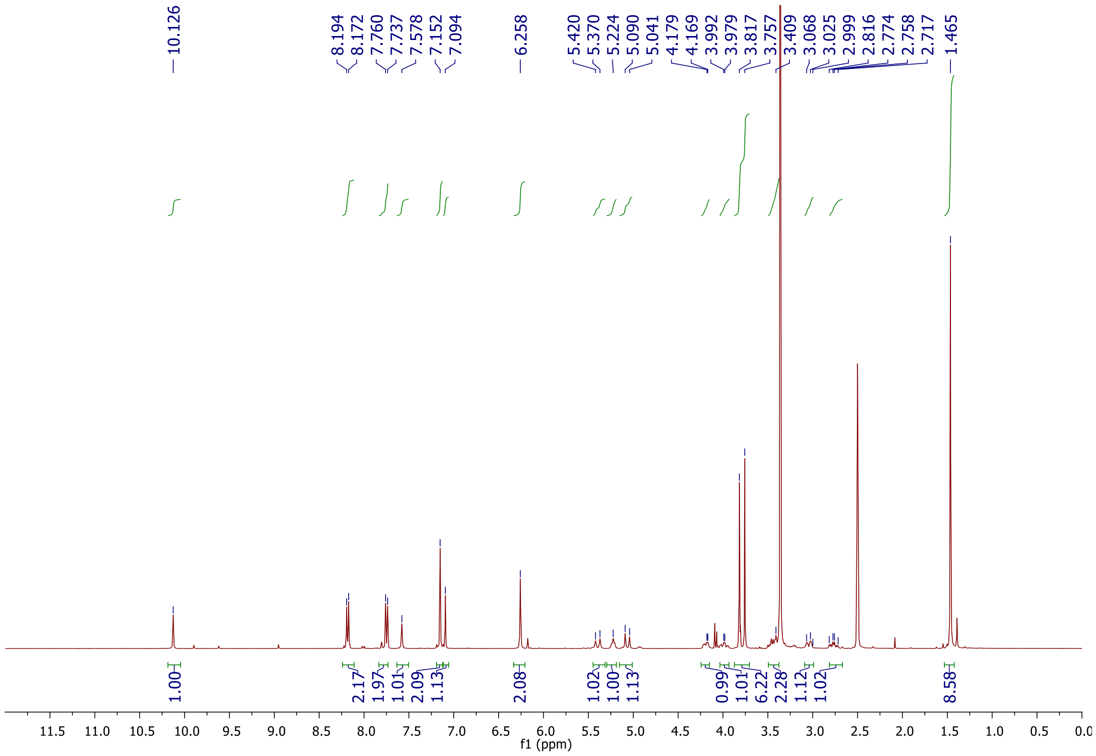

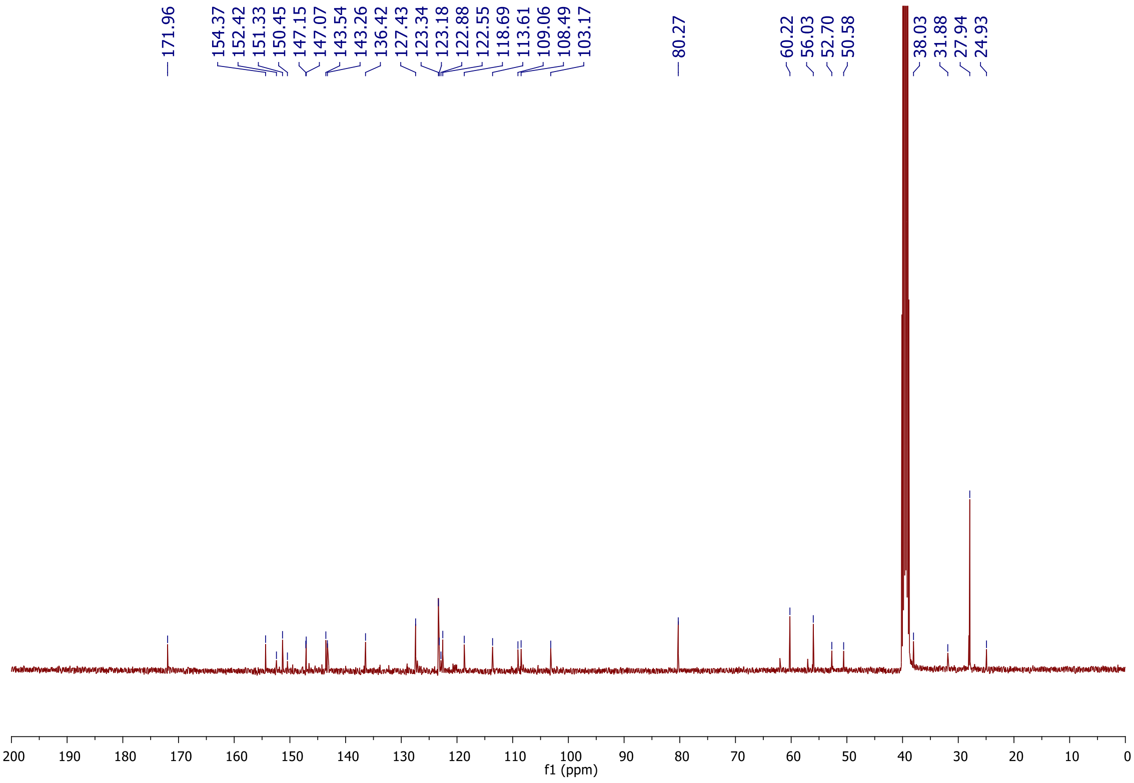

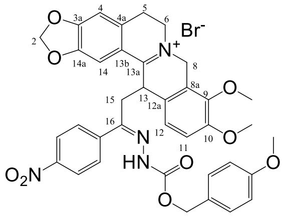

9,10-Dimethoxy-13-(2-(2-(((4-methoxybenzyl) oxy)carbonyl)hydrazono)-2-(4-nitrophenyl)ethyl)-5,6,8,13-tetrahydro-[1,3]dioxolo[4,5-*g*]isoquinolino[3,2-*a*]isoquinolin-7-ium bromide (2i)

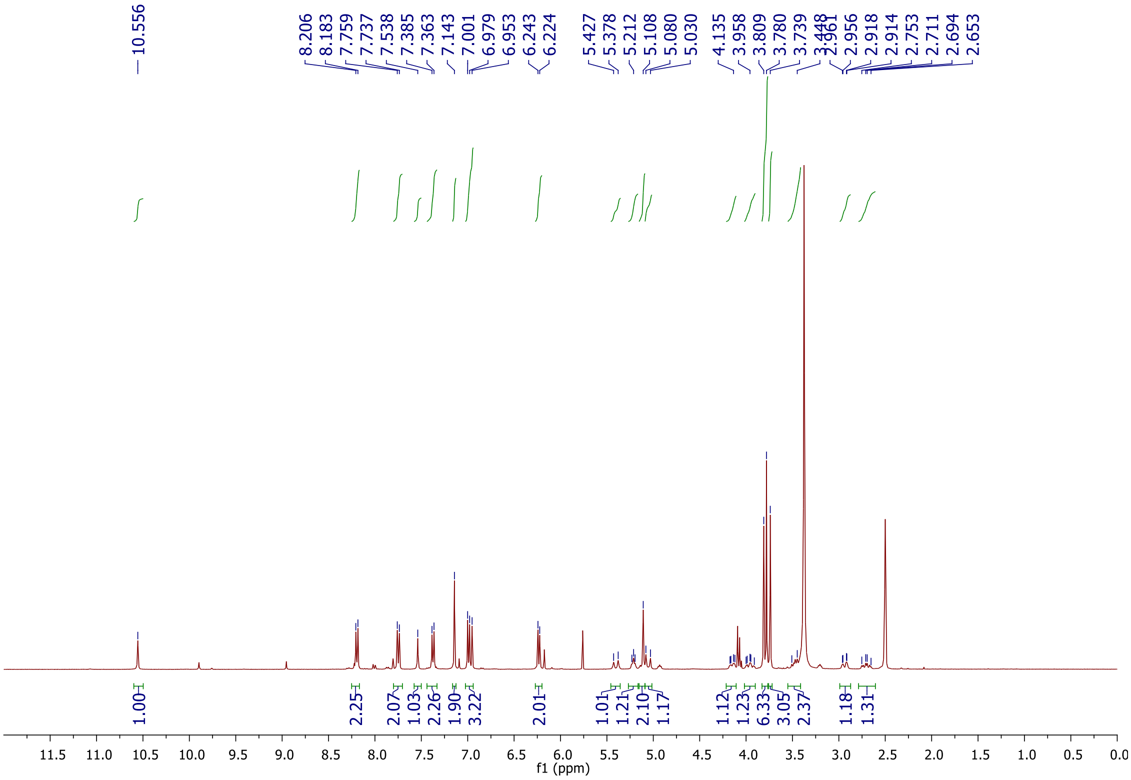

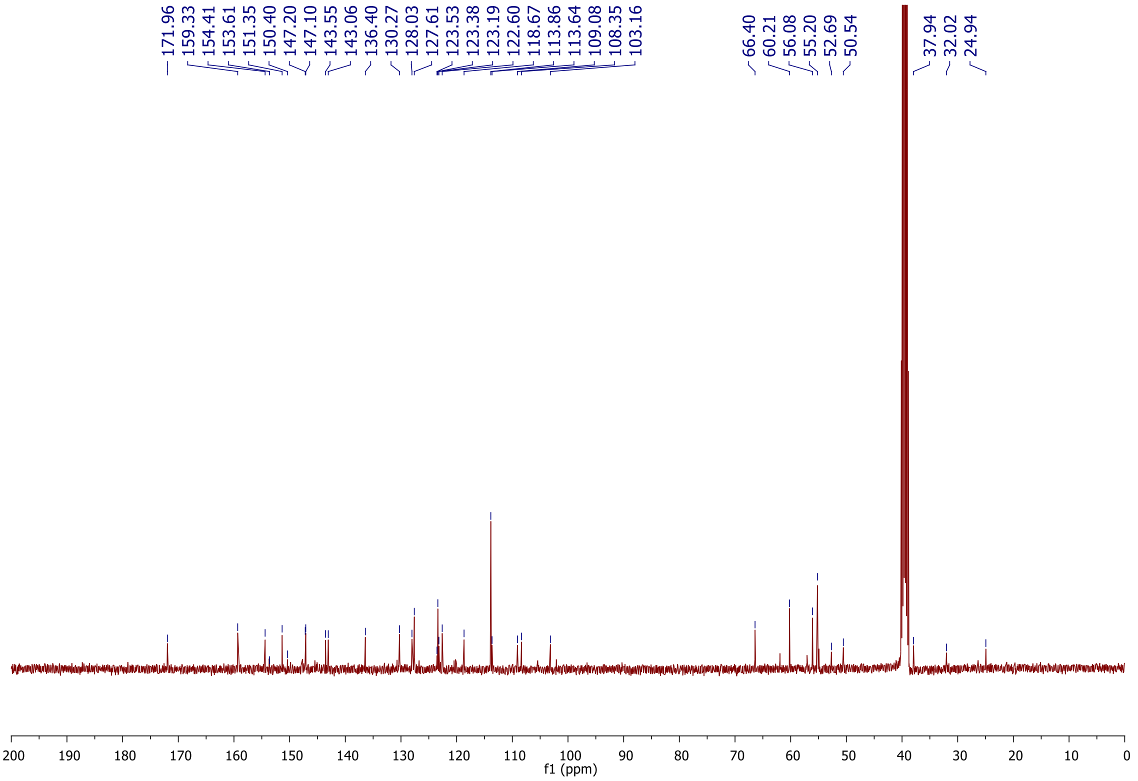

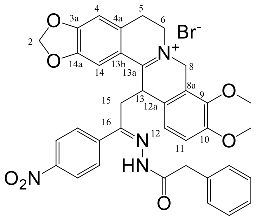

9,10-Dimethoxy-13-(2-(4-nitrophenyl)-2-(2-(2-phenylacetyl) hydrazono)ethyl)-5,6,8,13-tetrahydro-[1,3]dioxolo[4,5-*g*]isoquinolino[3,2-*a*]isoquinolin-7-ium bromide (2j)

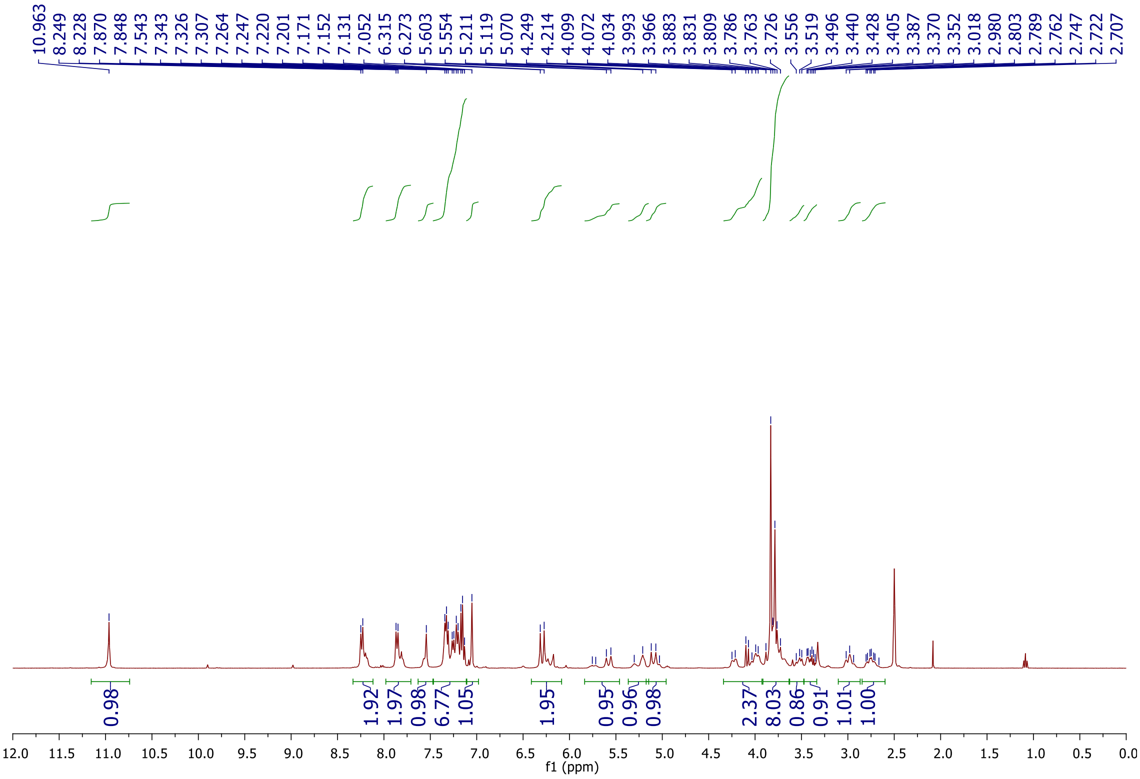

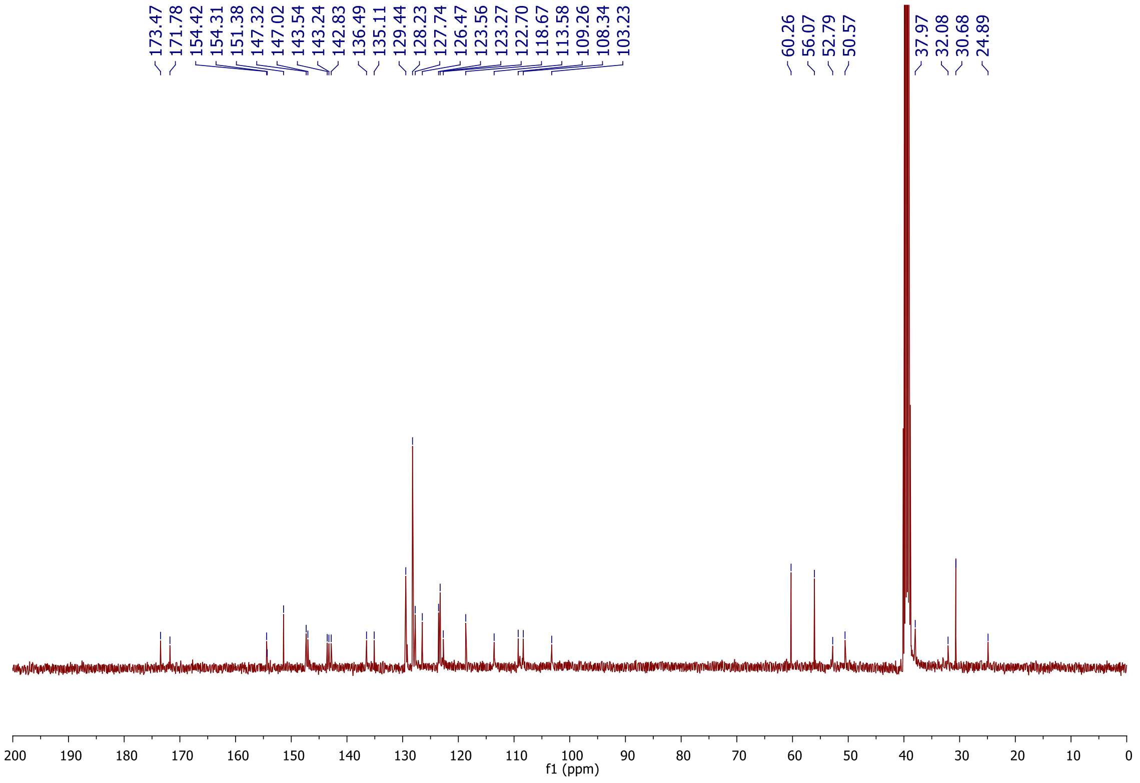

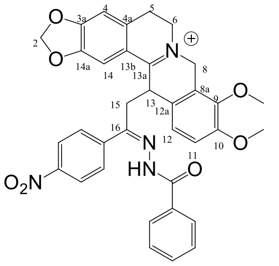

N'-(2-(9,10-Dimethoxy-6,8,13,13a-tetrahydro-5H-[1,3]dioxolo [4,5-g]isoquinolino[3,2-a]isoquinolin-13-yl)-1-(4-nitrophenyl) ethylidene)benzohydrazide (2k)

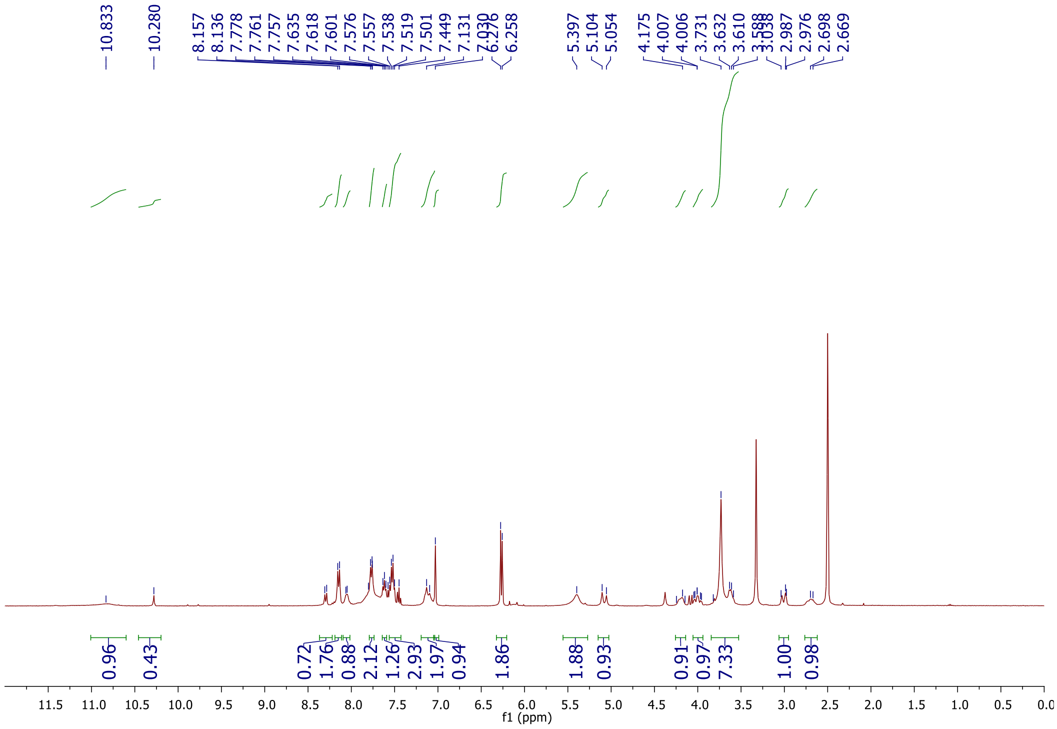

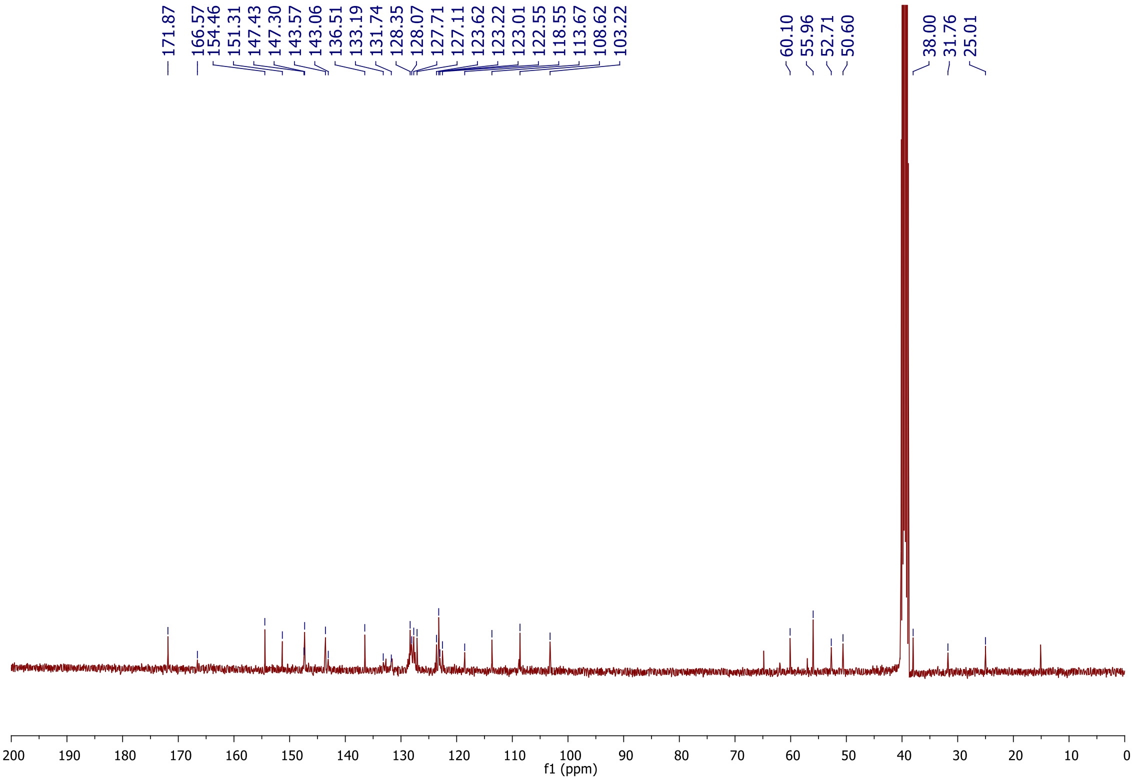

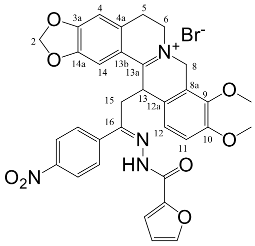

13-(2-(2-(Furan-2-carbonyl)hydrazono)-2-(4-nitrophenyl)ethyl)-9,10-dimethoxy-5,6,8,13-tetrahydro-[1,3]dioxolo[4,5-*g*]isoquinolino[3,2-*a*]isoquinolin-7-ium bromide (2l)

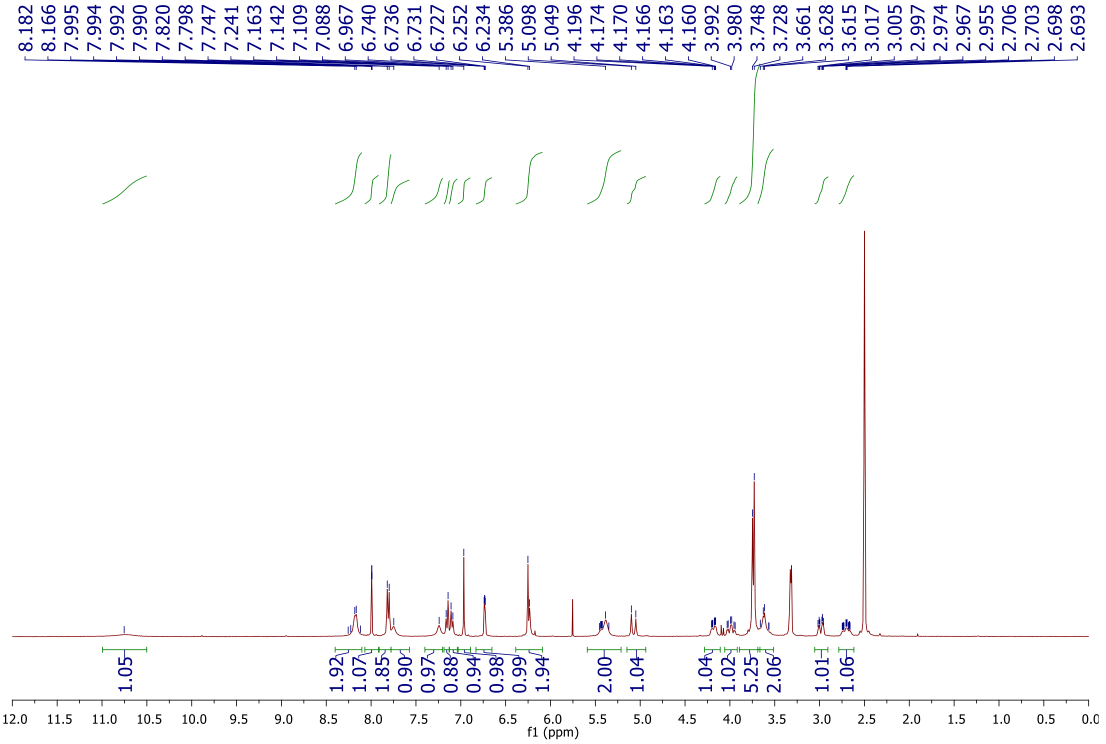

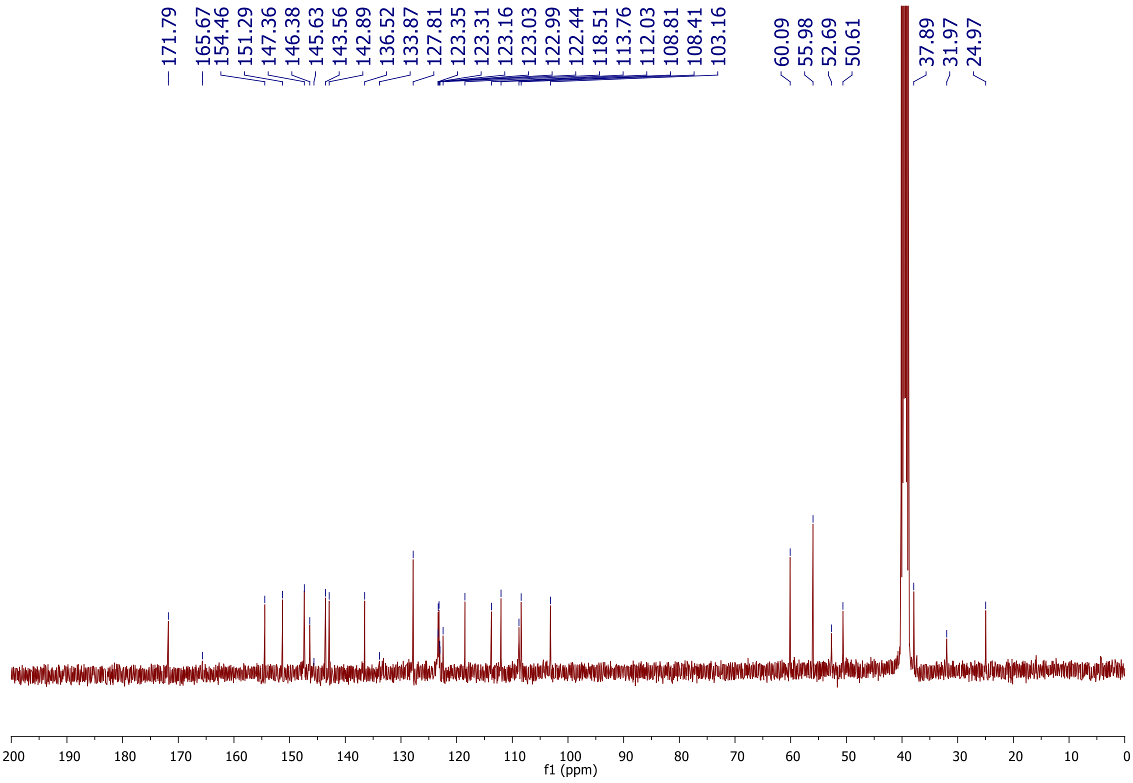

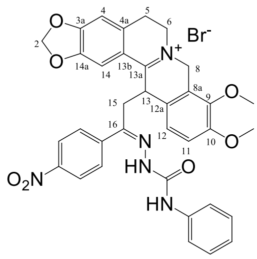

9,10-Dimethoxy-13-(2-(4-nitrophenyl)-2-(2-(phenyl carbamoyl) hydrazono)ethyl)-5,6,8,13-tetrahydro-[1,3]dioxolo[4,5-*g*]iso-quinolino[3,2-*a*]isoquinolin-7-ium bromide (2m)

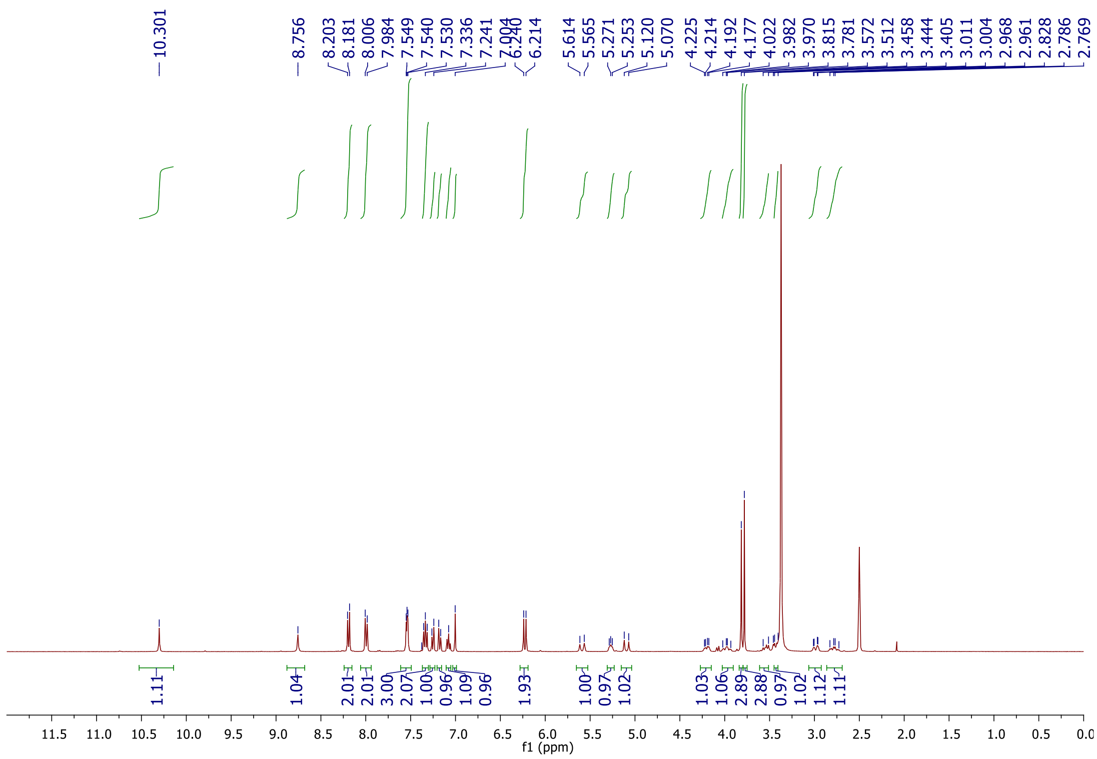

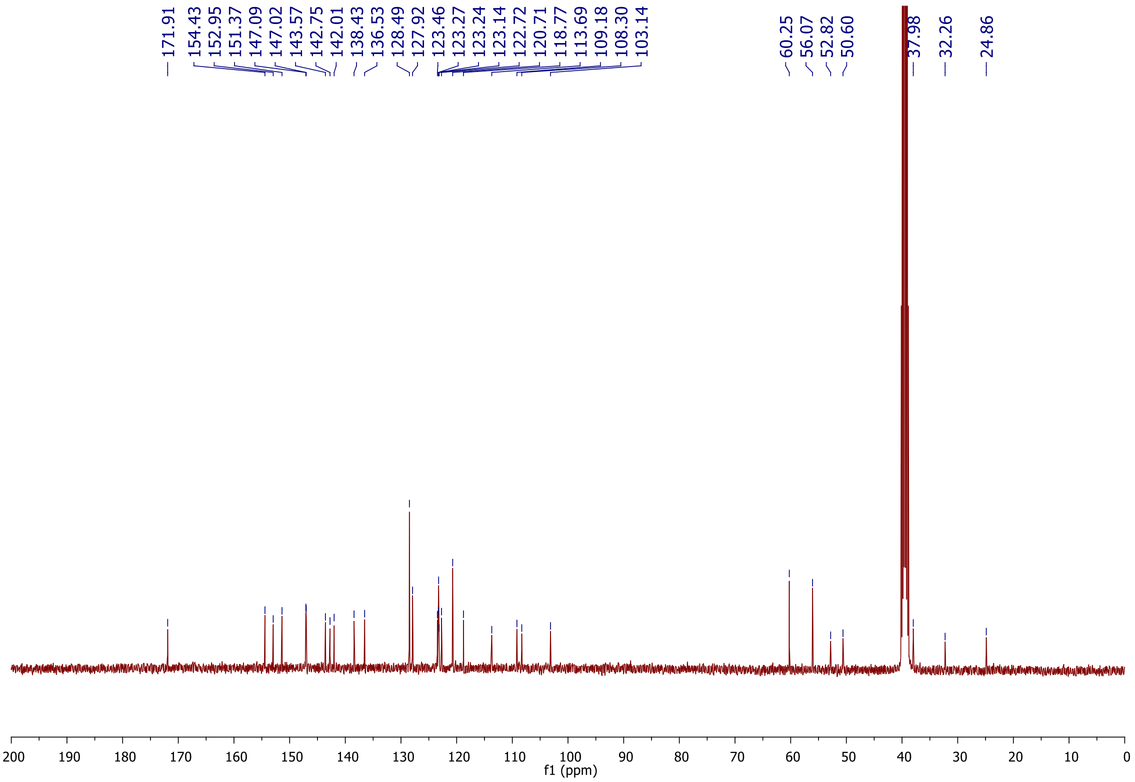

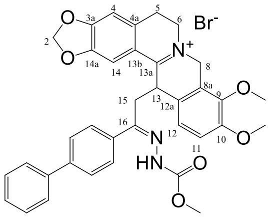

13-(2-([1,1'-Biphenyl]-4-yl)-2-(2-(methoxycarbonyl)hydrazono) ethyl)-9,10-dimethoxy-5,6,8,13-tetrahydro-[1,3]dioxolo[4,5-*g*]isoquinolino[3,2-*a*]isoquinolin-7-ium bromide (2n)

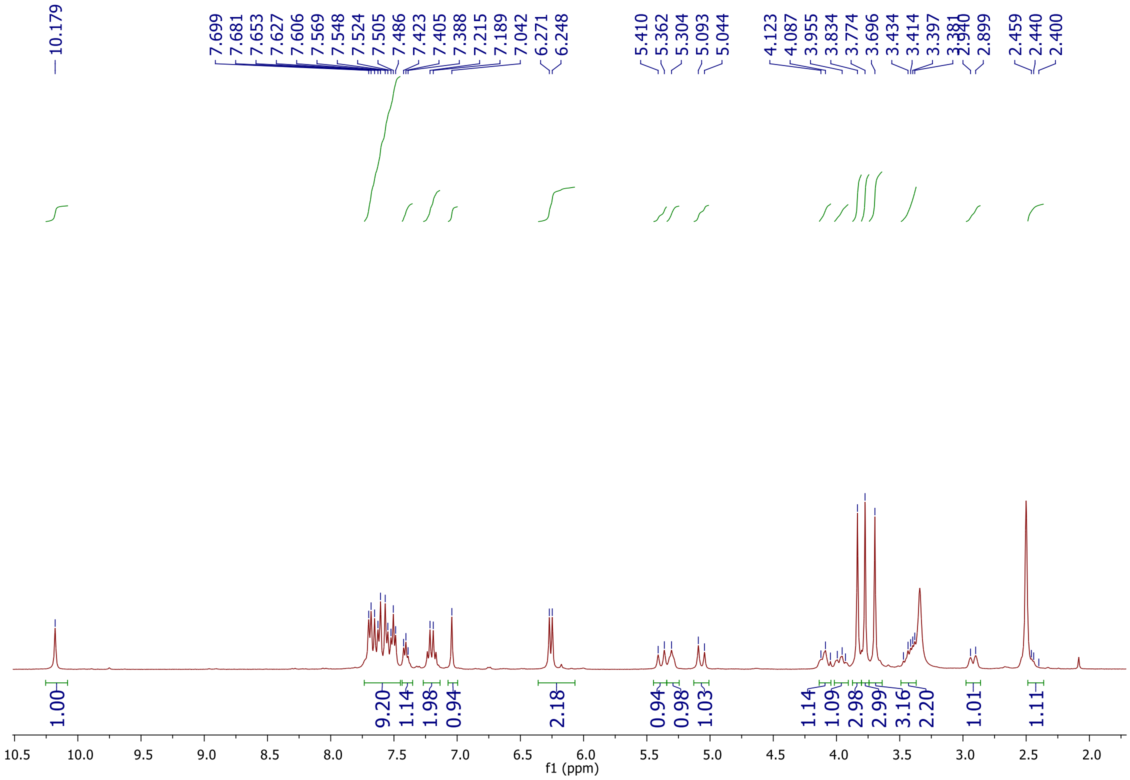

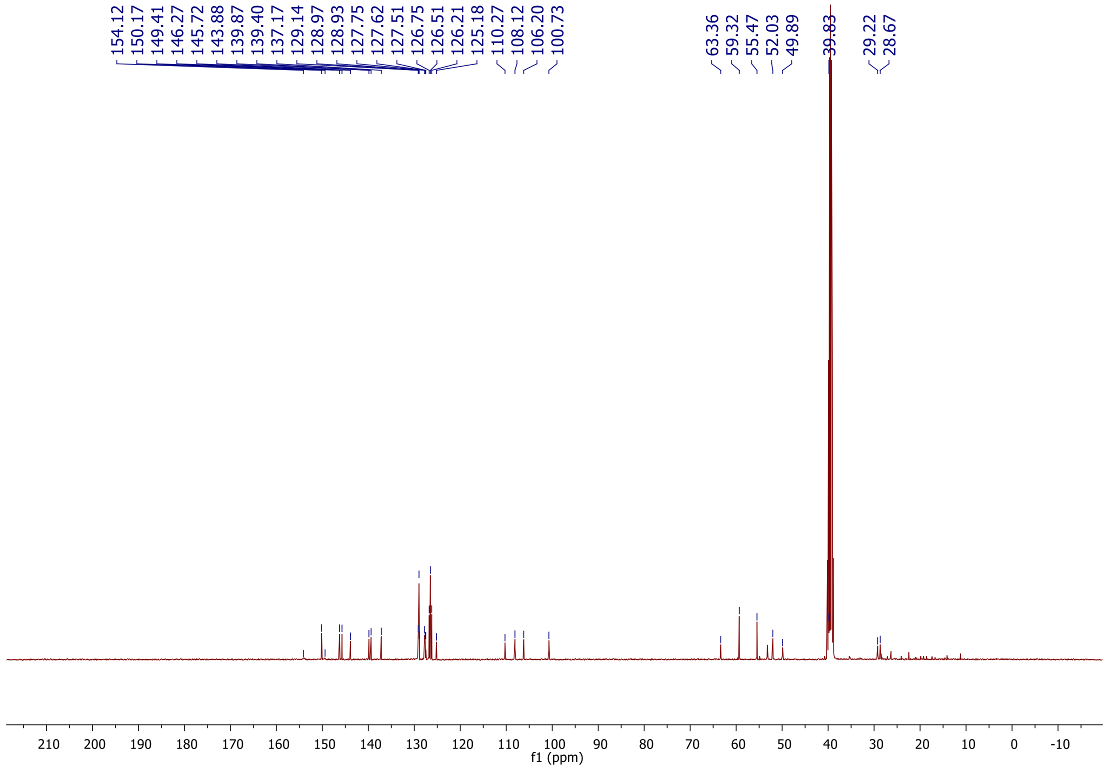

4. ^{1}H and ^{13}C NMR spectra of products 3a–n

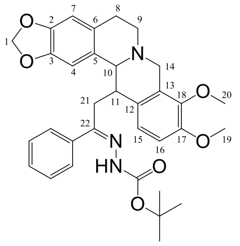

***tert*-Butyl 2-(2-(9,10-dimethoxy-6,8,13,13a-tetrahydro-5*H*-[1,3]dioxolo[4,5-*g*]isoquinolino[3,2-*a*]isoquinolin-13-yl)-1-phenylethylidene)hydrazinecarboxylate (3a)**

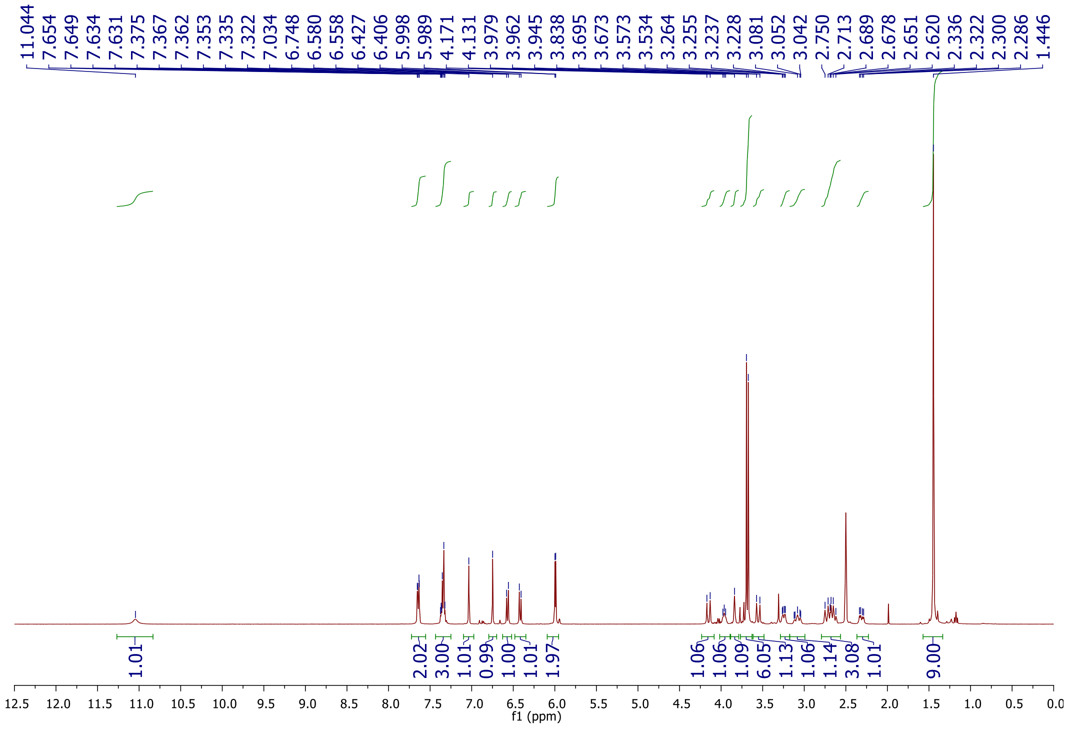

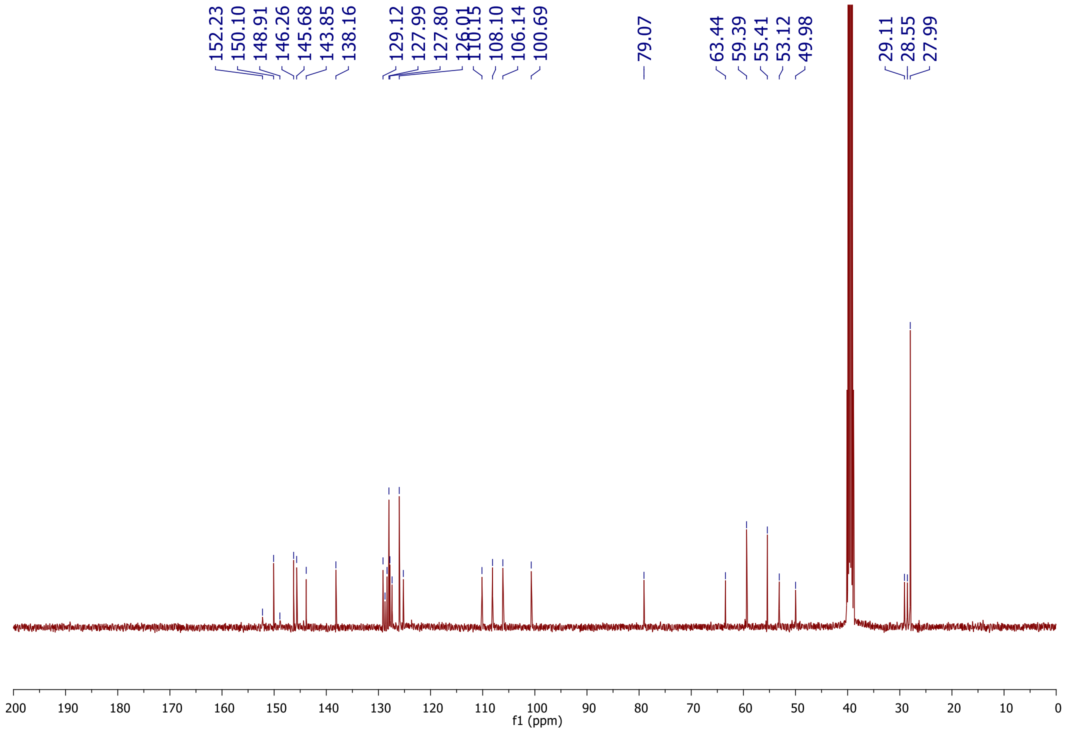

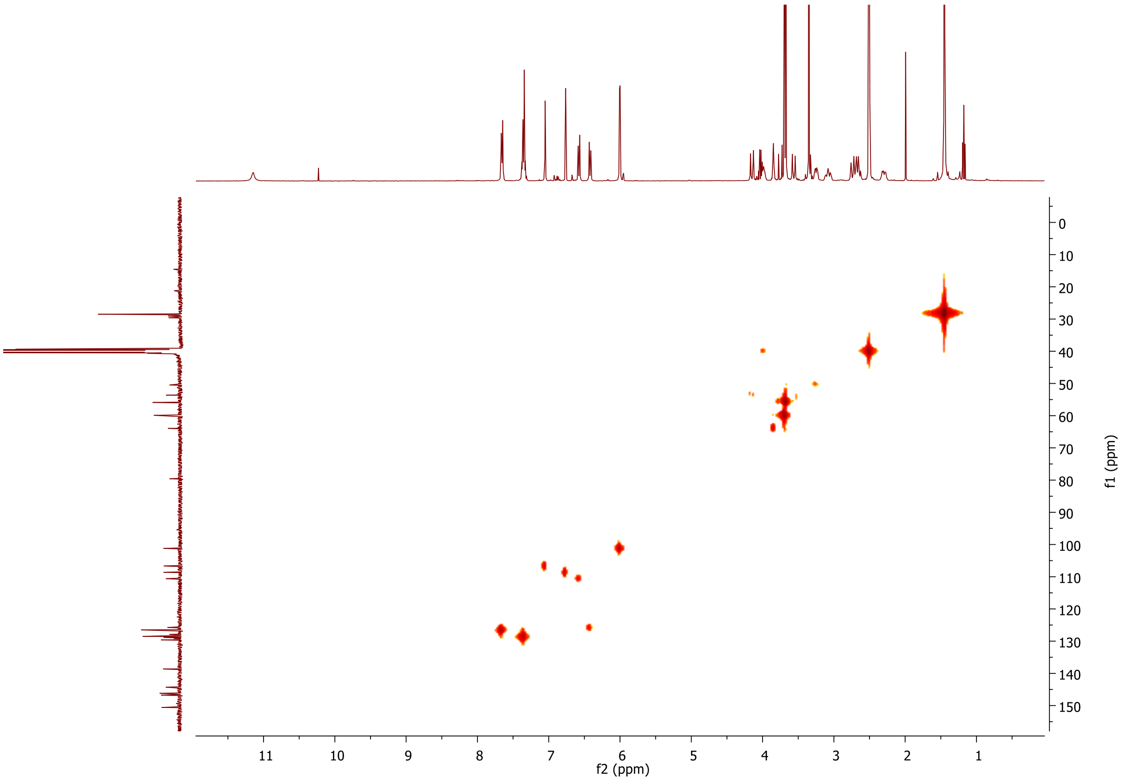

11
10
9
8
7
6
5
4
3
2
1
f2 (ppm)
0
10
20
30
40
50
60
70
80
90
100
110
120
130
140
150
f1 (ppm)

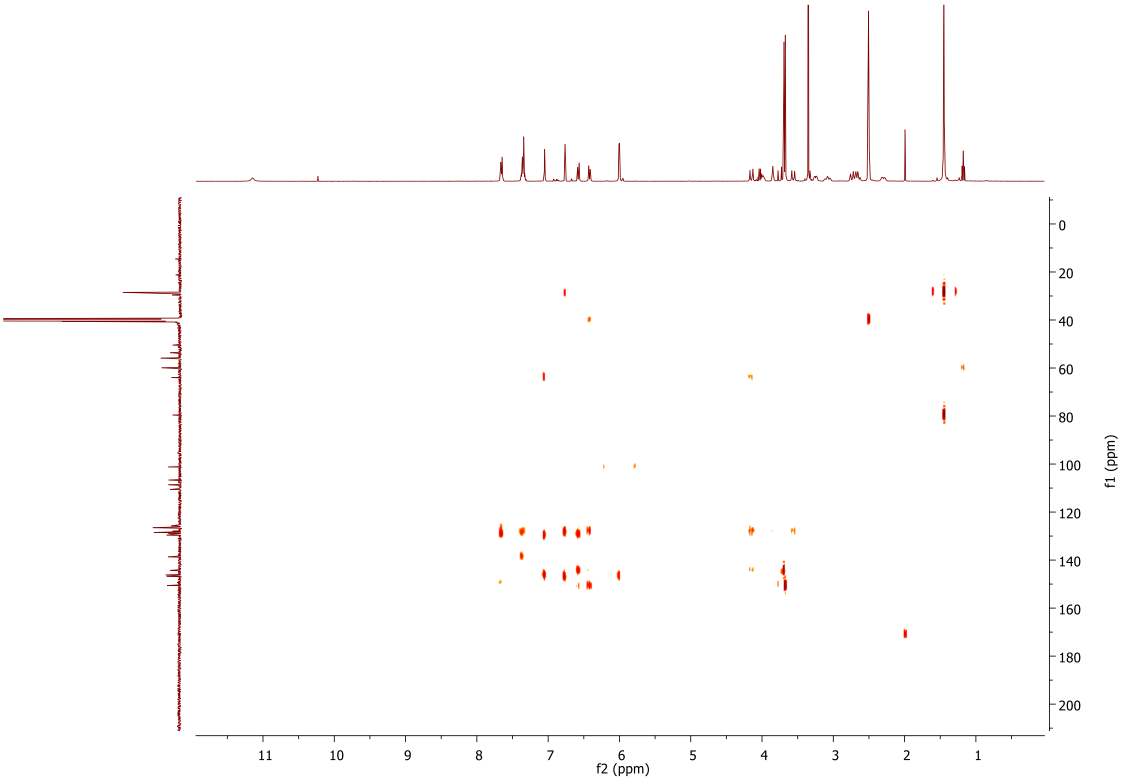

11
10
9
8
7
6
5
4
3
2
1
f2 (ppm)
0
20
40
60
80
100
120
140
160
180
200
f1 (ppm)

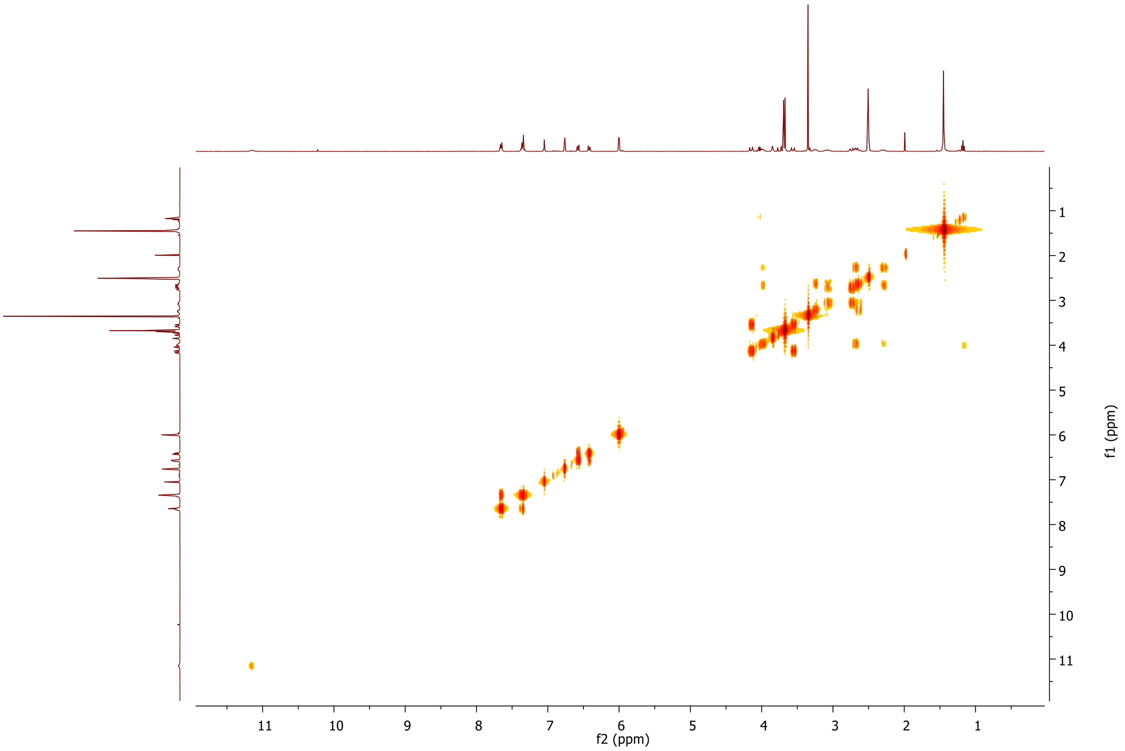
f2 (ppm)
f1 (ppm)

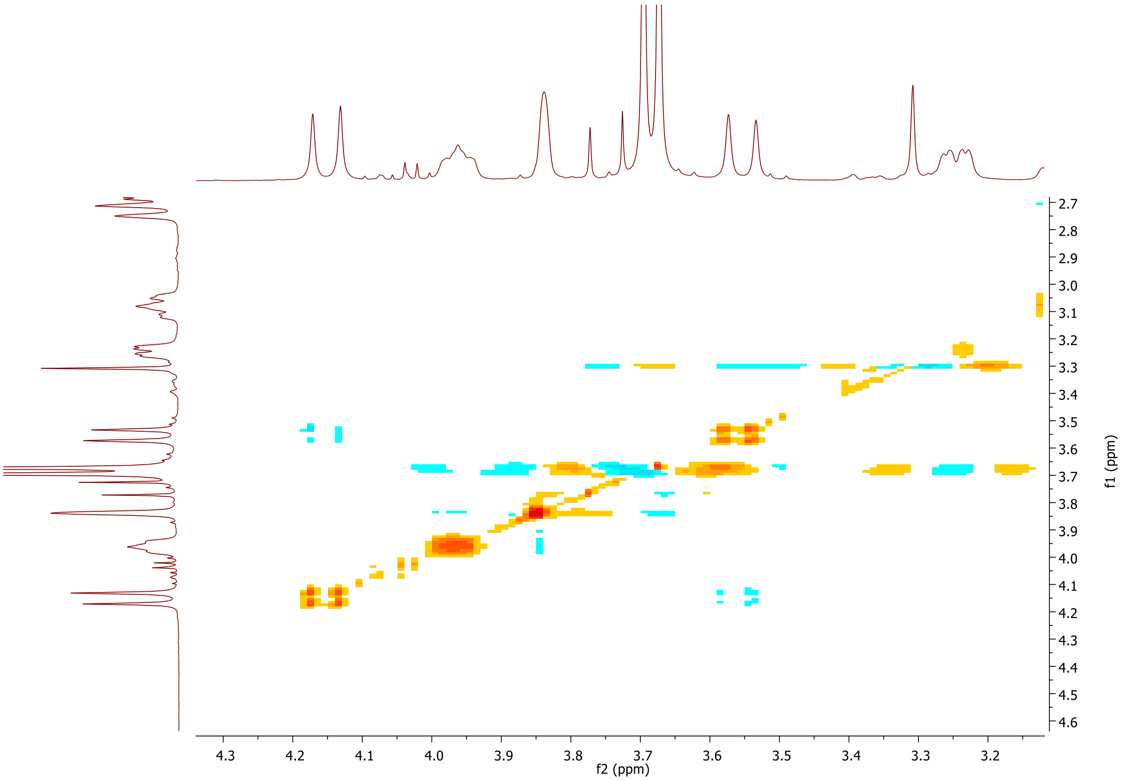
f2 (ppm)
f1 (ppm)

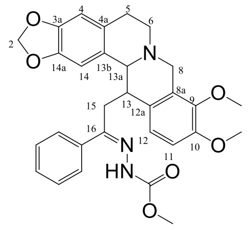

Methyl-2-(2-(9,10-dimethoxy-6,8,13,13a-tetrahydro-5*H*-[1,3]dioxolo[4,5-*g*]isoquinolino[3,2-*a*]isoquinolin-13-yl)-1-phenylethylidene)hydrazinecarboxylate (3b)

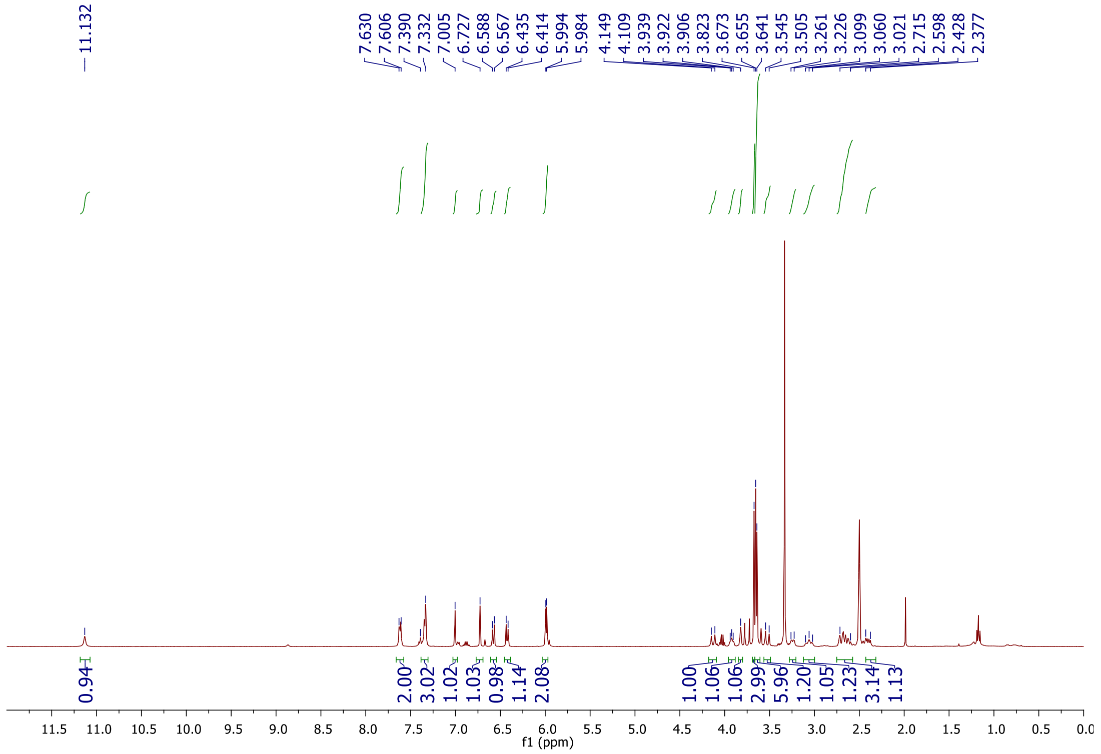

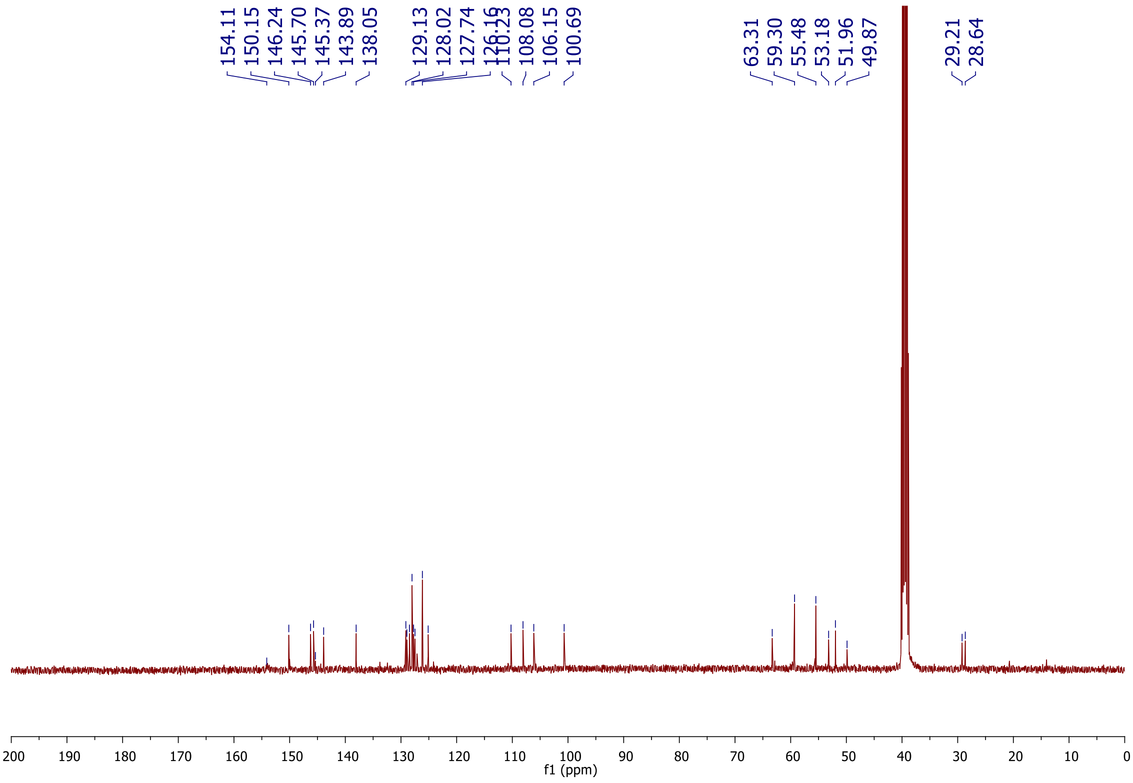

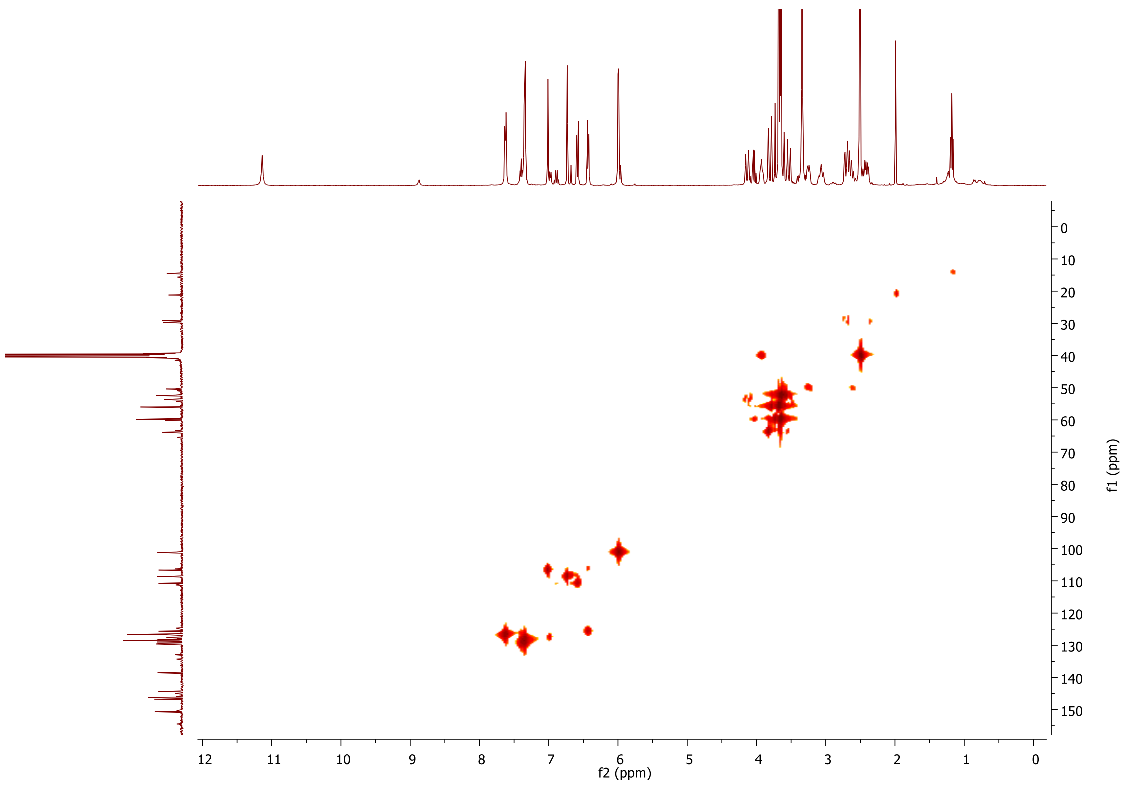
f2 (ppm)
f1 (ppm)

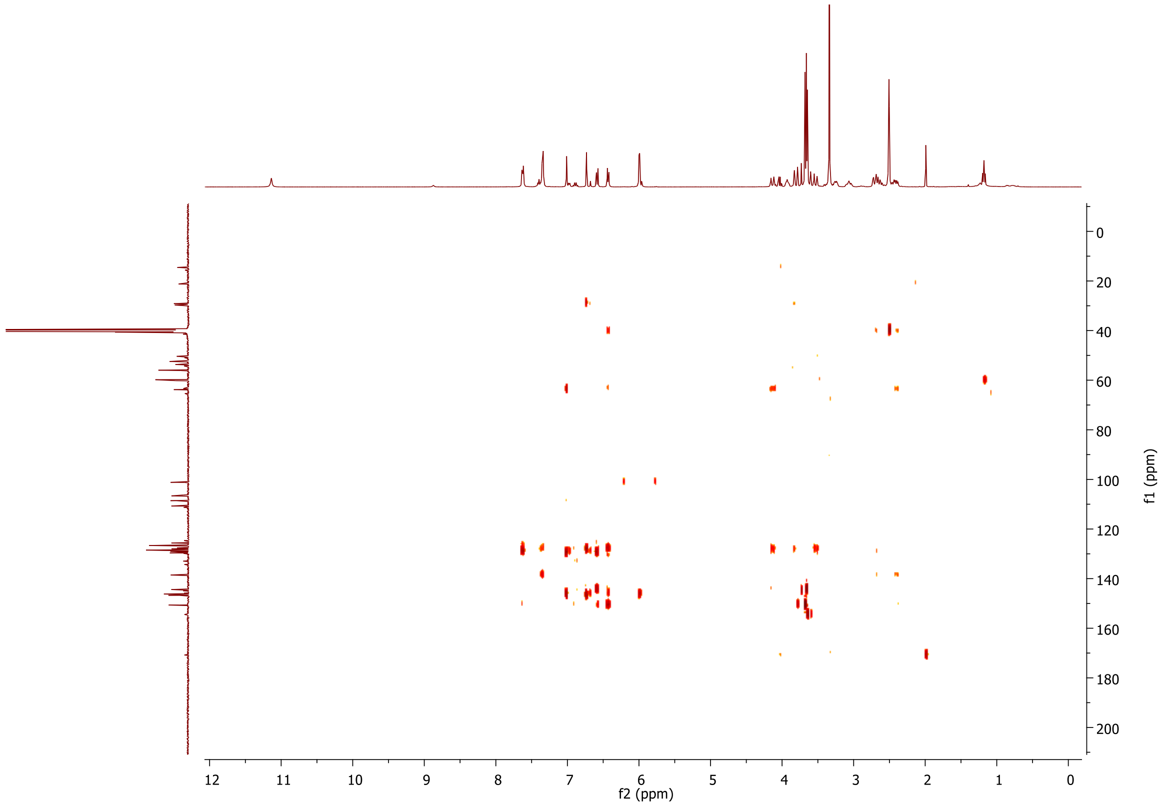
f2 (ppm)
f1 (ppm)

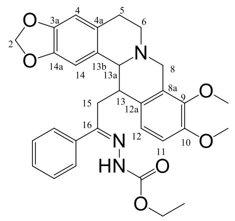

Ethyl 2-(2-(9,10-dimethoxy-6,8,13,13a-tetrahydro-5*H*-[1,3]dioxolo[4,5-*g*]isoquinolino[3,2-*a*]isoquinolin-13-yl)-1-phenylethylidene)hydrazinecarboxylate (3c)

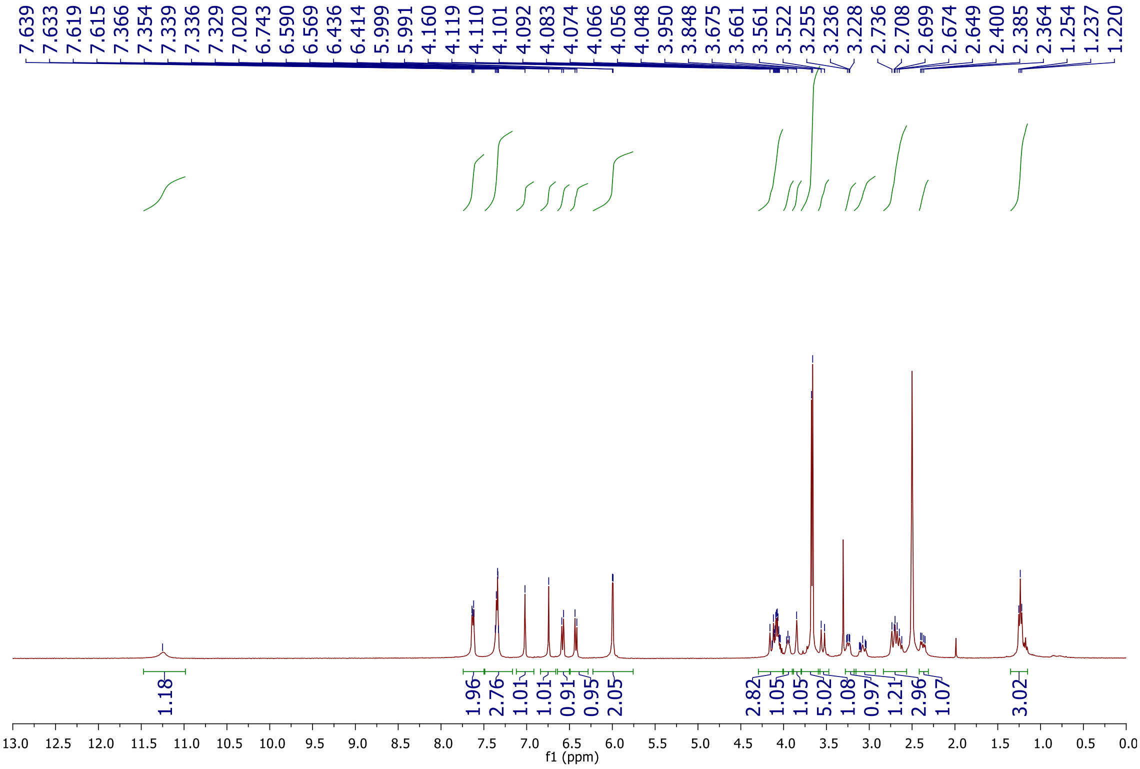

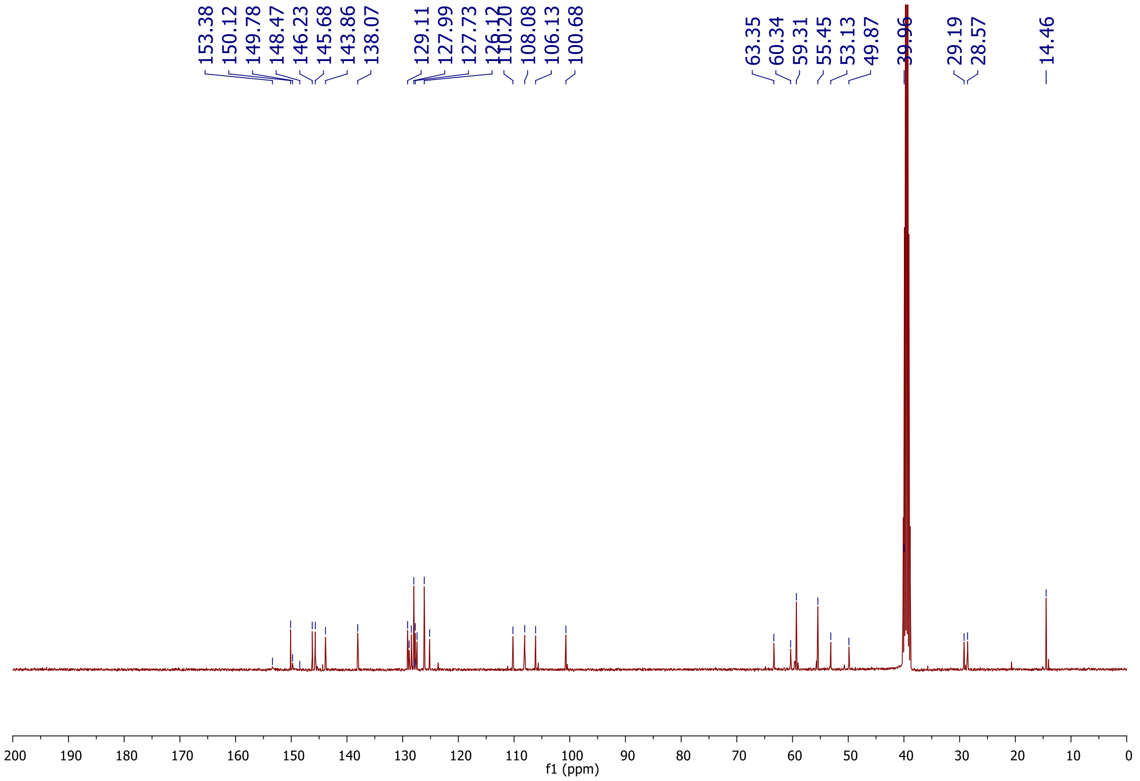

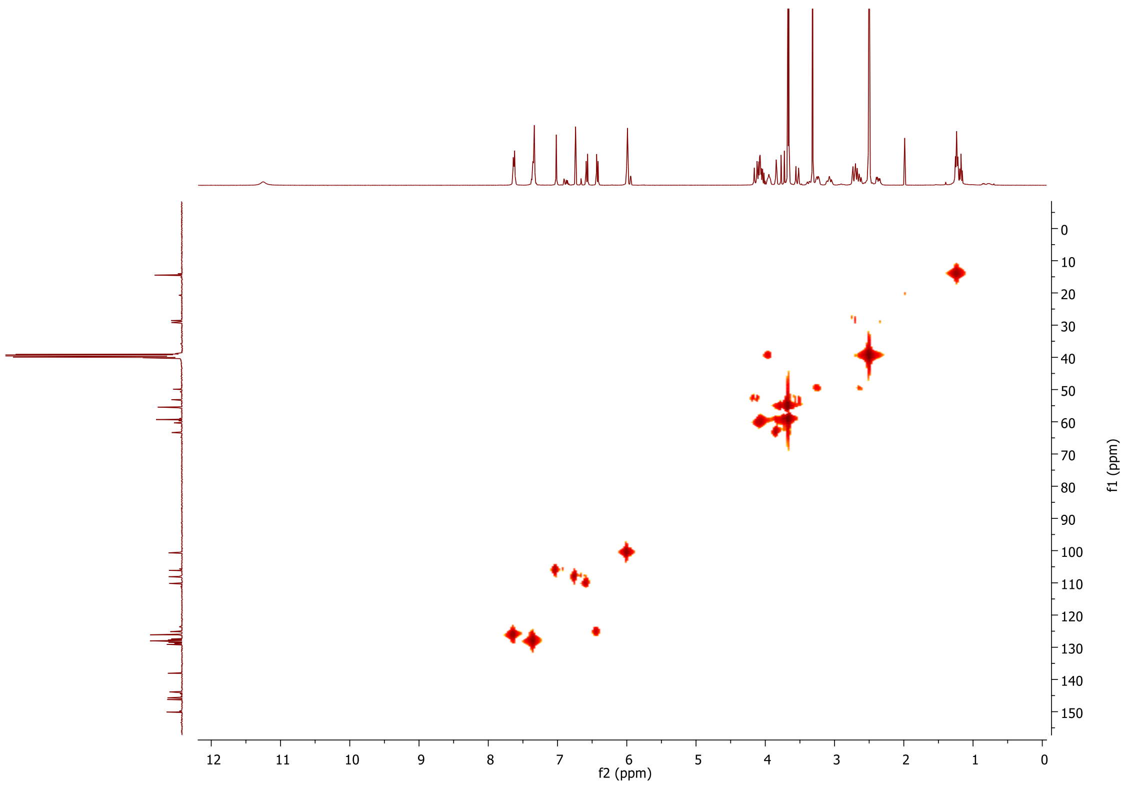
f2 (ppm)
f1 (ppm)

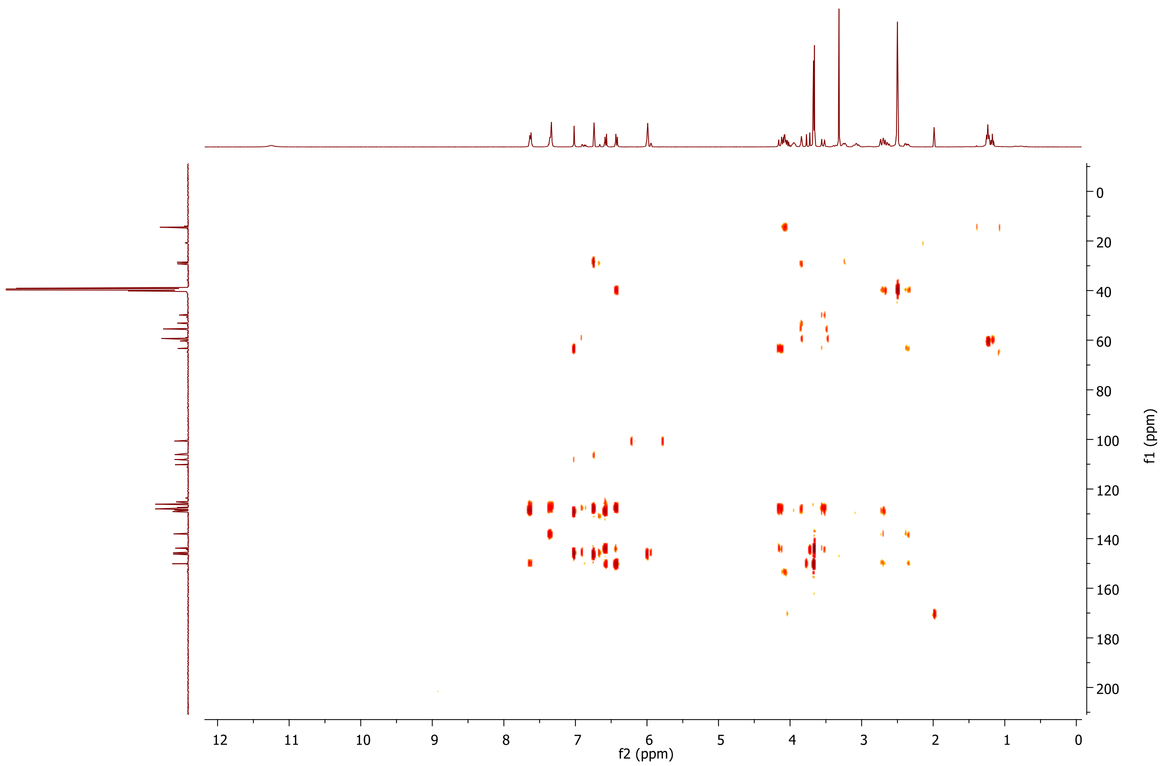
f2 (ppm)
f1 (ppm)

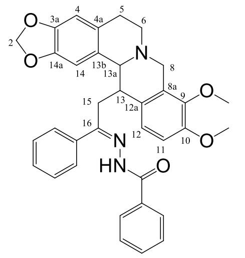

***N'*-(2-(9,10-dimethoxy-6,8,13,13a-tetrahydro-5*H*-[1,3]dioxolo[4,5-*g*]isoquinolino[3,2-*a*]isoquinolin-13-yl)-1-phenylethylidene) benzohydrazide (3d)**

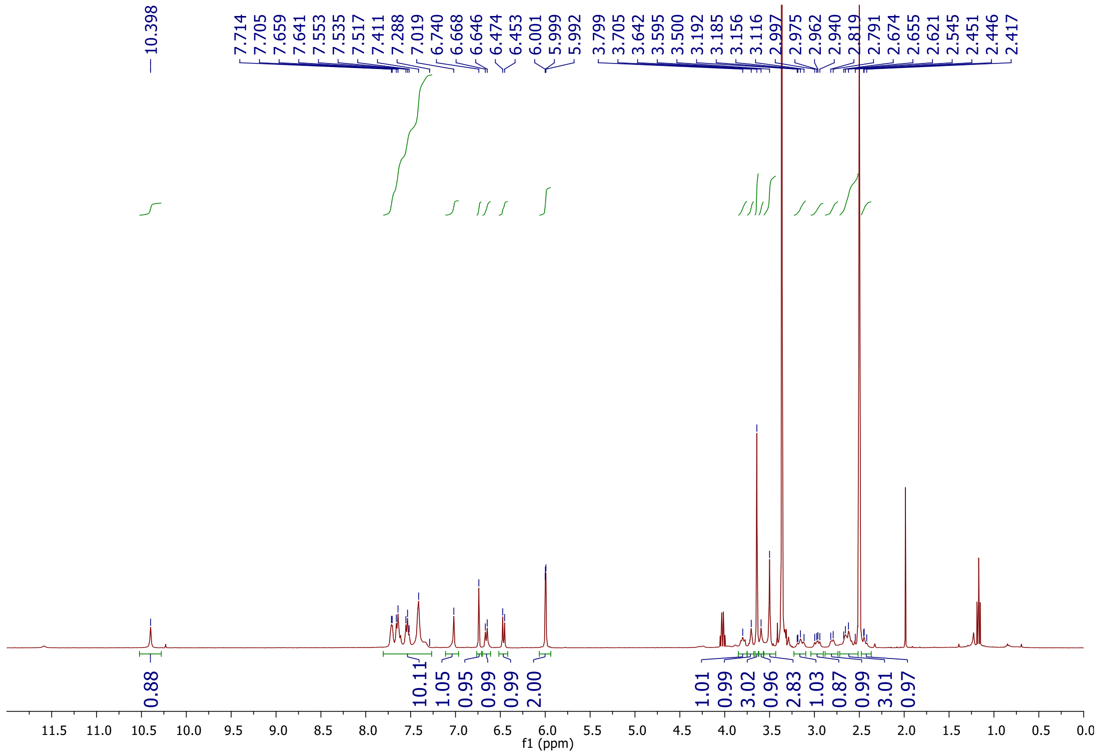

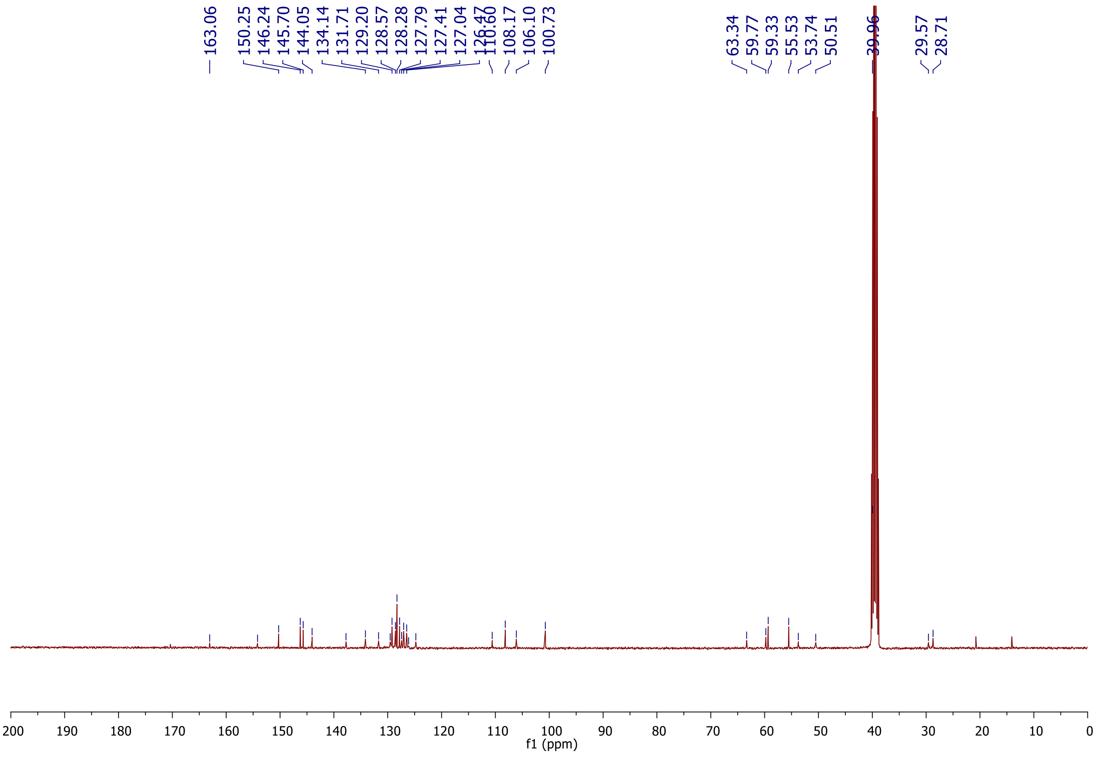

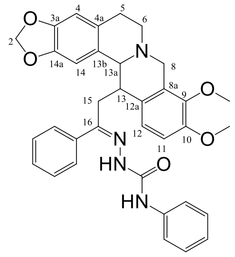

2-(2-(9,10-Dimethoxy-6,8,13,13a-tetrahydro-5*H*-[1,3]dioxolo[4,5-*g*]isoquinolino[3,2-*a*]isoquinolin-13-yl)-1-phenylethylidene)-*N*-phenylhydrazinecarboxyamide (3e)

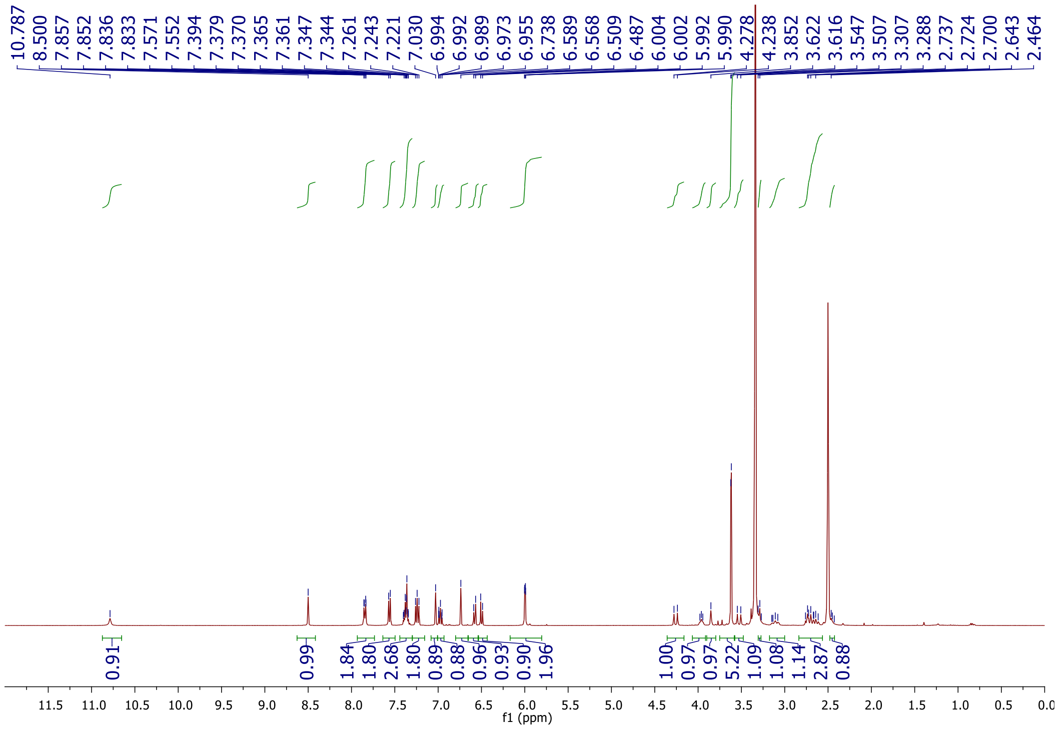

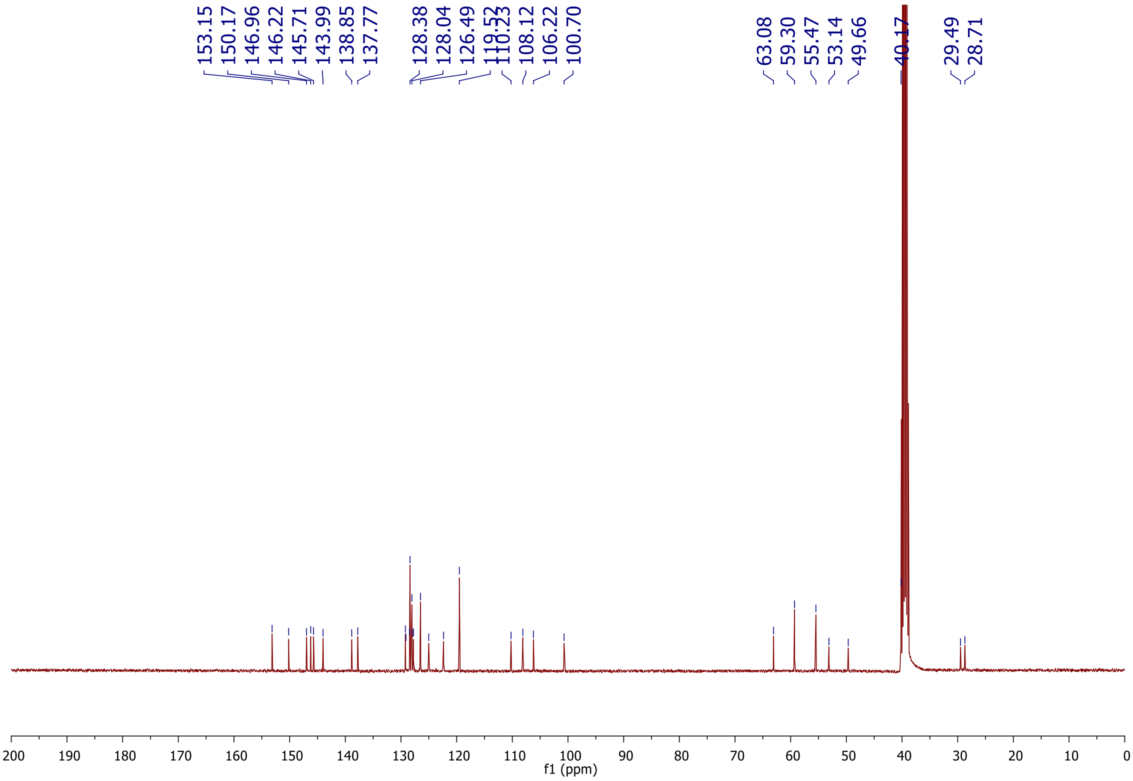

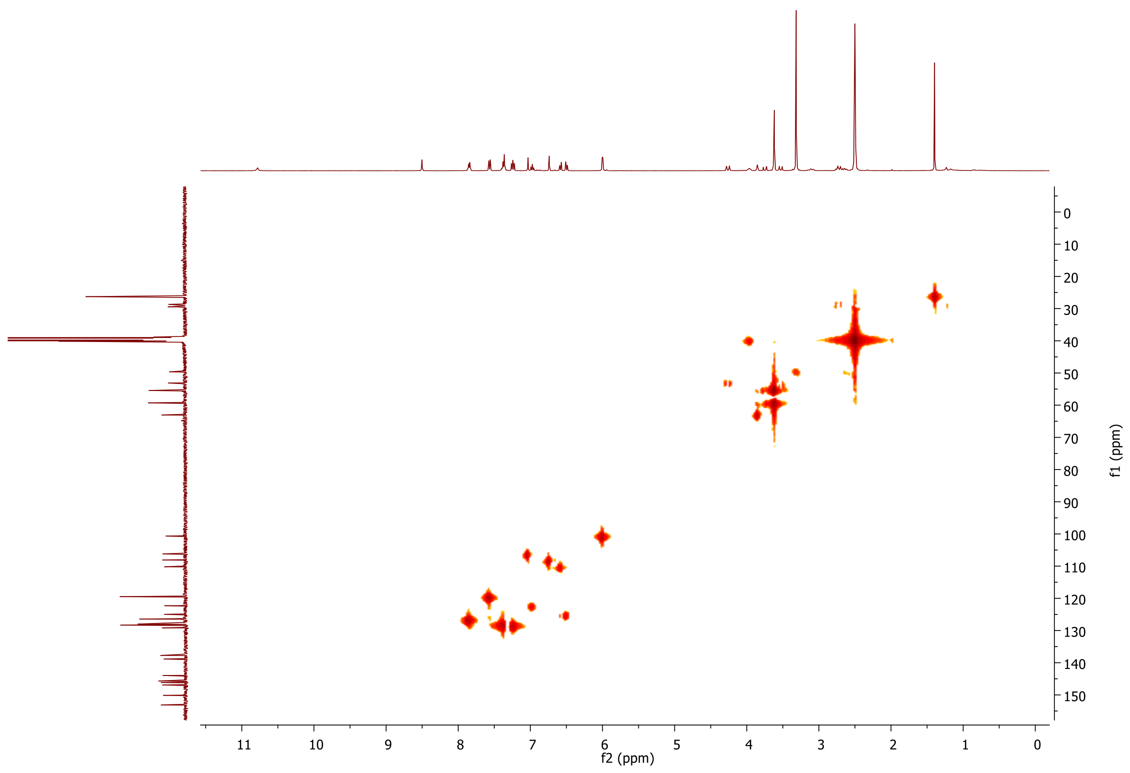

f2 (ppm)
f1 (ppm)

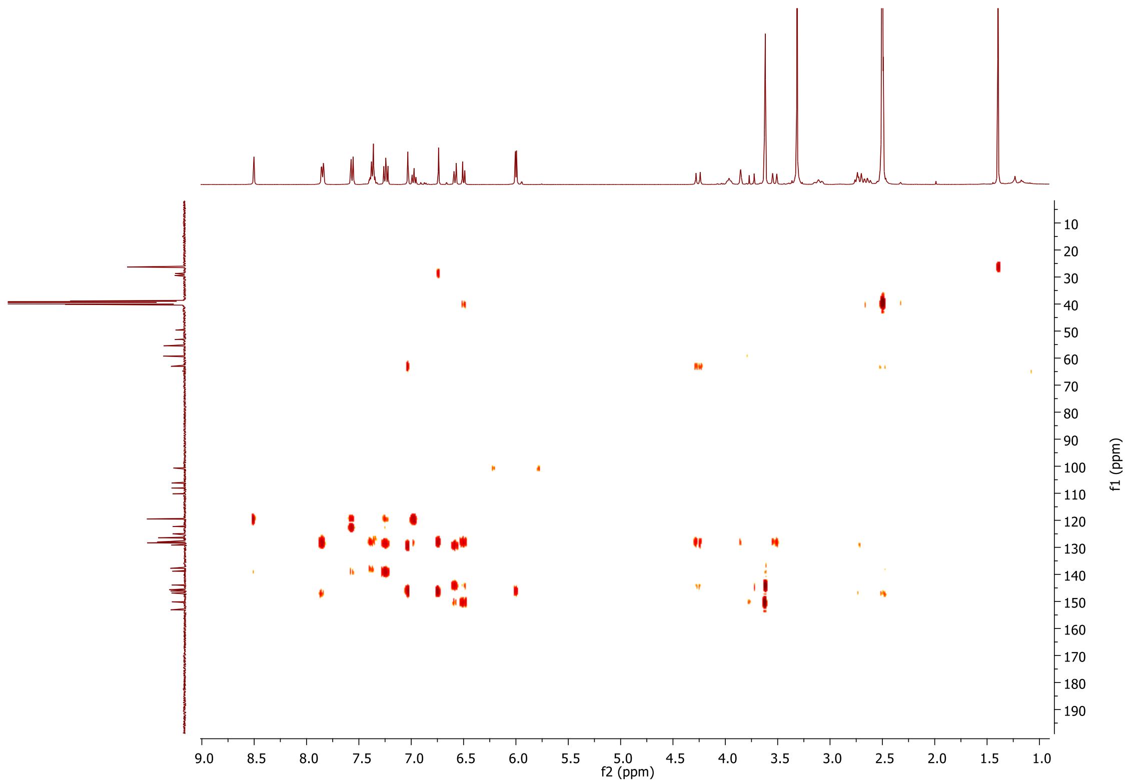

f2 (ppm)
f1 (ppm)

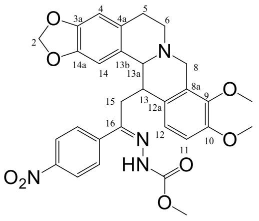

Methyl-2-(2-(9,10-dimethoxy-6,8,13,13a-tetrahydro-5*H*-[1,3]dioxolo[4,5-*g*]isoquinolino[3,2-*a*]isoquinolin-13-yl)-1-(4-nitrophenyl)ethylidene)hydrazinecarboxylate (3f)

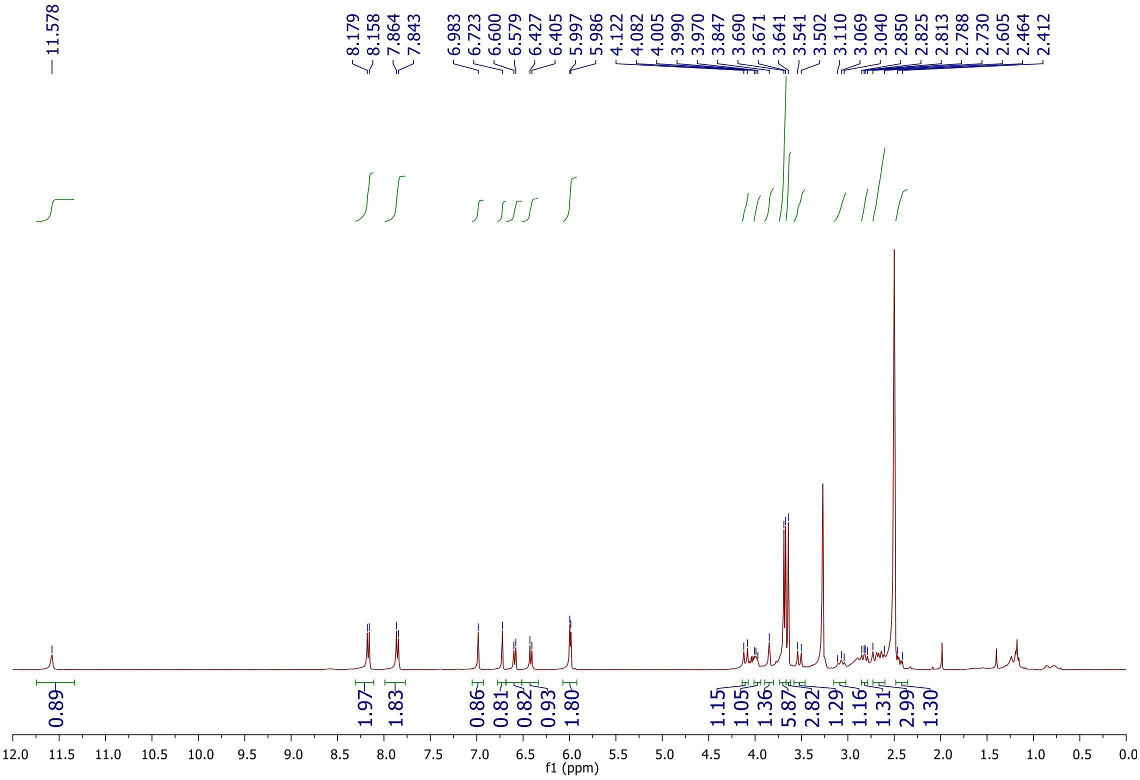

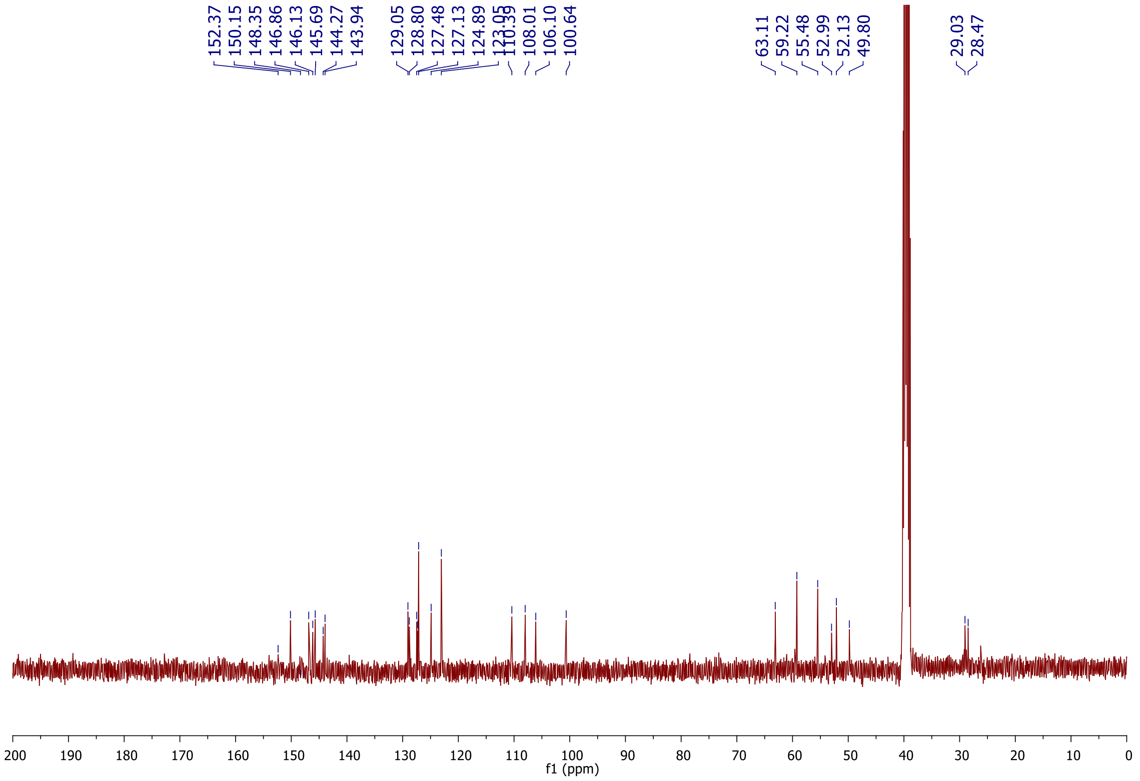

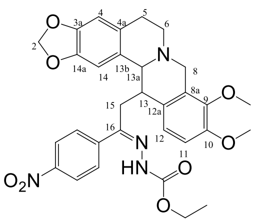

Ethyl-2-(2-(9,10-dimethoxy-6,8,13,13a-tetrahydro-5*H*-[1,3]dioxolo[4,5-*g*]isoquinolino[3,2-*a*]isoquinolin-13-yl)-1-(4-nitrophenyl)ethylidene)hydrazinecarboxylate (3g)

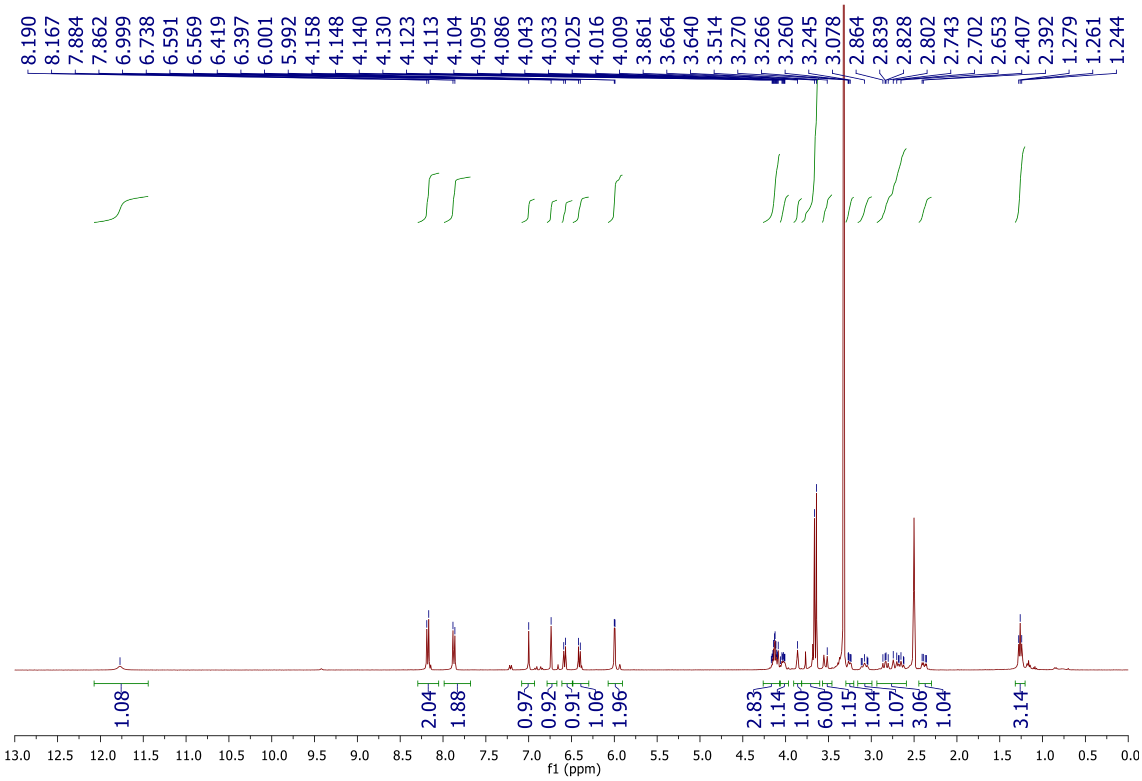

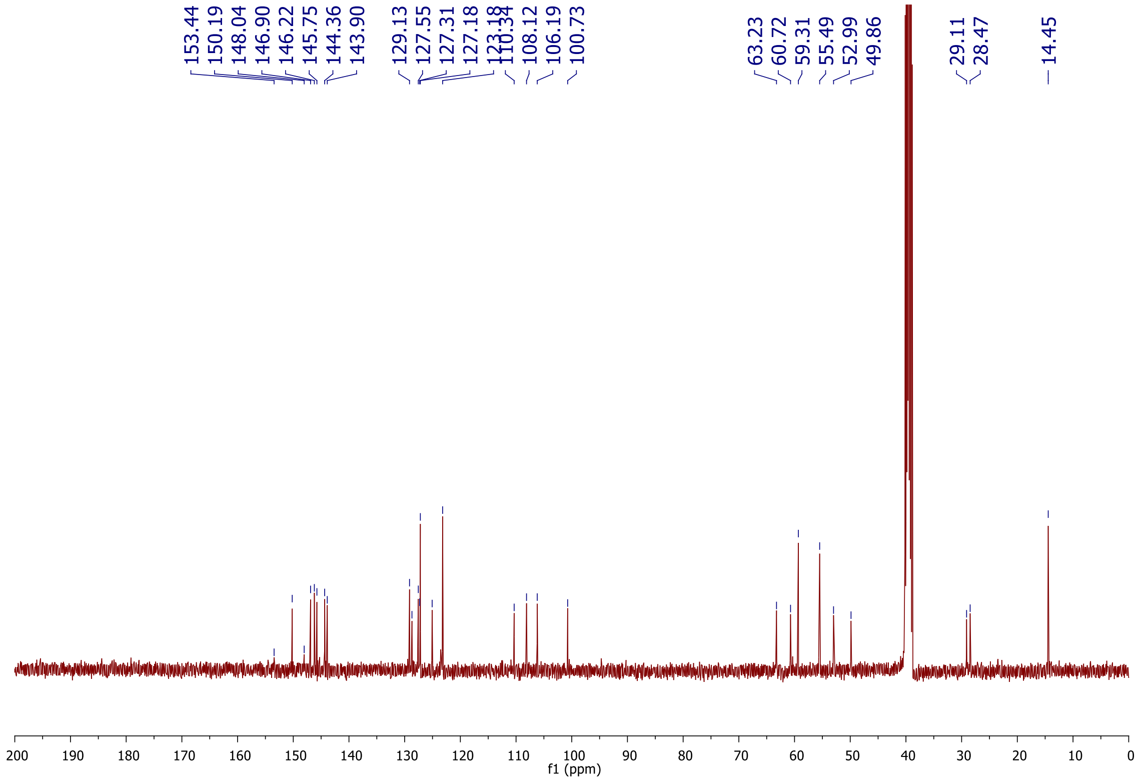

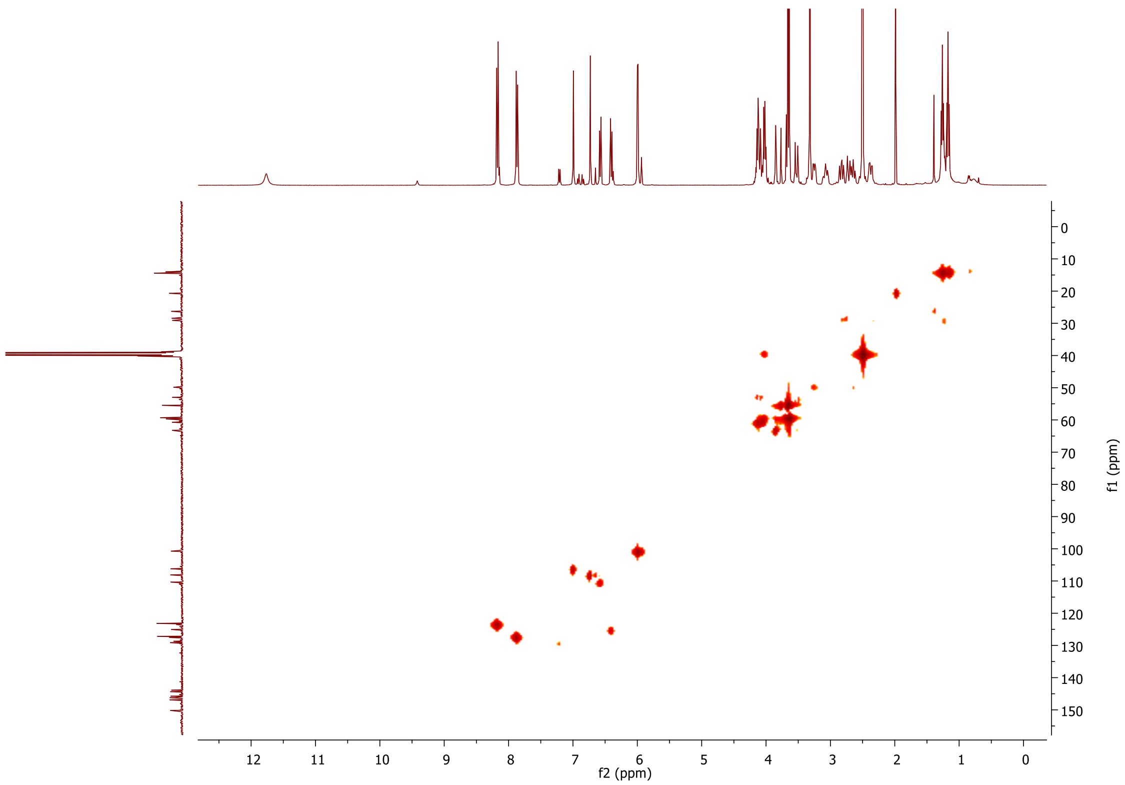
f1 (ppm)
f2 (ppm)

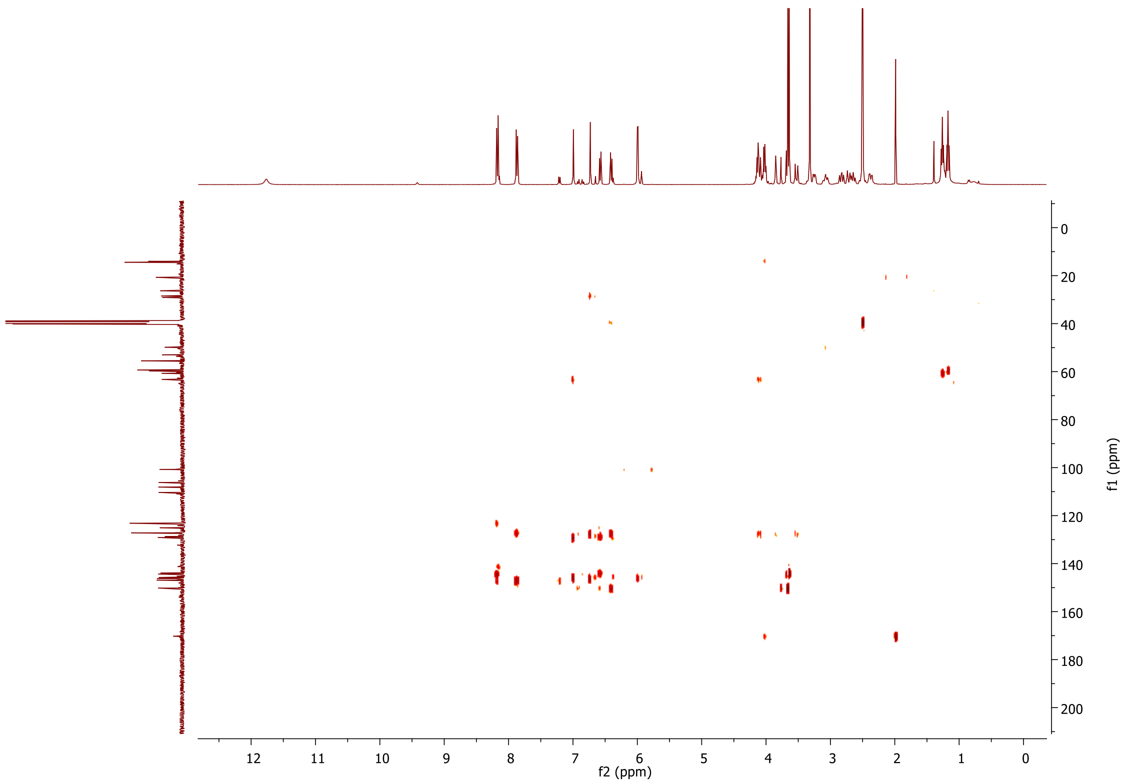
f1 (ppm)
f2 (ppm)

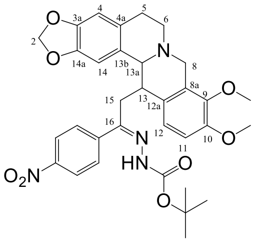

tert-Butyl-2-(2-(9,10-dimethoxy-6,8,13,13a-tetrahydro-5_H_-[1,3]dioxolo[4,5-_g_]isoquinolino[3,2-_a_]isoquinolin-13-yl)-1-(4-nitrophenyl)ethylidene)hydrazinecarboxylate (3h)

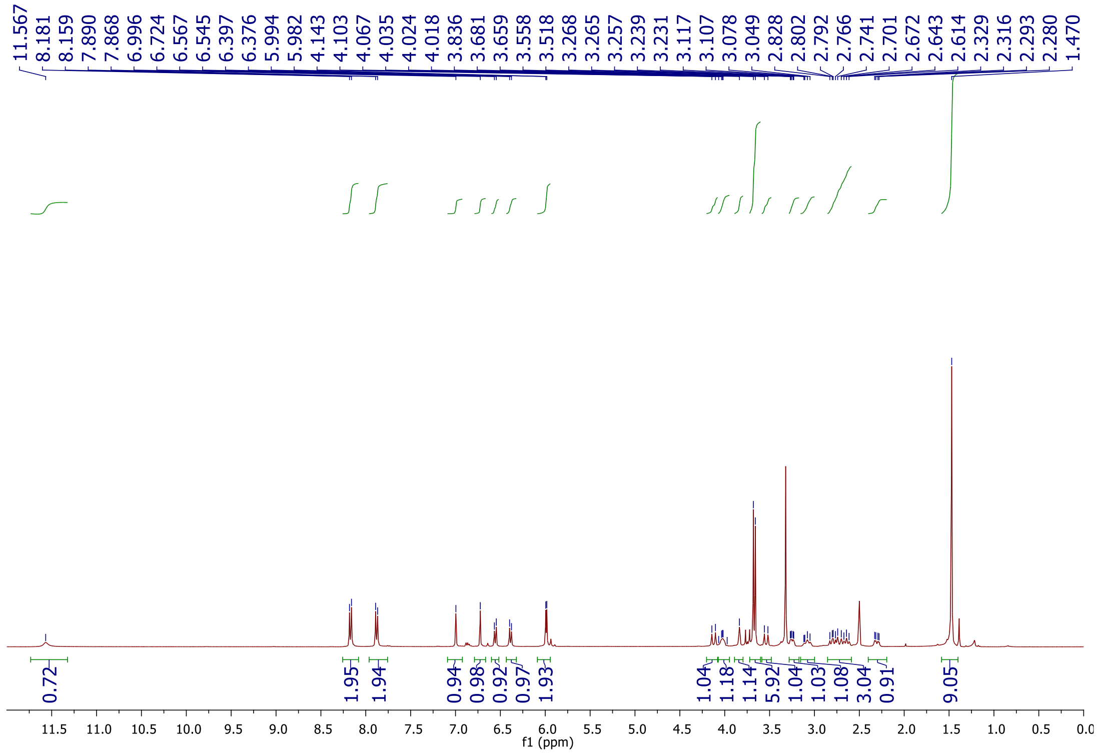

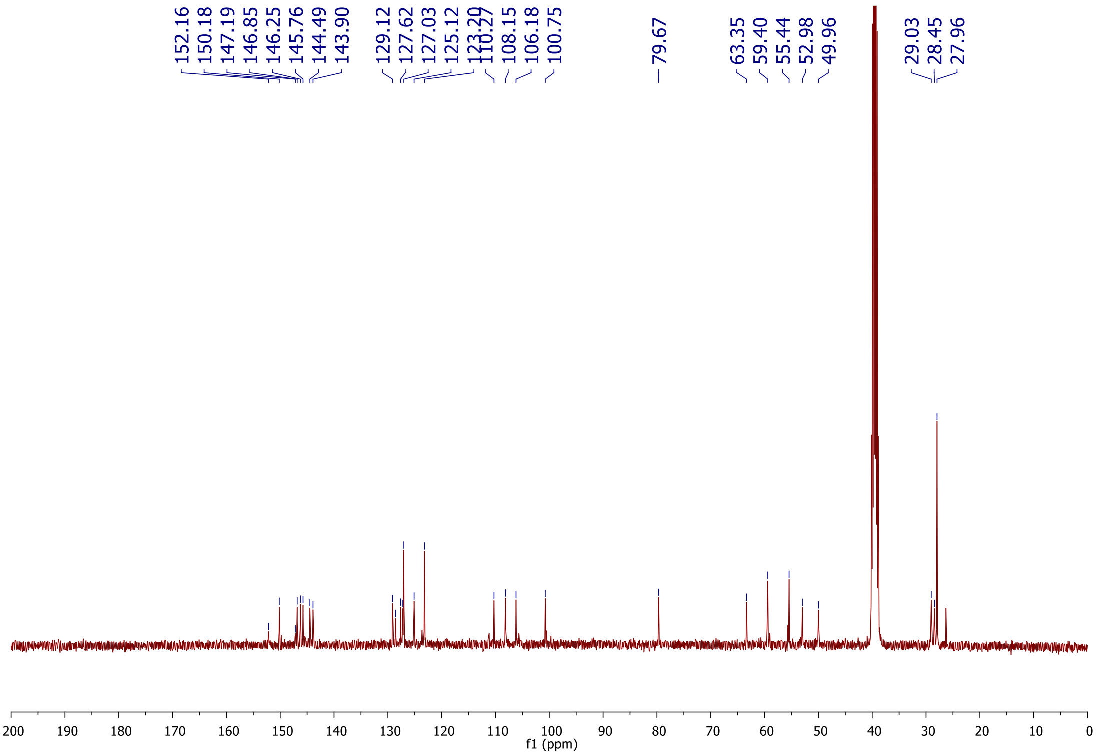

4-Methoxybenzyl 2-(2-(9,10-dimethoxy-6,8,13,13a-tetrahydro-5*H*-[1,3]dioxolo[4,5-*g*]isoquinolino[3,2-*a*]isoquinolin-13-yl)-1-(4-nitrophenyl)ethylidene)hydrazinecarboxylate (3i)

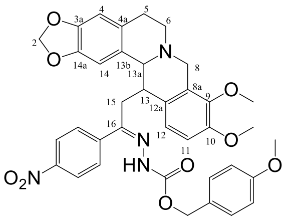

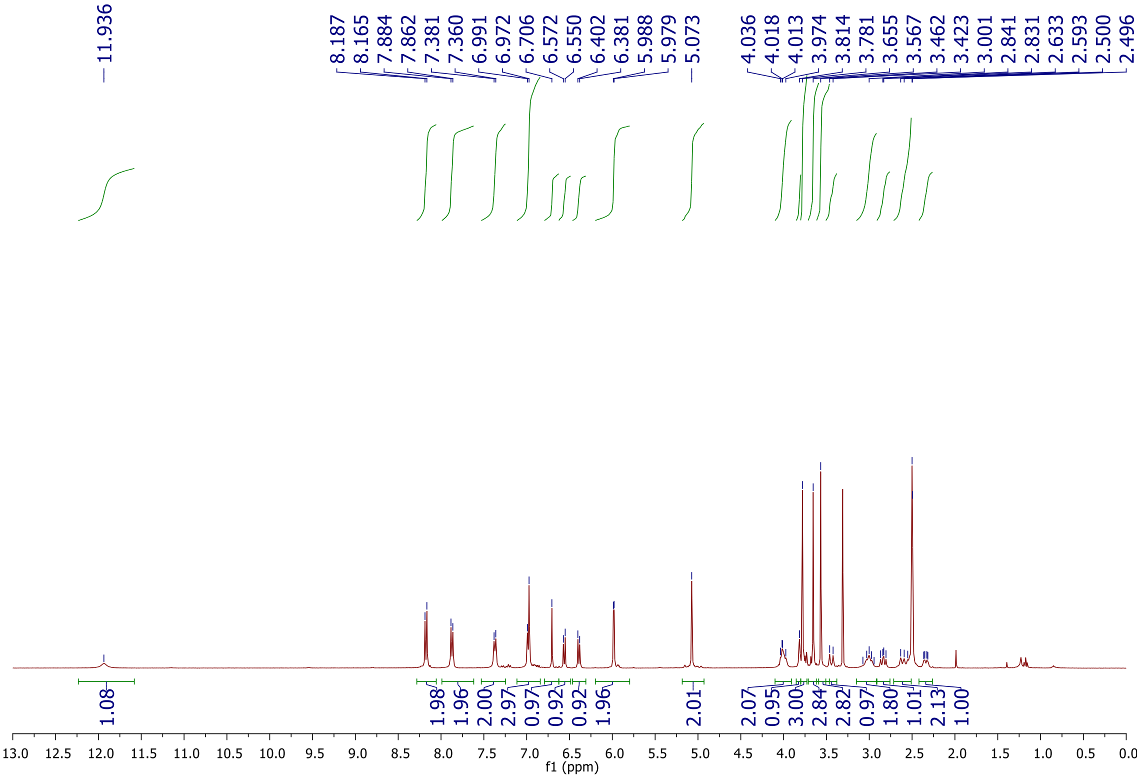

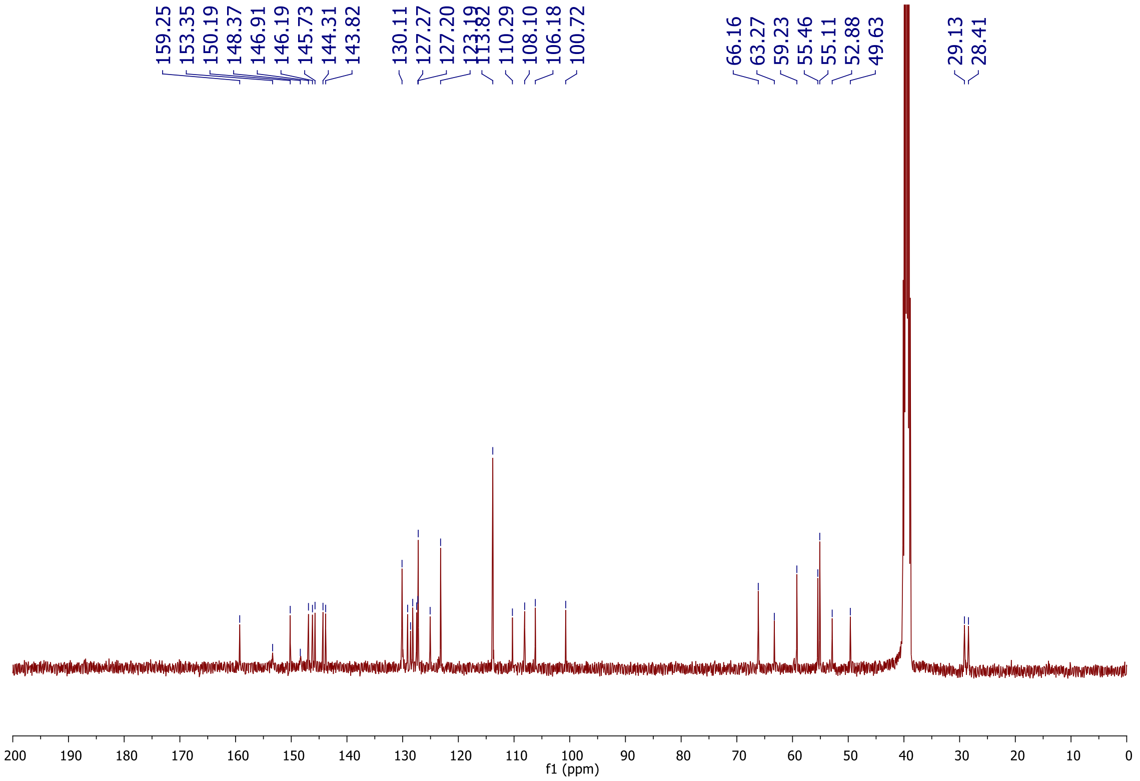

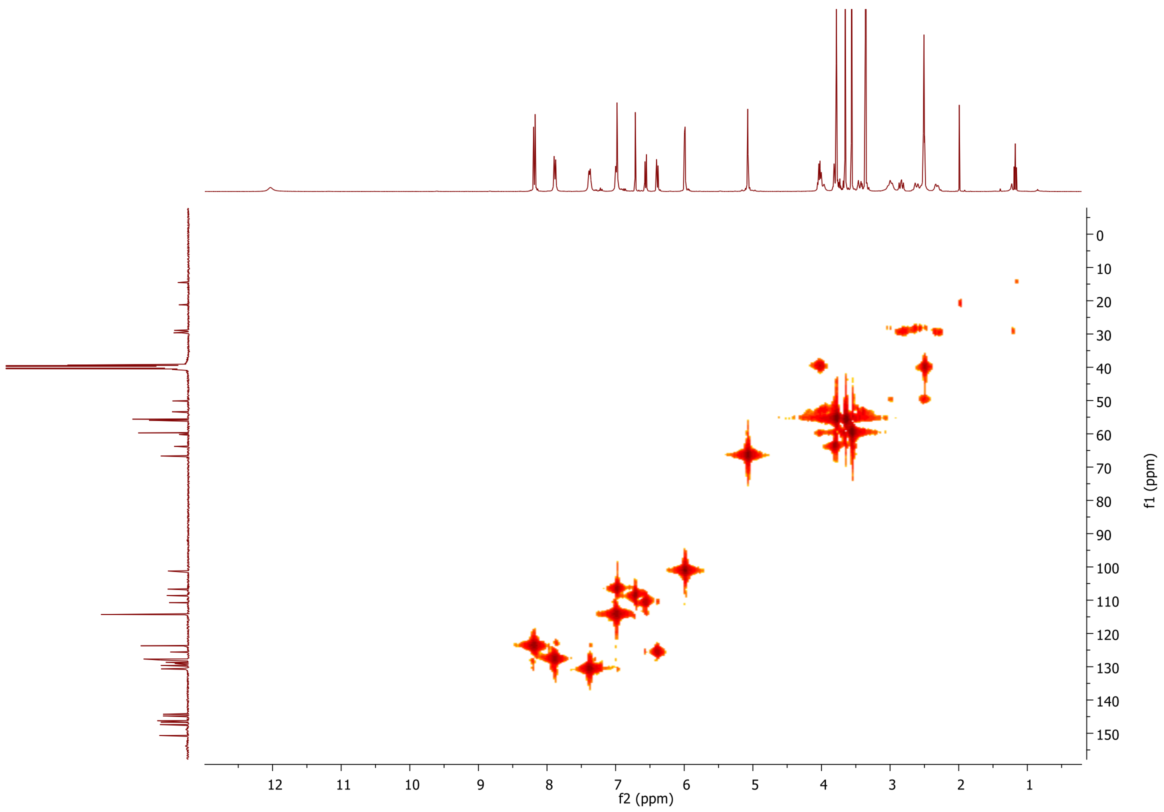

0
10
20
30
40
50
60
70
80
90
100
110
120
130
140
150
f1 (ppm)
12
11
10
9
8
7
6
5
4
3
2
1
f2 (ppm)

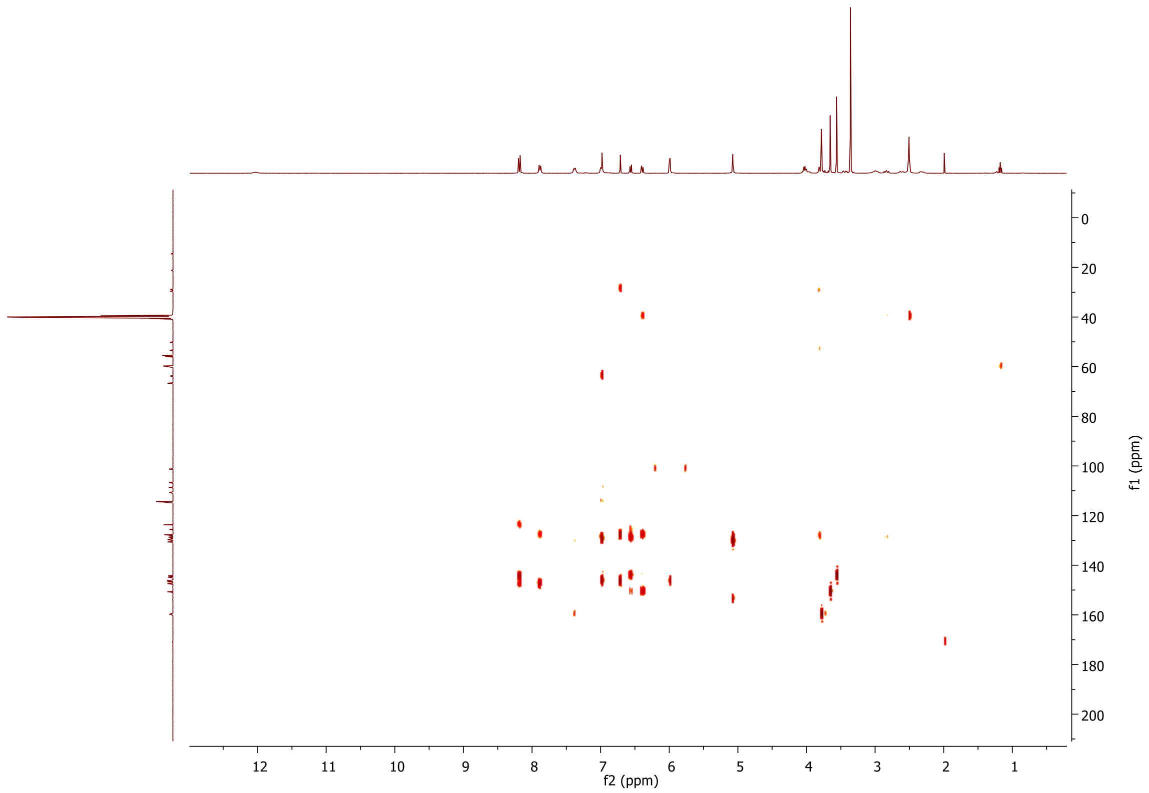

0
20
40
60
80
100
120
140
160
180
200
f1 (ppm)
12
11
10
9
8
7
6
5
4
3
2
1
f2 (ppm)

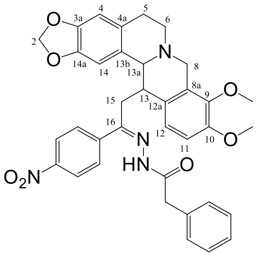

N'-(2-(9,10-Dimethoxy-6,8,13,13a-tetrahydro-5*H*-[1,3]dioxolo[4,5-g]isoquinolino[3,2-*a*]isoquinolin-13-yl)-1-(4-nitrophenyl)ethylidene)-2-phenylacetohydrazide (3j)

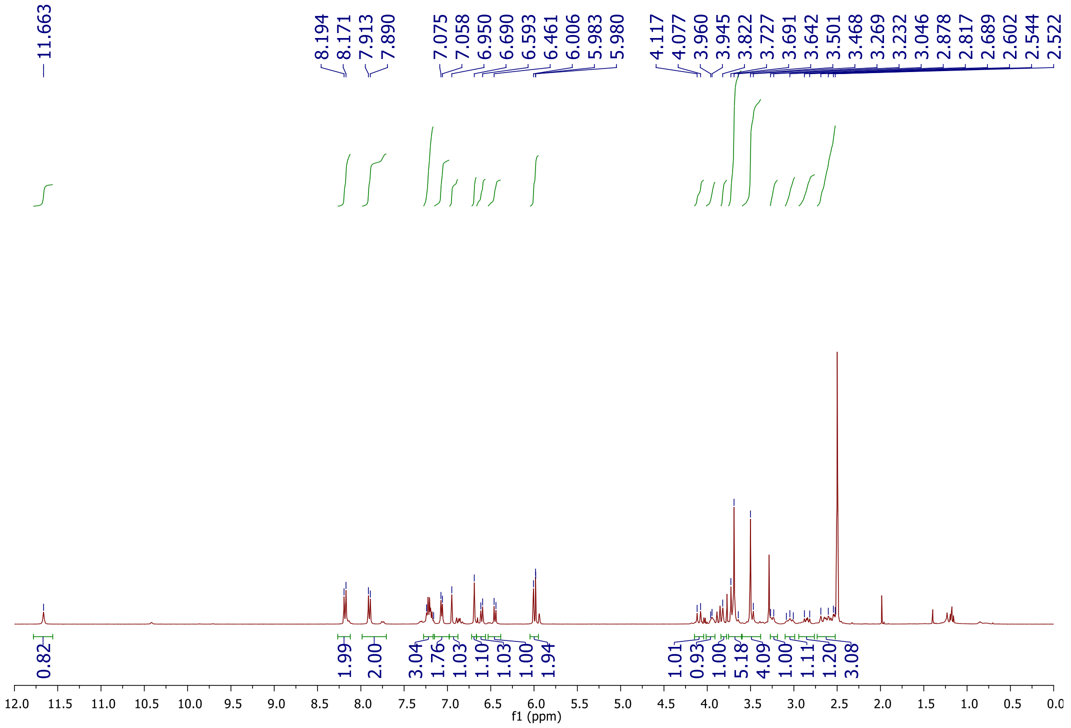

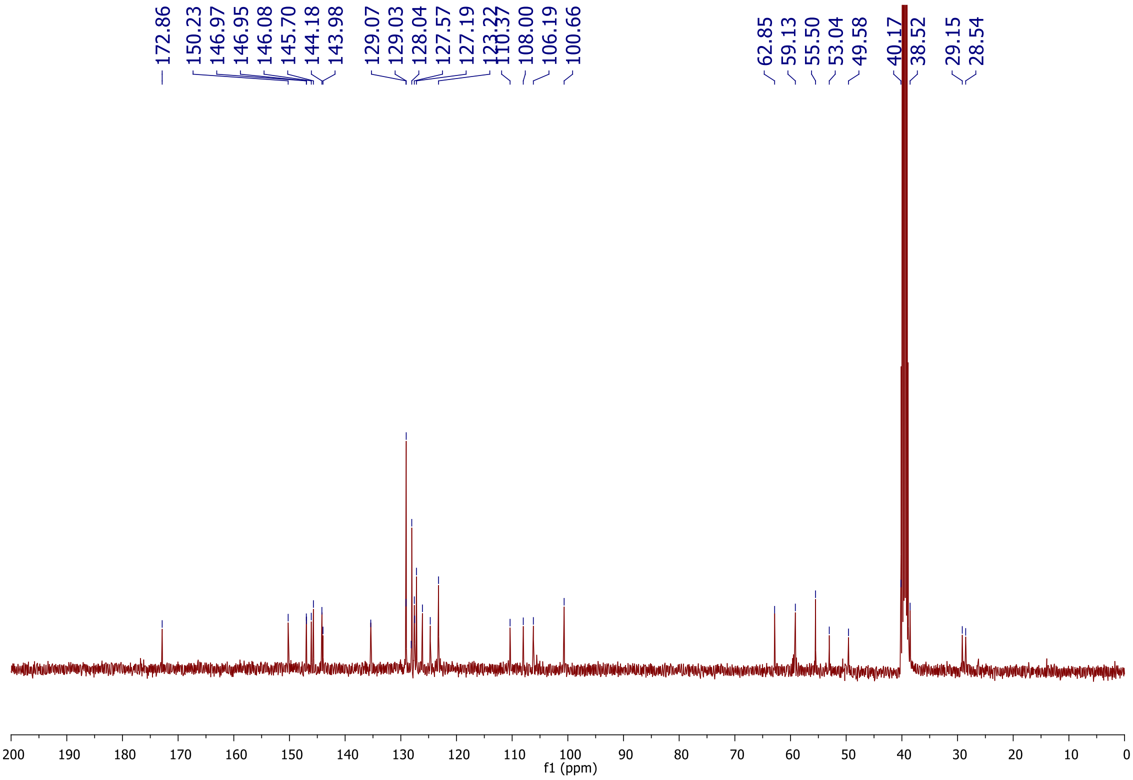

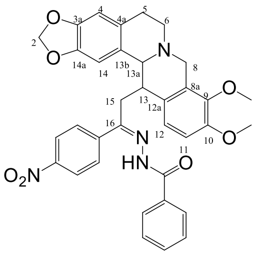

***N*'-(2-(9,10-Dimethoxy-6,8,13,13a-tetrahydro-5*H*-[1,3]dioxolo [4,5-*g*]isoquinolino[3,2-*a*]isoquinolin-13-yl)-1-(4-nitrophenyl) ehylidene)benzohydrazide (3k)**

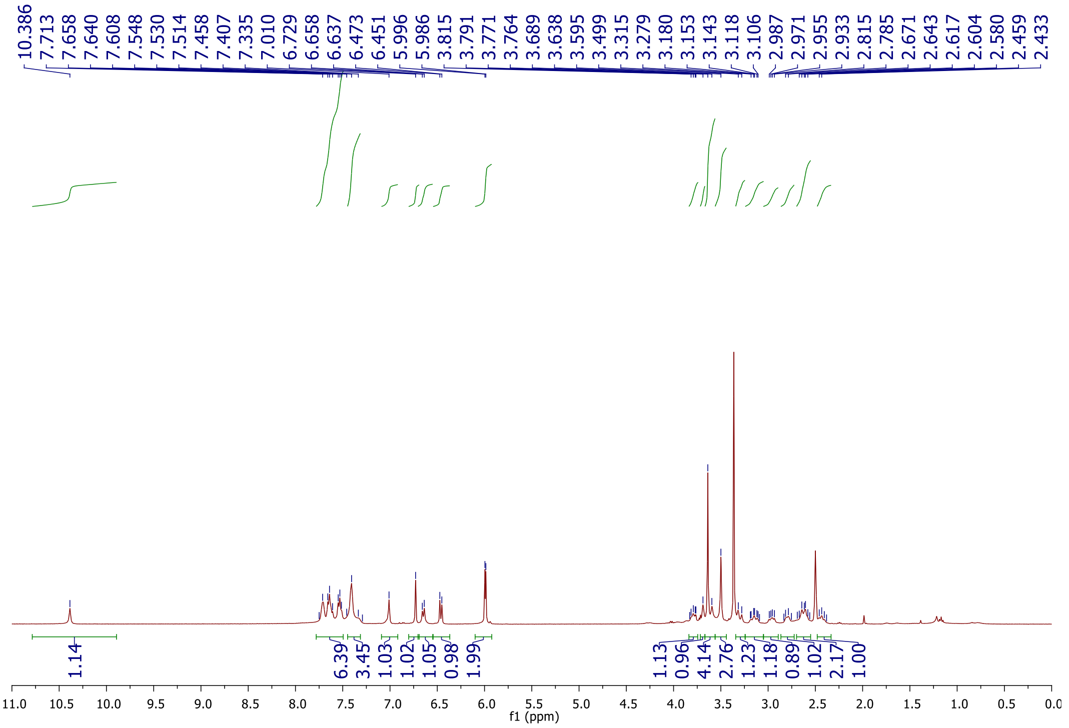

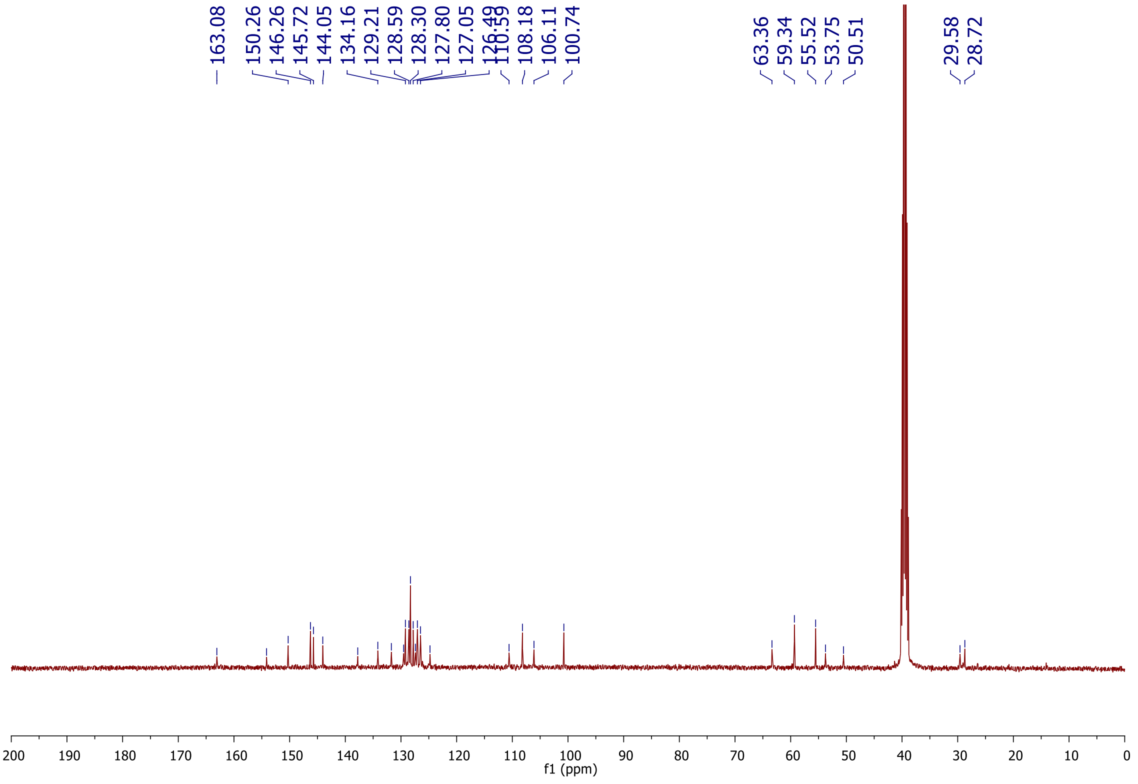

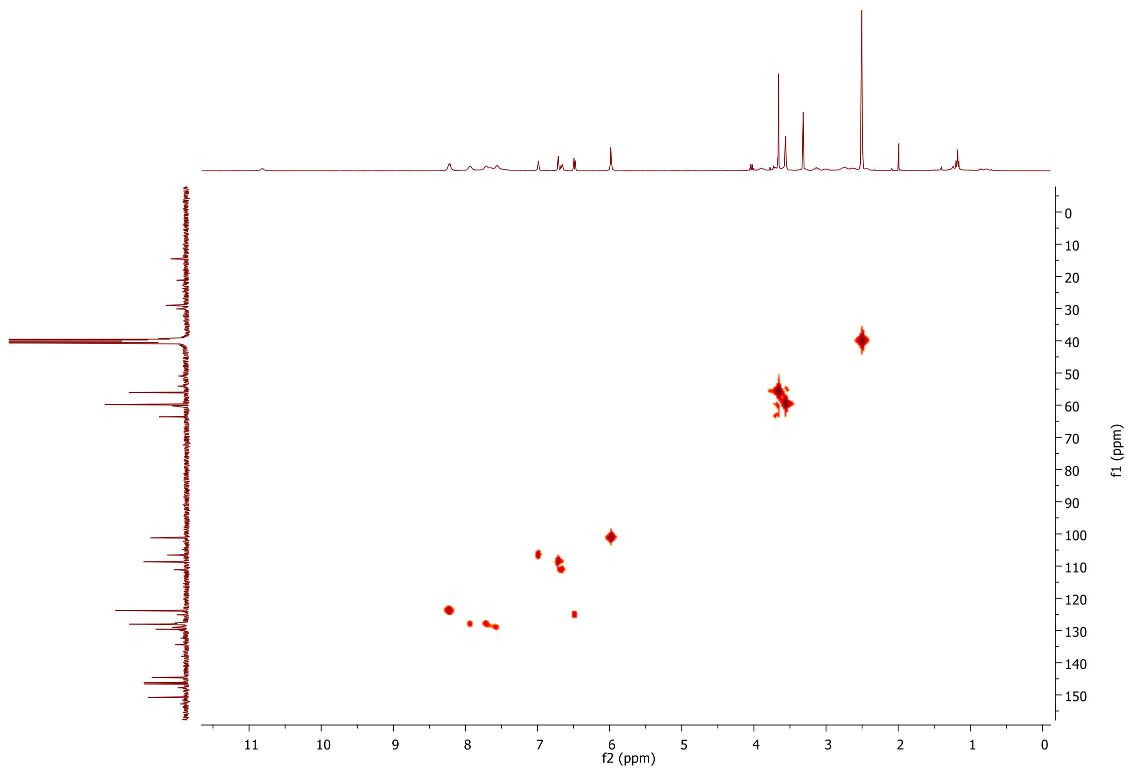
f2 (ppm)
f1 (ppm)
11
10
9
8
7
6
5
4
3
2
1
0
10
20
30
40
50
60
70
80
90
100
110
120
130
140
150

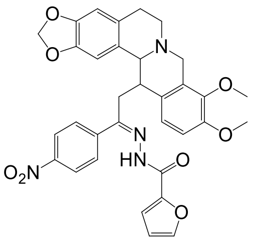

***N*'-(2-(9,10-Dimethoxy-6,8,13,13a-tetrahydro-5*H*-[1,3]dioxolo[4,5-*g*]isoquinolino[3,2-*a*]isoquinolin-13-yl)-1-(4-nitrophenyl) ethylidene)furan-2-carbohydrazide (3l)**

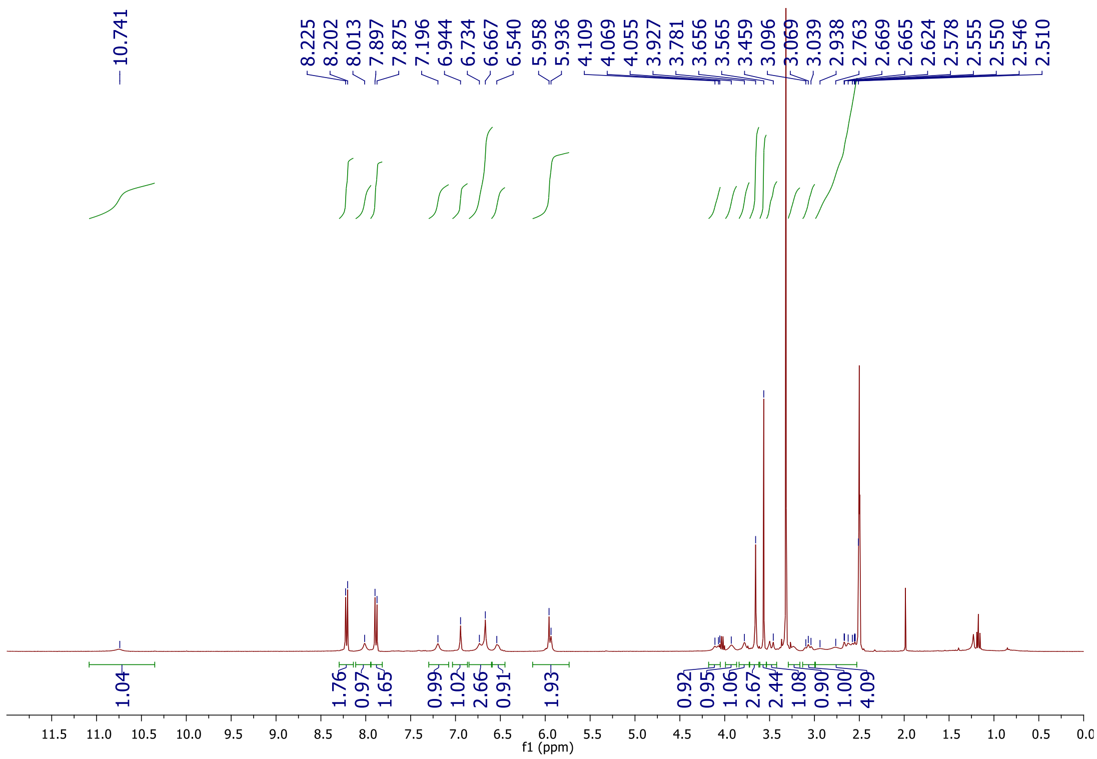

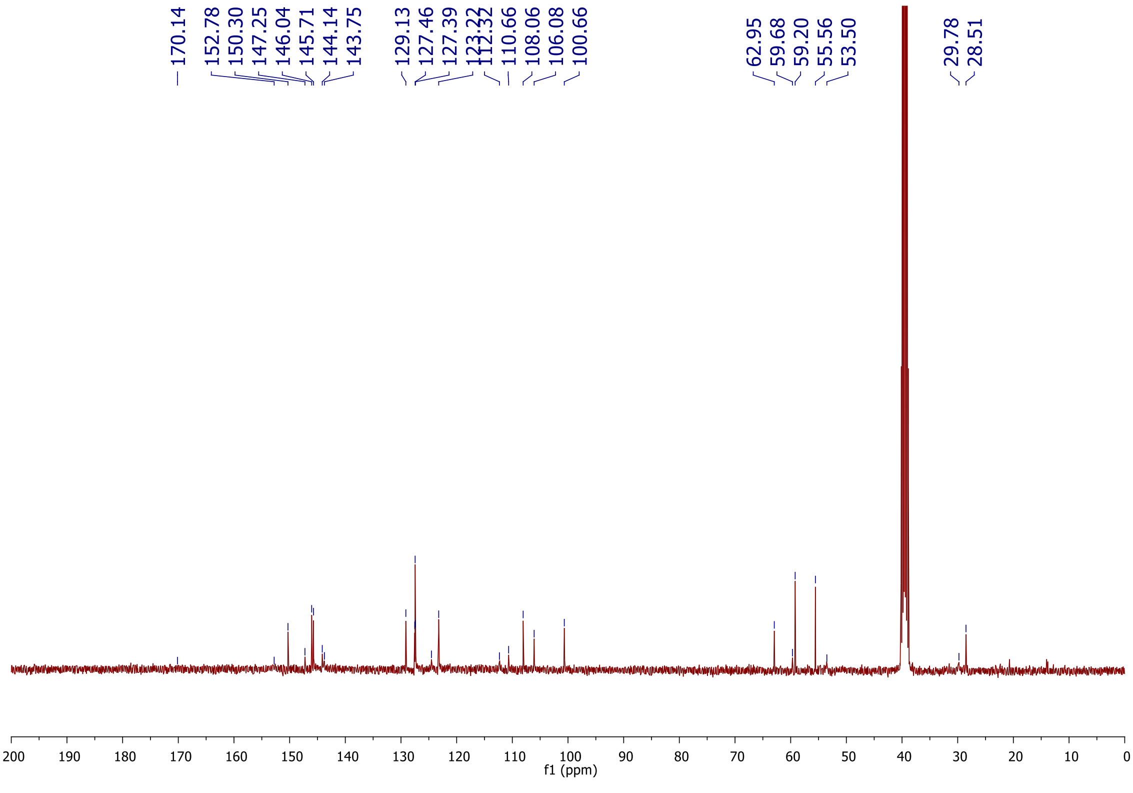

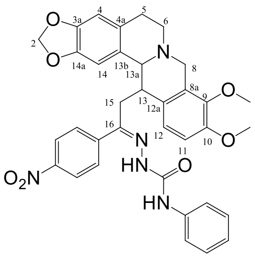

2-(2-(9,10-Dimethoxy-6,8,13,13a-tetrahydro-5*H*-[1,3]dioxolo[4,5-*g*]isoquinolino[3,2-*a*]isoquinolin-13-yl)-1-(4-nitrophenyl) ethylidene)-*N*-phnylhydrazinecarboxamide (3m)

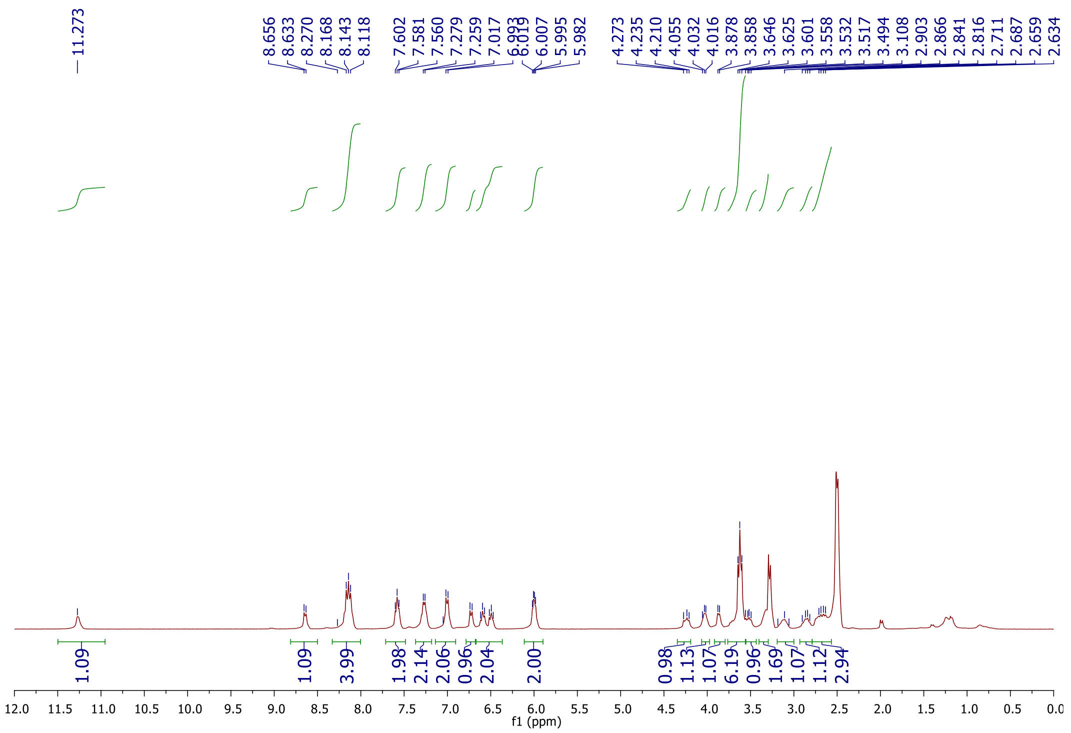

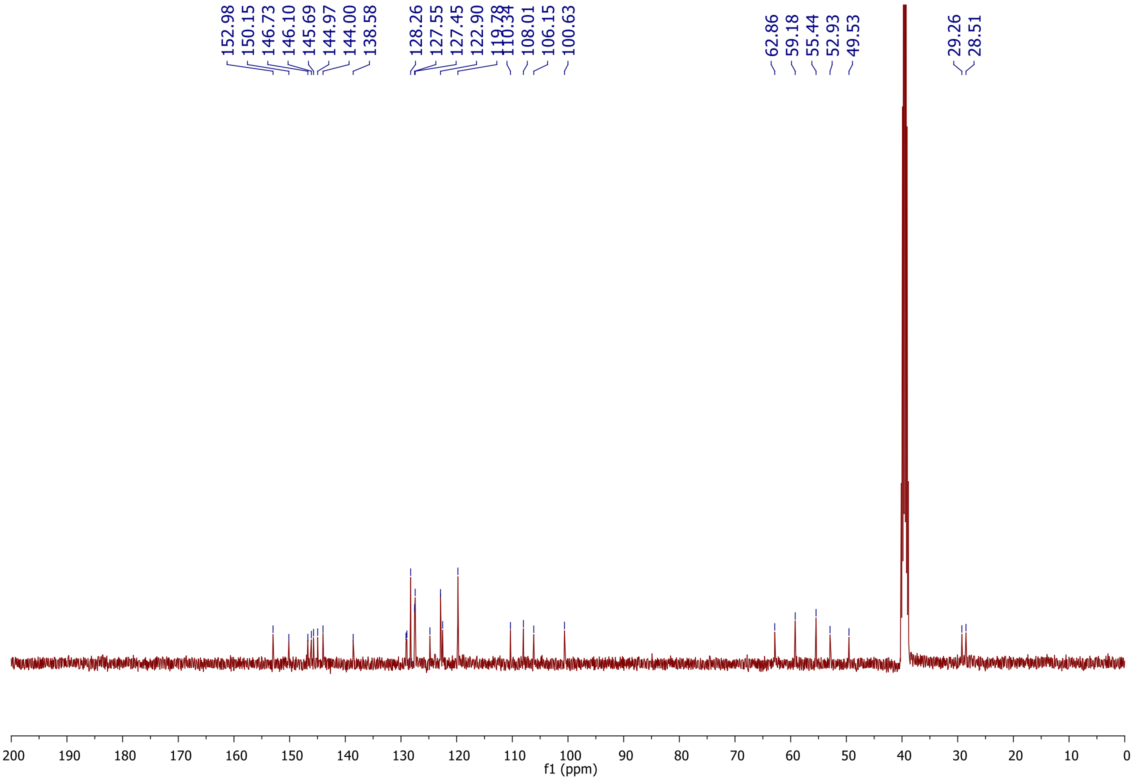

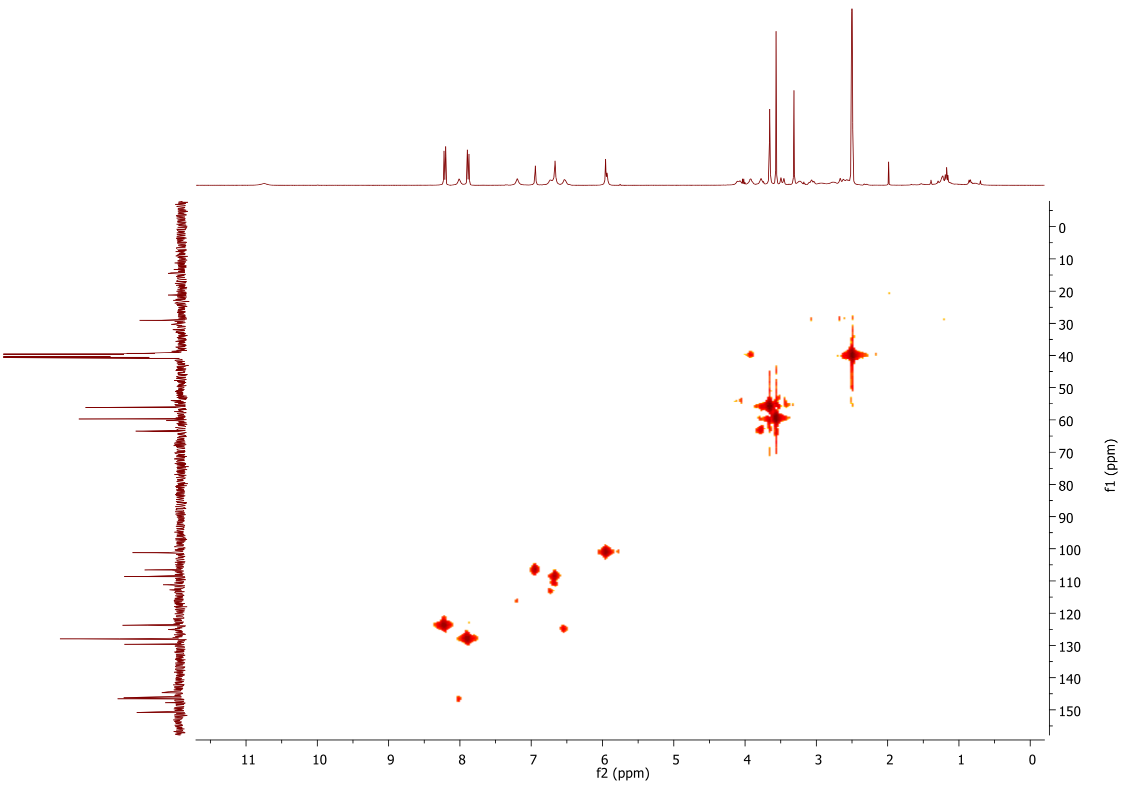

f1 (ppm)
0
10
20
30
40
50
60
70
80
90
100
110
120
130
140
150
f2 (ppm)
11
10
9
8
7
6
5
4
3
2
1
0

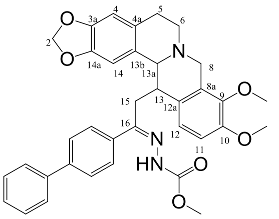

Methyl-2-(1-([1,1'-biphenyl]-4-yl)-2-(9,10-dimethoxy-6,8,13,13a-tetrahydro-5*H*-[1,3]dioxolo[4,5-*g*]isoquinolino[3,2-*a*]isoquinolin-13-yl)ethylidene)hydrazinecarboxylate (3n)

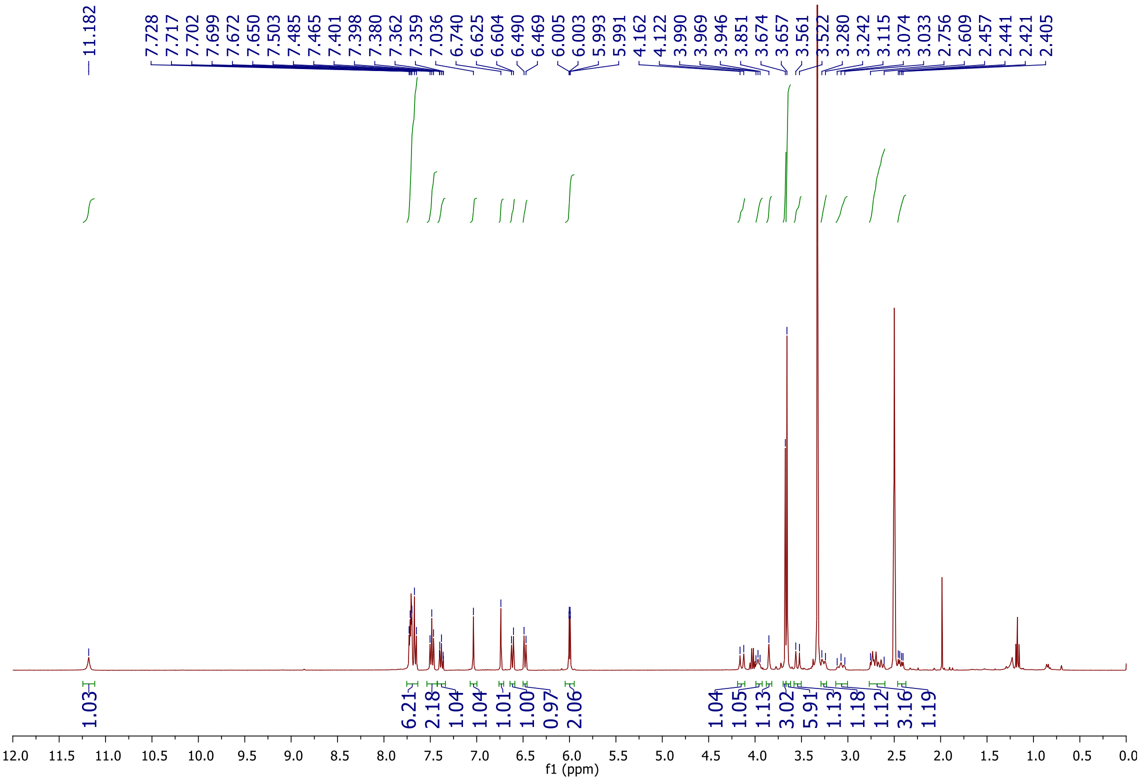

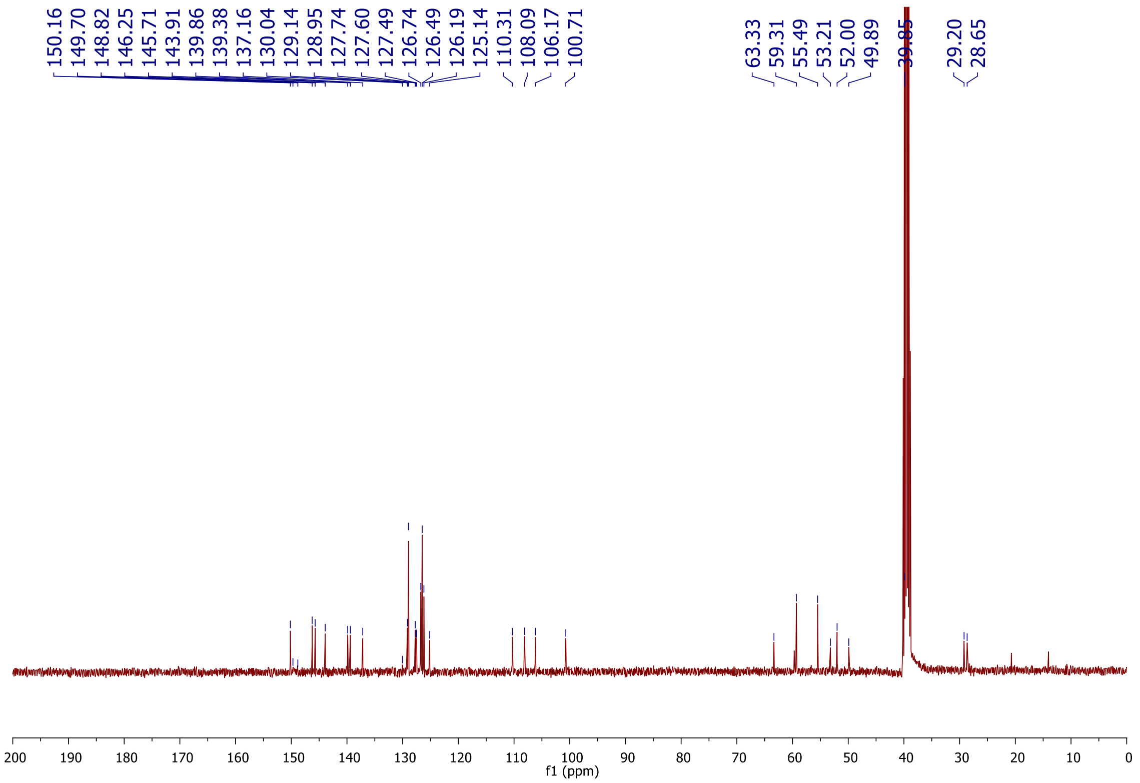

5. References

1. Ciccolini, C.; Mari, G.; Favi, G.; Mantellini, F.; De Crescentini, L. *Molecules* **2019**, *24*, 3785−3797.
2. Ferraroni, M.; Bazzicalupi, C.; Papi, F.; Fiorillo, G.; Guamán-Ortiz, L. M.; Nocentini, A.; Scovassi, A. I.; Lombardi, P.; Gratteri, P. *Chem. Asian J.* **2016**, *11*, 1107−1115.
3. Fu, S.; Xie, Y.; Tuo, J.; Wang, Y.; Zhu, W.; Wu, S.; Yan, G.; Hu, H. *Med. Chem. Commun.* **2015**, *6*, 164−173.
