# Supplementary material for: Synthesis of new dihydroberberine and tetrahydroberberine analogues and evaluation of their antiproliferative activity on NCI-H1975 cells
Source: Beilstein J Org Chem. 2020 Jul 6;16:1606–16. doi: 10.3762/bjoc.16.133 (PMC7356317; doi:10.3762/bjoc.16.133)
Supplement: File 2 — Antiproliferative evaluation of compounds 2a–n and 3a–g,i–n. [file Beilstein_J_Org_Chem-16-1606-s002.pdf]

BEILSTEIN JOURNAL OF ORGANIC CHEMISTRY

Supporting Information

for

Synthesis of new dihydroberberine and tetrahydroberberine analogues and evaluation of their antiproliferative activity on NCI-H1975 cells

Giacomo Mari, Lucia De Crescentini, Serena Benedetti, Francesco Palma, Stefania Santeusanio and Fabio Mantellini

Beilstein J. Org. Chem. **2020,** *16,* 1606–1616. doi:10.3762/bjoc.16.133

Antiproliferative evaluation of compounds 2a–n and 3a–g,i–n

License and Terms: This is a supporting information file under the terms of the Creative Commons Attribution License (http://creativecommons.org/licenses/by/4.0). Please note that the reuse, redistribution and reproduction in particular requires that the authors and source are credited.
The license is subject to the *Beilstein Journal of Organic Chemistry* terms and conditions: (https://www.beilstein-journals.org/bjoc)

Table of contents

1. Cell proliferation assays. S2

2. WST-8 assay. S2

3. Sulforhodamine B assay. S3

4. Statistical analysis. S3

5. References. S3

1. Cell proliferation assays.

The non-small cell lung cancer (NSCLC) cell line NCI-H1975 (ICLC, Genova) was grown in DMEM medium supplemented with 10% fetal bovine serum, 1% L-glutamine, and 1% penicillin/streptomycin 100 U/mL (Sigma-Aldrich, Milan, Italy) and maintained in a CO_2 incubator at 37 °C and 5% CO_2. The effects of hydrazone-DHBERs **2a–n** and hydrazone-THBERs **3a–g,i–n** on cancer cell proliferation were analyzed by WST-8 and sulforhodamine B (SRB) assays, estimating cell metabolic activity and total protein content, respectively.[1] Test compounds were solubilized in dimethyl sulfoxide (DMSO) at the concentration 50 mM and then diluted at 25 µM in complete medium (final DMSO concentration 0.05%). DHBER and THBER were also tested at the same concentration as reference molecules for hydrazone-DHBERs and hydrazone-THBERs, respectively.

2. WST-8 assay.

The WST-8 assay (Sigma-Aldrich, Milan, Italy) is a colorimetric test based on the reduction of the tetrazolium salt WST-8 [2-(2-methoxy-4-nitrophenyl)-3-(4-nitrophenyl)-5-(2,4-disulfophenyl)-2*H*-tetrazolium, monosodium salt] by cellular dehydrogenases in viable cells.[2] Briefly, cells (5000/well) were seeded in clear 96-well plates and then treated with the test compounds (25 µM) or vehicle (0.05% DMSO, untreated control). After 24 and 48 of incubation, test compounds were removed thus to avoid possible interferences with the assay, WST-8 reagent in DMEM was added to each well, and cells were further incubated at 37 °C up to 4 h. Color development was monitored at 450 nm in a multiwell plate reader (Thermo Fisher Scientific, Milan, Italy). Data were expressed as percentage versus untreated cells.

3. Sulforhodamine B assay

The SRB assay is based on the ability of SRB dye to bind basic protein amino acid residues. The amount of dye incorporated by the cells gives an estimation of total protein mass, which is directly proportional to cell number.[3] In detail, cells (5000/well) were seeded in 96-well plates and then treated with the test compounds (25 µM) or vehicle (0.05% DMSO, untreated control) for 24 and 48. At the end of the treatment, test compounds were removed, cells were fixed using 100 µl/well of 50% aqueous trichloroacetic acid for 1 h at 4 °C, rinsed with water and incubated for 30 min with 50 µl/well SRB solution (0.4%) (Sigma-Aldrich, St. Louis, USA). After rinsing with 1% acetic acid and solubilizing in 10 mM Tris for 10 min, the absorbance of each well was measured in a microplate reader (Thermo Fisher Scientific, Milan, Italy) at 570 nm. Data were expressed as percentage versus untreated cells.

4. Statistical analysis

Results were presented as mean ± SD of three independent experiments. Differences between means were analyzed by one-way ANOVA followed by Tukey post-hoc test (SPSS software, IBM). A P value <0.05 was considered statistically significant.

5. References

1. Menyhárt, O.; Harami-Papp, H.; Sukumar, S.; Schäfer, R.; Magnani, L.; de Barrios, O.; Győrffy, B. *Biochim. Biophys. Acta* **2016,** *1866*, 300–319.
2. Mari, G.; Catalani, S.; Antonini, E.; De Crescentini, L.; Mantellini, F.; Santeusanio, S.; Lombardi, P.; Amicucci, A.; Battistelli, S.; Benedetti, S.; Palma, F. *Bioorg. Med. Chem.* **2018**, *26*, 5037–5044.
3. Farabegoli, F.; Scarpa, E. S.; Frati, A.; Serafini, G.; Papi, A.; Spisni, E.; Antonini, E.; Benedetti, S.; Ninfali, P. *Food Chem.* **2017**, *218*, 356–364.
